# Computational modeling of complex bioenergetic mechanisms that modulate CD4+ T cell effector and regulatory functions

**DOI:** 10.1038/s41540-022-00263-4

**Published:** 2022-11-22

**Authors:** Ryan Baker, Raquel Hontecillas, Nuria Tubau-Juni, Andrew J. Leber, Shiv Kale, Josep Bassaganya-Riera

**Affiliations:** NIMML Institute, Blacksburg, VA 24061 USA

**Keywords:** Bioenergetics, Biochemical networks, Differential equations

## Abstract

We built a computational model of complex mechanisms at the intersection of immunity and metabolism that regulate CD4+ T cell effector and regulatory functions by using coupled ordinary differential equations. The model provides an improved understanding of how CD4+ T cells are shaping the immune response during *Clostridioides difficile* infection (CDI), and how they may be targeted pharmacologically to produce a more robust regulatory (Treg) response, which is associated with improved disease outcomes during CDI and other diseases. LANCL2 activation during CDI decreased the effector response, increased regulatory response, and elicited metabolic changes that favored Treg. Interestingly, LANCL2 activation provided greater immune and metabolic modulation compared to the addition of exogenous IL-2. Additionally, we identified gluconeogenesis via PEPCK-M as potentially responsible for increased immunosuppressive behavior in Treg cells. The model can perturb immune signaling and metabolism within a CD4+ T cell and obtain clinically relevant outcomes that help identify novel drug targets for infectious, autoimmune, metabolic, and neurodegenerative diseases.

## Introduction

*Clostridioides difficile* is an anerobic, gram-positive bacterium responsible for opportunistic infections in the gastrointestinal tract (GI) which can lead to nosocomial diarrhea, pseudomembranous colitis, or even death^1^. According to the Centers for Disease Control (CDC), *C. difficile* infection (CDI) affects over 450,000 people every year in the U.S. alone, with about 29,000 deaths within one month from the diagnosis^2^. *C. difficile* facilitates disease and more specifically tissue damage in part through the production of toxin A (TcdA) and toxin B (TcdB), which are glucosyltransferases that catalyze the glucosylation of GTP-binding proteins Rho, Rac, and Cdc42 thereby modifying signal transduction pathways that lead to cell death^3^. Host immunity to *C. difficile* is orchestrated through temporally balanced T effector (Th1/Th17) and T regulatory (Treg) responses^4^. For instance, IL-17 has been reported as a key cytokine in inducing severe inflammation, promoting further neutrophil recruitment^5^. Indeed, Th17 responses are dominant during the peak of inflammation, while Treg cells are upregulated throughout the recovery/resolution phase in preclinical models^6^. Similarly, increases in CD4+Foxp3+ Tregs populations by *Bacteroides fragilis* treatment, believed to alter metabolism, induces IL-10 and TGF-β production and mucosal tolerance to CDI^7^. Thus, the increase in Tregs may also be beneficial for treating both CDI and reducing recurrent CDI. Furthermore, functional Tregs play crucial roles in providing protection against autoimmune disease such as ulcerative colitis, Crohn’s disease, and lupus as well as neurogenerative diseases such as Alzheimer’s disease. Here, we present our computational modeling efforts to study CD4+ T cells in general and Tregs in particular. More specifically, we use our computational model to evaluate whether activation of host LANCL2, a novel therapeutic target, reduces immunopathogenic Th17 responses and enhances protective Treg responses. Our in silico efforts also provide a unique window to the changes in metabolic processes that shape phenotype and function of CD4+ T cells in general and Treg in particular during CDI.

CD4+ T cells subsets have distinct metabolic profiles. For instance, Treg cells have reduced glucose cellular uptake, elevated fatty acid oxidation, and higher levels of oxidative phosphorylation^8,9^. In contrast, pathogenic effector cells (e.g., Th1 and Th17) tend to have heightened glucose import to the cells and a Warburg Effect driven metabolism, with high glycolytic activity, high lactate production, and reduced oxidative phosphorylation within the mitochondria^8,10^. The TCA cycle is an important source of NADH for energy production, but many other metabolic pathways flow through sections of the TCA cycle. Pathways such as gluconeogenesis, glutaminolysis, fatty acid synthesis, and the malate-aspartate shuttle all depend on portions of the TCA cycle and provide mechanisms for balancing metabolites in the TCA cycle and other pathways, providing alternative energy sources, and building blocks for cell proliferation and function^11^. Many of these metabolic pathways are necessary for CD4+ T cell differentiation and function, but the mechanistic linkages between immunity and metabolism remain poorly understood. Indeed, both immunity and metabolism are complex massively and dynamically interacting systems, and given their huge complexity, it can be difficult to accurately pinpoint the underlying molecular mechanisms that mediate functional and phenotypic outcomes.

An illustrative example of an emerging prototypical pathway in the intersection of immunity and metabolism and a drug target is LANCL2, an immunometabolic regulator whose natural ligand, abscisic acid (ABA), is a plant derived phytohormone that is important for glycemic control in humans^12,13^. Named after their sequence similarity to bacterial lanthionine synthase C-like proteins, the mammalian homologs LANCL1, LANCL2, and LANCL3 were found to not contribute to lanthionine synthesis^14^. Instead, LANCL2 was found to bind ABA and a variety of synthetic molecules resulting in the increased glucose uptake via the increased expression of GLUT1/4^13^. Activation of LANCL2 also has been shown to stimulate metabolite entrance into the TCA cycle via increased enzymatic activity of pyruvate dehydrogenase (PDH)^15^. Activation of LANCL2 also increases STAT5 phosphorylation and FOXP3 expression via IL-2 receptor interaction, leading to an increase in Treg cells and a decrease in Th17 cell populations^16^. Conversely, loss of LANCL2 results in increased lactate production via glycolysis specifically through the increasing hexokinase and lactate dehydrogenase (LDH) activity in CD4+ T cells^17^. In silico modeling efforts have made substantial gains in the past 10 years to describe functional relationships in biochemistry, cell signaling pathways, and cellular processes^18–20^. Previously implemented in silico models that function on many levels of immunity, including macrophage differentiation^21^, systems-level response of the immune system to CDI^6^, co-infections^22^, hybrid agent-based/ODE modeling of *Helicobacter pylori* infection of the gut^23^, comprehensive mathematical models of metabolism^24,25^, and a dynamic model of CD4+ T cell differentiation^26^ provide a foundation upon which this work is built. Our recent modeling efforts presented here have incorporated components of metabolism as critical modulators of immune signaling and CD4+ T cell differentiation. Given the dual roles of LANCL2 and the increasing importance of metabolism on regulating immune signaling, modeling LANCL2 effect on CD4+ T cell differentiation during infection such as CDI may prove to be valuable in defining new therapeutic opportunities. Metabolic regulation of immunity and modulation of metabolism by immunological changes has become a focus of substantial research efforts^11^. The addition of metabolic regulation as well as LANCL2 into our model of CD4+ T cell differentiation have provided us mechanistic insights into how LANCL2 activation reduces Th17 and increases iTreg CD4+ T cell phenotypes via LANCL2 as well as insight into the importance of citrate transport and lactate accumulation in shaping regulatory and effector responses. The use of computational modeling in understanding how to pharmacologically manipulate the intersection of immunity and metabolism to influence the balance of effector versus Treg cells has applications in the treatment of infectious, autoimmune, metabolic, and neurodegenerative diseases.

## Results

### Creation of ODE model of CD4+ T cell differentiation and metabolism

**Fig. 1:**
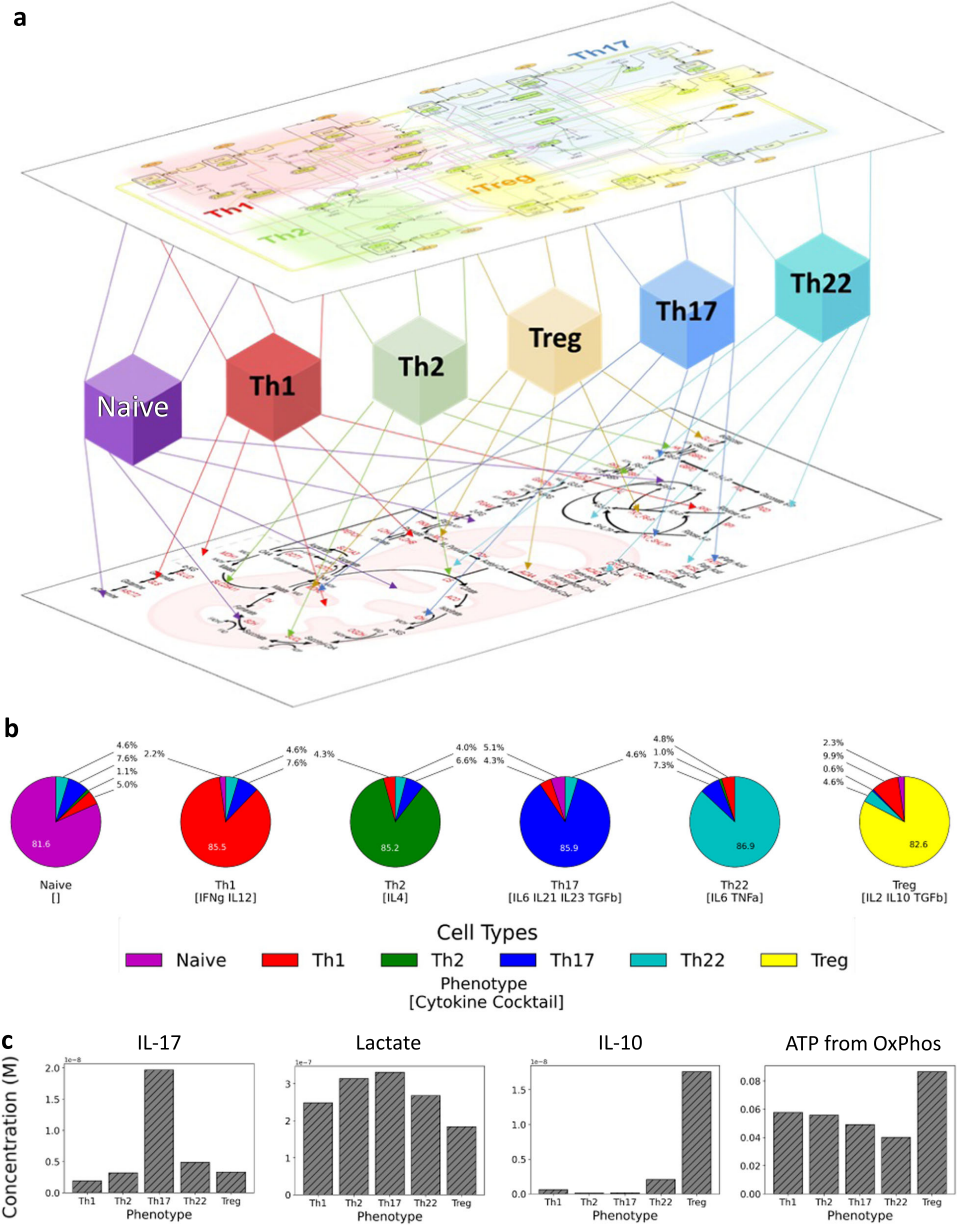
Calibration and assessment of immuno-metabolic model of CD4+ T cell differentiation. **a** Graphical representation of the layered approach to the model. Cytokine concentrations are inputted into the model to determine a portion of CD4+ T cell differentiation. Type and composition of differentiated CD4+ T cells determine metabolism. Metabolism feeds back into immune signaling and can impact CD4+ T cell differentiation at specific junctures. **b** Predicted CD4+ T cell differentiation based on defined inputs for a given state. **c** Generated cytokine values (IL-10 and IL-17) and metabolite values (intracellular lactate, ATP from oxidative phosphorylation) for each CD4+ phenotype using pre-defined input cytokine cocktails.

We created an ODE model of CD4+ T cell bioenergetics aimed at predicting differentiation of Naïve CD4+ T cells into a terminal state from input cytokine concentration and metabolites (Fig. 1a). The model consists of 66 enzymes, 75 immune modulators, and 108 metabolites that cover metabolic and immunological processes at varying granularities. The differentiated phenotypes encompass Th1 (T-bet activated), Th2 (GATA3 activated), Th17 (RORγ-t activated), Th22 (AhR activated), and Treg (FOXP3 activated). The underlying structure of the cytokine signaling pathways were expanded upon from our prior work in Carbo et al.^26^ to now include components of Th22 differentiation as well as a more up-to-date understanding of molecular signaling pathways within CD4+ T cells. The metabolic portion of the model encompasses glycolysis, gluconeogenesis, the pentose phosphate pathway (PPP), tricarboxylic acid (TCA) cycle, oxidative phosphorylation (OxPhos), fatty acid oxidation, glutaminolysis, and the malate-aspartate shuttle. Enzyme kinetics and reactions for the model were derived from primary literature and Sabio-RK^27^ when available and relevant. Metabolic enzyme concentrations for each of the CD4+ T cell phenotypes were extracted and normalized from a single cell RNA-Seq study^28^ and used to determine enzyme concentrations based on CD4+ T cell phenotype. Metabolic enzyme concentrations ([*E*]) are determined by a weighted average, calculated using the phenotype value of each cell type (*p*_*i*_) and the metabolic enzyme concentration ([*e*]_*i*_) of each cell type derived from the CD4+ T cell RNAseq study^28^ where *n* is equal to the number of cell types.

$$\left[ E \right] = \mathop {\sum }\limits_{i = 1}^n p_i\left[ e \right]_i$$

These two layers of the model are connected by a convergence layer consisting of nodes representing each of the CD4+ T cell phenotypes. The model determines the relative composition of the CD4+ T cell population (*p*_*i*_) based on input cytokine concentrations via the immune signaling portion of the model. The model contains five transcription factors that each correspond to a T cell phenotype. The level of activation of each transcription factor is transformed using a sigmoidal function to return five intermediate phenotype values from 0 to 1. If the sum of the *p* values exceed 1, the values are normalized such that they sum to 1. The Naïve phenotype value is assigned last as the difference between 1 and the sum of all other phenotype values. The relative CD4+ T cell composition is then used to determine expression of metabolic enzymes thereby allowing for regulation of metabolism. A subset of metabolites also can modulate aspects of immune signaling thereby impacting CD4+ T cell differentiation though a feedback loop. These interactions of metabolism on immune signaling were identified through electronic literature mining followed by manual curation (Supplementary Figs. 1, 2). Ultimately, the model is designed to allow us to interrogate how naïve CD4+ T cells respond to a complex cytokine milieu as well as how modulation of metabolism and signal transduction components impacts differentiation.

### Model validation shows appropriate differentiation to cytokine cocktails

We utilized experimentally defined cytokine cocktails and expected CD4+ T cell differentiation as the independent and dependent variables to calibrate the model respectively (Supplementary Table 1). Due to the high number of parameters within the immune layer of the model that required estimation, we performed 310 parallel calibration runs using a high-powered cluster computing resource to perform parameter estimation using the particle swarm algorithm^29^. We implemented a parameter set with the smallest root-mean-square deviation (RMSD) from the validation dataset to create our final model. It is important to reiterate parameter calibrations only occurred for values that were for the immunological portion of the model as metabolic enzyme kinetics were derived from literature. The calibration with the lowest RMSD value was used for subsequent manual curation to incorporate realistic model parameters values. To further assess our calibrated model, we re-determined differentiation of CD4+ T cells based on our pre-defined input cytokine cocktails. Our results indicate that naïve CD4+ T cells appropriately differentiate into the predicted phenotype for a given cytokine cocktail (Fig. 1b). Analysis of metabolism and produced cytokines also indicate hallmark features such as the relative increase of glycolysis shown by enhanced lactate production increased lactate production, and the increase in mitochondrial function (mitochondrial ATP production) and fatty acid oxidation for Tregs (Fig. 1c). The production of IL-17 and IL-10 were also specific to their respective T cell phenotypes upon differentiation (Th17 and Treg) (Fig. 1c).

### In silico characterization of resolving *C. difficile* infection

**Fig. 2:**
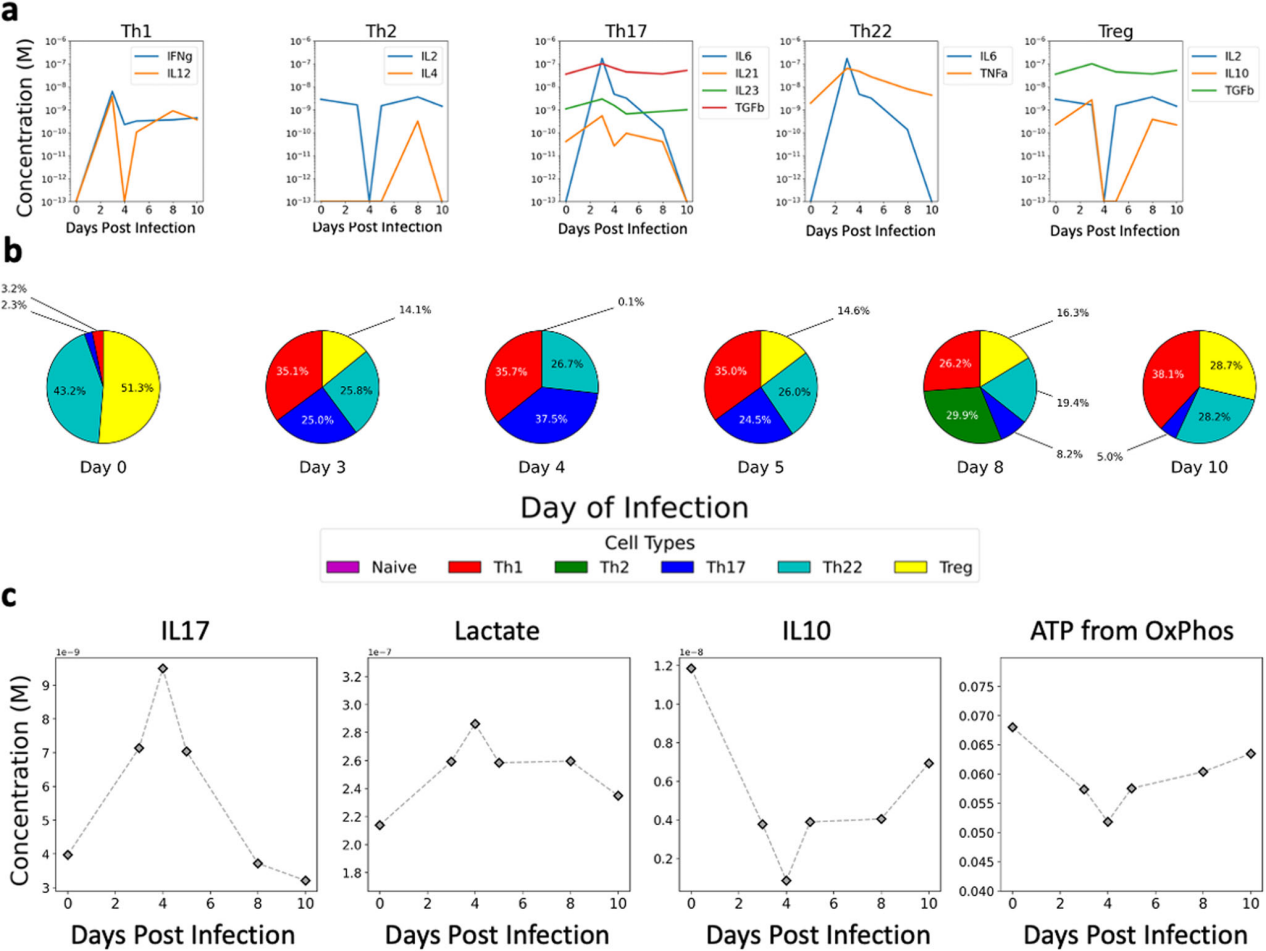
Predicted CD4+ T cell differentiation during *Clostridioides difficile* infection based on gene expression from RNA-Seq expression. **a** Gene expression of input cytokines from *Clostridioides difficile* infection associated with each CD4+ differentiated state prior to infection and on days 5 and 10 post infection. **b** Predicted CD4+ T cell differentiation based on cytokine gene expression on a given day pre or post infection. **c** Production of cytokines (IL-17 and IL-10) and metabolites (intracellular lactate accumulation and ATP from Oxidative Phosphorylation).

We chose to initially apply our immunometabolic model of CD4+ T cell differentiation on a time course study of resolving CDI given the clinical relevance of *C. difficile* as well as the importance of host CD4+ T cell responses during and after infection. The data set consisted of a temporal RNA-Seq analysis of mouse colons on Days 0, 3, 4, 5, and 10 post inoculation with *C. difficile*, as well as flow cytometry analysis of recruited immune cell populations. Input cytokine concentrations were derived from normalized and transformed RNA-Seq data (Fig. 2a). Based on these inputs, our model indicated an initial mixture of primarily CD4+ Tregs (51.3%), Th1 (3.2%), Th17 (2.3%), and Th22 (43.2%) cells prior to infection with *C. difficile* (Fig. 2b). These results are in accord with our prior results showing primarily a CD4+ Treg and with lower amounts of Th1 and Th17 populations in unchallenged colons^6^. Three days after infection, the model indicated Treg population had decreased to a third of the pre-infection levels (14.1%) and the population consisted of a mixture of Th1 (35.1%), Th17 (25.0%), and Th22 (25.8%) cells (Fig. 2b). Due to a decrease in IL-2 concentration in the input data, Treg levels dropped close to zero (0.1%) on day 4, with an increased Th17 response (37.5%). A similar response to day 3 was observed on day 5 post inoculation. By day 10, CD4+ Tregs had re-emerged as a dominant cell population and consisted of 28.7% of the overall population and Th22 (28.2%), Th1 (38.1%), and Th17 (5.0%) represented the remainder. Analysis of lactate production followed suit with IL-17 production by CD4+ T cells and ATP generation via OxPhos correlated with IL-10 production by CD4+ T cells (Fig. 2c). Our analysis suggests a return to pre-infection state by day 10 post infection, while a strong effector response was observed on days 3,4, and 5. Our in-silico analysis suggested a mixed Th1/Th17/Th22 response during infection and a recovery phase by day 10 that consisted of primarily Treg cells.

**Fig. 3:**
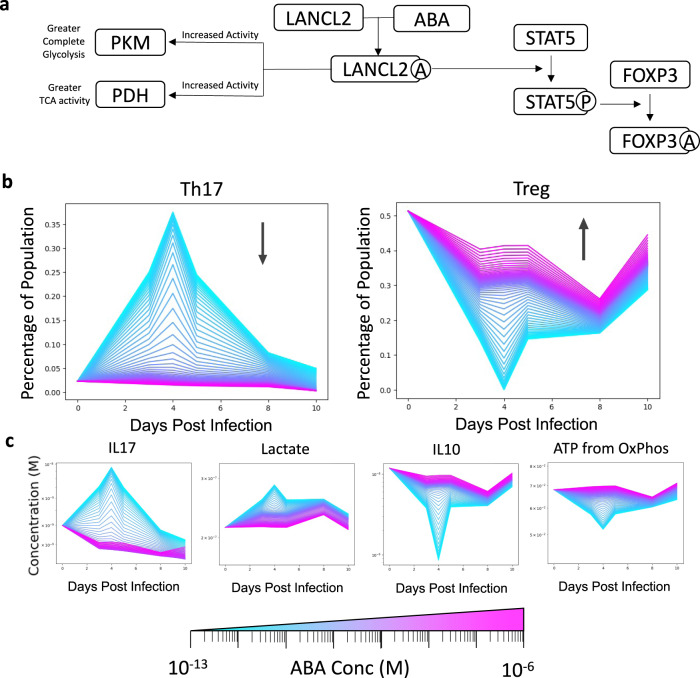
The effect of varying concentrations of ABA on CD4+ T cell differentiation during *Clostridioides difficile* infection. **a** Gene expression of input cytokines from *Clostridioides difficile* infection associated with each CD4+ differentiated state prior to infection and on days 5 and 10 post infection. **a** Known connections of LANCL2 to immune signaling and metabolism. **b** Increase in percentage of Treg and decrease in percentage of Th17 cells in the presence of varying concentrations of ABA (10^−7^ to 1 μΜ). **c** Increase in amount of produced IL-10 and ATP from oxidative phosphorylation and decreased IL-17 production and accumulated lactate in the presence of varying concentrations of ABA.

An increase in Treg cells and a decrease in Th17 cells is thought to be beneficial for CDI^30,31^. Our in silico model also suggested a wave like phenomena where an initial robust Th1/Th17 response is mitigated by a Treg response. We had integrated LANCL2 into our model given its known ability to increase Treg function when activated and decrease Treg activity when inhibited. LANCL2 was specifically incorporated into the model via its ability to activate pyruvate dehydrogenase (PDH) and pyruvate kinase (PK) and activate STAT5 (Fig. 3a). Activation of LANCL2 also results in the translocation of GLUT4 to the plasma membrane; however, CD4+ T cells express mainly GLUT1 (not GLUT4), so this phenomenon was not included in the model. In silico stimulation of LANCL2 via varying doses (10^−7^ to 1 μΜ) of LANCL2 ligands such as ABA during the CDI model resulted in enhanced Treg populations for all days pre and post challenge with the largest changes on Days 3, 4, and 5 where the Treg recovered from 14.1%, 0.1%, and 14.6 to 43%. (Fig. 3b). Conversely, Th17 cells decreased from 37% to <4% for the highest concentrations of ABA. Analysis of CD4+ T cell metabolism on Days 0, 3, 4, 5, 8, and 10 post challenge indicated a dose dependent, increased oxidative phosphorylation and IL-10 production in comparison to untreated control (Fig. 3c). This in silico prediction fit with prior experimental results observed by Leber et al. where activation of LANCL2 resulted in increased Treg cells as well as diminished disease severity in a model of IBD^15^. The increased energy derived from aerobic respiration was due to increased pyruvate dehydrogenase (PDH) activity. Within our CD4+ T cell model, increased PDH activity and fatty acid oxidation leads to higher accumulation of acetyl-coA. Allosteric activation of pyruvate carboxylase via acetyl-CoA causes an increased influx of metabolites into the TCA cycle. Increased TCA metabolites combined with increased acetyl-CoA concentration leads to greater TCA cycle flux, driving higher NADH production. Activated LANCL2 acting on FOXP3 acetylation also alters the gene expression of metabolic enzymes such as ATPase, which has its highest value associated with the Treg cell type. This dual functionality of immune and bioenergetic changes mitigates the differentiation of Th1 and Th17 cells while concurrently enhancing Treg activity within our model.

### Abscisic acid increases Treg function and pushes increased TCA cycle activity

**Fig. 4:**
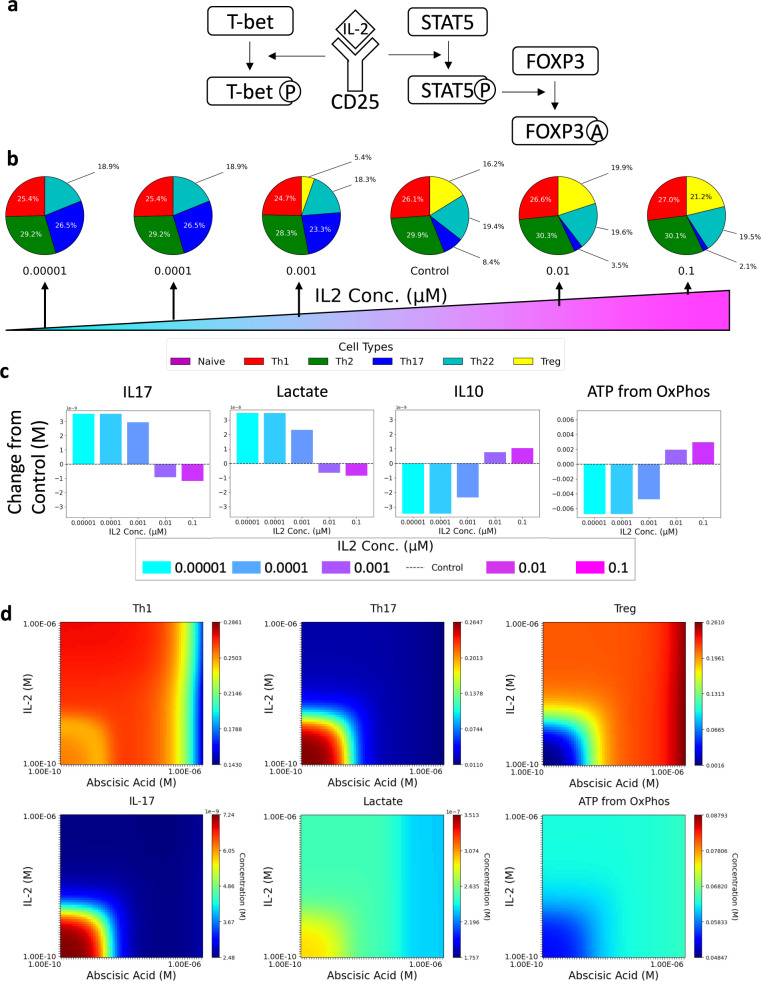
The effect of varying concentrations of IL-2 on CD4+ T cell differentiation using a mixed differentiation cocktail of cytokines. **a** Known connections of IL-2 in the model of CD4+ T cell differentiation. **b** Composition of CD4+ T cells using varying concentrations of IL-2. Baseline signifies starting concentration used in the model **c** Changes in IL-17, lactate, IL-10, and ATP from oxidative phosphorylation using varying concentrations of IL-2 (10^−5^ to 0.1 μΜ). **d** Comparison of varying concentrations of IL-2 (10^−4^ to 1 μΜ) versus ABA (10^−4^ to 1 μΜ) on Th17, Th1, and Treg differentiation as well as IL-17, Lactate, and ATP from oxidative phosphorylation.

An increase in IL-2 has been shown to increase Treg phenotypes as well as broadly enhance proliferation of other CD4+ T cell populations^32^ (Fig. 4a)^33^. resulting in an approximately equal CD4+ T cell proportions. From this baseline, we modulated IL-2 concentration. Increasing IL-2 from 1e−5 μΜ to the control concentration (3.09E−03 μΜ) increased Treg populations from 0.0 to 16.2% (Fig. 4b), while further increasing IL-2 to 0.1 μΜ only resulted in an additional increase to 21.2%. Th17 decreased from 26.5 to 2.1% over the same gradient while other CD4+ T cell subsets were altered marginally. Analysis of metabolism and produced cytokines resulted in a dose-dependent decrease in lactate and IL-17 and a dose-dependent increase in ATP produced from OxPhos and IL-10 (Fig. 4c).

We compared the effects of varying the concentration of IL-2 (10^−7^ to 1 μΜ) to varying the concentration of ABA (10^−7^ to 1 μΜ) (Fig. 4d). Increasing concentrations of ABA and IL-2 both decreased Th17 differentiation and increased Treg differentiation. Increasing ABA and IL-2 both reduced the production of IL-17. ATP produced from OxPhos was increased by similar amounts in response to ABA and IL-2. Lactate production was reduced by both ABA and IL-2, however, higher concentrations of ABA continued to reduce lactate where higher concentrations of IL-2 had no additional effect. Th1 differentiation is affected differently by ABA and IL-2 concentration. As IL-2 concentration increases, it causes a slight increase in Th1 differentiation whereas at higher concentrations of ABA, Th1 differentiation begins to decrease. Additionally, due to pathways associated with proliferation not being included in our model, the IL-2-mediated increase in proliferation cannot be shown. Increased proliferation caused by exogenous IL-2 broadly increase the numbers of both effector and regulatory cells. Our results suggest that IL-2 operates in a narrow concentration margin driving induction of Tregs while LANCL2 activation is effective at broader activity ranges at increasing Treg differentiation while promoting metabolic pathways that are preferred by regulatory cells.

### Exposure of Treg and Th17 cells to extracellular lactate leads to diverging metabolic effects

**Fig. 5:**
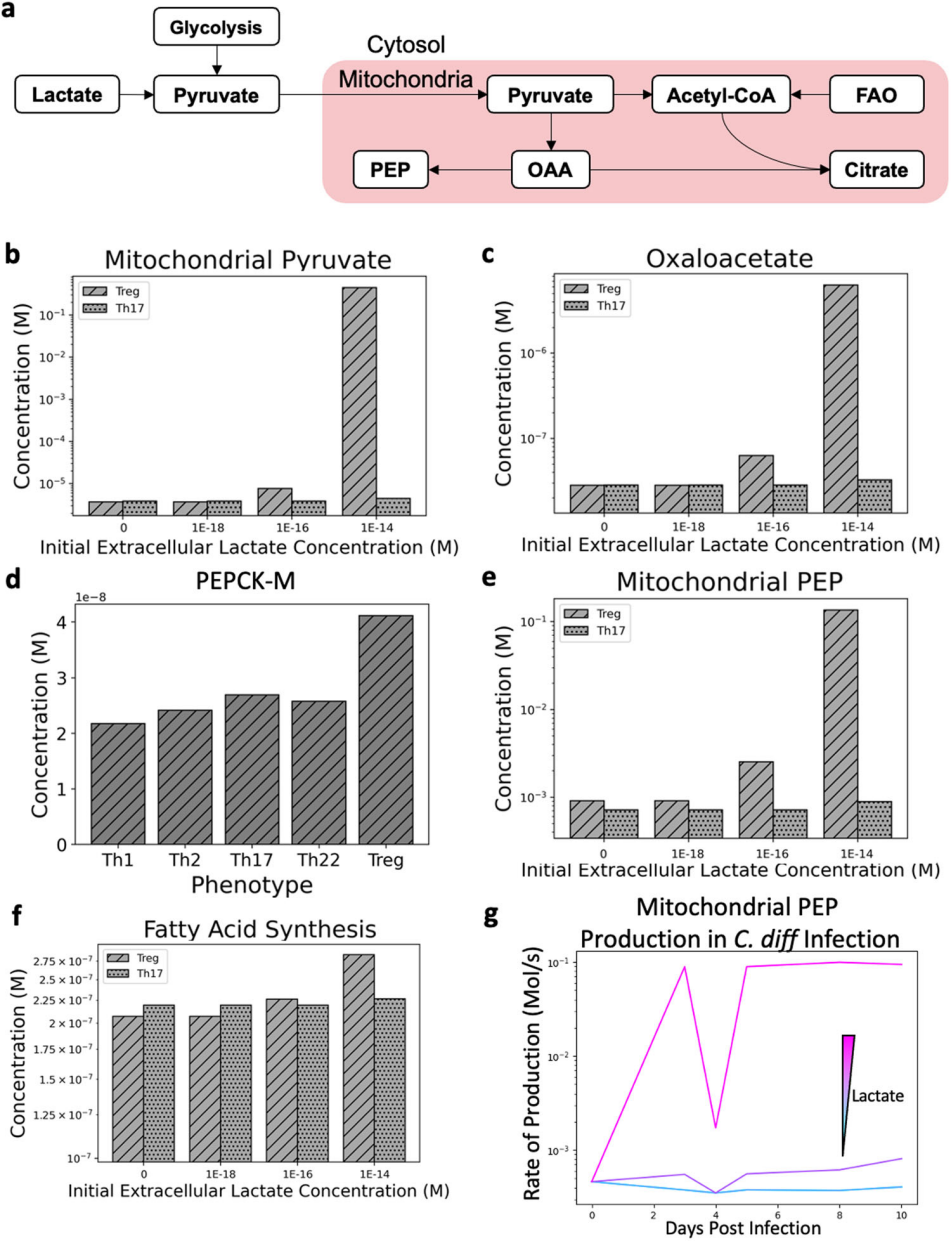
The effect of varying concentrations of extracellular Lactate (0, 10^−9^, 10^−7^, 10^−5^ μM) on Treg, Th17, and a population of T cells responding to *Clostridioides difficile* infection. **a** Cartoon representation of how lactate imported into the cell would be utilized. **b** Increasing mitochondrial pyruvate concentration due to increasing lactate concentrations are more apparent in Treg cell types. **c** Oxaloacetate concentrations within the mitochondria of simulated Treg and Th17 cells. **d** PEPCK-M concentrations for each of the T cell populations. **e** Phosphoenolpyruvate concentrations within the mitochondria of simulated Treg and Th17 cells. **f** Concentration of palmitic acid produced by Treg and Th17 cells under variable extracellular lactate concentrations. **g** Phosphoenolpyruvate concentration within the mitochondria of simulated T cells responding to *Clostridioides difficile* infection under variable lactate concentrations.

Experiments performed by Haas et al. showed that exposing Th17 cells to lactate cause them to have higher production of IL-17 and reduced motility^34^. Conversely, increased lactate uptake in Treg cells makes them more immunosuppressive^35^. With these seemingly opposing results stemming from a metabolic input, we ran an in silico experiment to determine if there were differences in how these two cell types reacted to increased lactate concentrations. For lower concentrations of extracellular lactate, the effect was a slight decrease in efficacy of lactate export from the cell. Increased lactate concentrations led mostly to pyruvate being transported in greater number to the mitochondria (Fig. 5a, b). Due to the increased flux of metabolites, the concentration of oxaloacetate was also increased (Fig. 5c). Due to the expression of the lactate importer being higher in Tregs, as well as the mitochondrial transporter for pyruvate, increasing the concentration of lactate had a greater effect overall on the metabolite concentrations within the mitochondria. This effect is compounded by a further higher expression of PEPCK-M in Treg cell types (Fig. 5d), a rate-limiting enzyme that catalyzes the conversion of oxaloacetate to phosphoenolpyruvate. This reaction is the sole link between the TCA cycle and gluconeogenesis in CD4+ T cells due to the lack of expression of PEPCK in all cell types. This bottleneck in metabolism combined with such a drastic difference in expression causes the amount of PEP produced by Treg cells to be much greater than the amount produced by Th17 cells (Fig. 5e). Another metabolic pathway affected by lactate uptake, fatty acid synthesis, showed the difference between Th17 and Treg production is dampened greatly compared to PEP production levels (Fig. 5f). These results would suggest that PEP exported from the mitochondria, and potentially gluconeogenesis plays an important role in Treg function and differentiation. Higher oxidative phosphorylation capabilities of the Tregs also allows for citrate to progress through the TCA cycle more effectively by maintaining higher levels of NAD+ in the mitochondria. When the *C. difficile* in silico experiment is run multiple times with increasing concentrations of lactate, there is minimal difference to the phenotypes of the T cells, but major differences in the metabolic outcomes associated with Treg cells. Corresponding to the level of Treg within the simulated population, the amount of PEP produced in the mitochondria either increases drastically, or minimally in response to increasing lactate concentrations (Fig. 5g). The immunological discrepancies between Th17 and Treg response to lactate can potentially be explained by their divergent metabolic states, highlighted by the model. Treg cells direct higher amounts of lactate through the mitochondria to gluconeogenesis via PEPCK-M, whereas Th17 cells leverage more non-mitochondrial means of energy generation. Further investigation is required to determine if the increase in gluconeogenesis is responsible for increased immunosuppressive behavior in Tregs.

## Discussion

The homeostatic regulation of immune responses is key to proper resolution of infectious diseases and the management of inflammatory, metabolic, neurodegenerative, and autoimmune diseases. Effector and Treg CD4+ T cell responses work together to provide targeted responses to many different stimuli within the body. While both are necessary for proper immune function, tipping the scales in favor of a Treg response can be beneficial for the protection, resolution, and healing of certain infectious, metabolic, neurodegenerative and autoimmune diseases^36,37^. More specifically, strong effector responses in infectious diseases of the GI tract lead to increased damage of the epithelial layers and loss of barrier function^38,39^. On the other hand, autoimmune diseases are characterized by their excessive and dysregulated effector CD4+ T cell responses^40^. This paper describes the development of a computational and mathematical model of CD4+ T cell differentiation and bioenergetics to elucidate and further characterize the underlying mechanisms by which CD4+ T cells may be pushed towards a Treg phenotype and function. While this model is broadly applicable to any disease in which CD4+ T cells play a major role in the immune response, we focused our initial efforts on the use of the model to describe and predict the effects of potential therapeutic interventions in the context of *C. difficile* infection (CDI). We calibrated our model to experimentally derived cytokine cocktails, identified the baseline immune response to CDI using our model, simulated treatment of CDI with ABA or IL-2 in a comparison study, and identified gluconeogenesis via PEPCK-M as an emerging metabolic pathway implicated in the induction Treg responses.

Our previous computational modeling efforts led to the creation of a CD4+ T cell differentiation model^26^ that could predict the differentiation of four phenotypes (Th1, Th2, Th17, and Treg). The model created by Carbo et al. at the NIMML consisted of multiple cytokine signaling pathways involving the extracellular cytokine, cytokine receptor, various intermediate signaling molecules, and the transcription factors associated with each phenotype^41^. The current work has completely transformed our previous CD4+ T cell model in two major ways: increasing substantially the granularity of the immune signaling and incorporating bioenergetics into the model. The increased granularity of the model allows for the description of an additional phenotype (Th22), while the addition of many bioenergetic pathways provides a metabolic profile for the cell populations and new mechanisms for altering the immune response via new connections from metabolism to the immune signaling pathways^42,43^.

The new model is capable of accurately predicting the phenotype and function of the cell population in addition to its metabolic profile given the cytokine cocktails used in biological experiments for selective differentiation. The validation of the immunological component of the model improves confidence in the model outputs, but the portion of the results associated with the metabolic component of the model are computational estimations of the metabolic profile for each phenotype. The model predicts that effector CD4+ T cells have increased glycolytic activity, with increased lactate export, whereas Treg cells have increased TCA activity and increased ATP production from oxidative phosphorylation. These metabolic profiles match what has been reported previously in experimental metabolic studies of CD4+ T cells^8,44^ and provide scaffolding for additional metabolites to interact with the immune portion of the model. Differentially expressed metabolic genes cause each phenotype’s metabolism to react differently to various perturbations such as altering concentrations of ABA (the natural LANCL2 agonist), IL-2, or extracellular lactate. We have used these differences to compare the therapeutic efficacy of ABA and IL-2 in inducing a Treg response in the context of CDI, as well as to identify pathways that are likely associated with each phenotype.

A key feature of this model is its ability to predict the relative abundance of CD4+ T cell subsets based only on the estimated concentrations of the cytokines within the tissue sample. For instance, the model can take modified RNAseq data to estimate cytokine concentrations and is able to provide predicted phenotypes and immunological functions along with a breakdown of how metabolites are flowing through the cell. This allows for an in-depth estimation of how CD4+ T cells are functioning in an environment without needing to isolate cells from the sample and perform a metabolic analysis on them. We have found that maintaining a comprehensive computational model has allowed for the rapid analysis global system-wide datasets and led to the ability to quickly extract more useful information from the results of biological experiments.

During CDI, broad spectrum antibiotics create a microenvironment that favors successful colonization of by *C. difficile*. Spores ingested during this time survive the gastric acid barrier and enter the colon. *C. difficile* penetrates the mucus layer of the colon, adheres to epithelial cells, and produces toxin A and B, which elicit an inflammatory response by the host^45^. This inflammatory response is associated with disease severity, because as effector cells respond to the invading bacteria, they also cause indiscriminate tissue damage to the epithelial layer of the gut^46^. In a mouse model of CDI, the inflammatory response peaks 3–5 days after inoculation with the spores, followed by a switch to a strong Treg response by days 8–10 which corresponds to the recovery phase of the disease. Our model can accurately reproduce these experimental behaviors and provides an estimate of how each CD4+ T cell phenotype differentiates over time. Experimental studies have shown that reduction of the inflammatory response leads to amelioration of the disease^47^. As such, excessive pathogenic effector CD4+ T cell are indicators of increased colonic pathology and disease severity whereas Treg cells are markers of tissue resolution and disease recovery. Our in silico experiments demonstrated an increase in Th17 cell differentiation during the peak of CDI, and a subsequent decrease going into the recovery stage. Treg cell differentiation was generally inverse to Th17 and was mostly suppressed until the recovery phase. Our model shows Th1, Th2, and Th22 are still able to differentiate due to Th1/Th2 differentiating cytokines being present in substantial concentrations. While the consensus of CDI studies has been that the infection is cleared by day 10, cytokines associated with Th1 and Th2 are still present and potentially contributing to tissue damage. Indeed, our model suggests that the inflammatory response may last for several days past the infection clearance. This lingering inflammation connected to effector responses may disrupt the healthy gut microbiota from recolonizing the gut after CDI and contribute to the recurrence of the infection. As such, increasing the Treg response, even into the recovery phase, may induce better recolonization by healthy microbiota and accelerate recovery.

While the model does show increased inflammation at the end of the infection from Th1/Th2 cell types, disease severity still tracks most strongly with Th17 cells and inversely with Treg cells. Our prior work and that of others has highlighted the importance of LANCL2 as a regulator of immunity and metabolism, increasing Treg differentiation and decreasing Th17 differentiation^15^. From a metabolic perspective, LANCL2 activation results in increased utilization of pyruvate within the mitochondria as opposed to complete glycolysis and lactate production^13,15^. Activation of LANCL2 results in the activation of STAT5 and is a promising avenue for therapeutic development given the high abundance of LANCL2 in Treg cells when compared to other CD4+ T cell subsets^28^. Activation of LANCL2 has been shown to be highly favorable for reducing symptoms of IBD by causing CD4+ T cells to favor regulatory phenotypes^15,16^. Our computational modeling suggests that LANCL2 activation pushes the CD4+ T cell towards a Treg phenotype at the signal transduction, but also pushes for a metabolic environment that mimics that of a Treg cell, including increased aerobic respiration, decreased lactate production, and increased fatty acid utilization.

We compared LANCL2 activation with increasing IL-2 concentration, another potential route for immune signaling and therapeutic intervention. IL-2 is a pleiotropic cytokine capable of inducing T memory and Treg cells at low concentrations and T effector responses at high concentrations^48^. Development of Treg cells occurs via IL-2-JAK1/3-STAT5 STAT5 mediated pathways. How IL-2 drives Th1 differentiation remains unclear, although Ross et al proposed that IL-2 causes the preconditioning of the cells underlying signal transduction network, making CD4+ T-cells more sensitive to Th1 inducing stimuli. Our results suggest that IL-2 activation at lower concentrations induced differentiation into a Treg phenotype, but at elevated concentrations we observed a slight increase in pathogenic Th1 cell populations. The magnitude of change was relatively small compared to changes in cytokines between the days of infections. This smaller change is consistent with the effect of IL-2 on STAT-5 phosphorylation in the general CD4+ T cell population seen by Li et al.^49^. In contrast, the model indicated induction of Tregs via LANCL2 activation induced a more robust activation of Tregs at lower concentrations and did not induce, but rather mitigated Th1 differentiation. Combined with the proliferative effects of IL-2, this further exemplifies the benefit of using LANCL2 activation over IL-2 to induce a sustained Treg response safely and effectively.

The underlying mechanisms by which Tregs and Th17 cells differentiate as well as the metabolic pathways that they depend upon to differentiate are required knowledge to alter the immune response of an organism. With a better understanding of these immunological mechanisms, it is possible to target specific pathways within metabolism to induce Treg subsets. Based on our modeling work, we propose that gluconeogenesis, specifically through the little studied enzyme PEPCK-M, is an important pathway for Treg differentiation and function. Several studies of the uptake of lactate in CD4+ T cells showed that lactate pushes both Th17 cells to become more effector-like and Treg cells to become more immunosuppressive^34,35^. In our model, uptake of lactate increased the concentration of pyruvate entering the mitochondria for both Treg and Th17 cells. However, Treg cells are primed to push additional pyruvate into the TCA cycle due to the increased activity of fatty acid oxidation, which maintains a high concentration of acetyl-CoA, allosterically activating pyruvate carboxylase. Due to their increased expression of PEPCK-M, Tregs can effectively siphon oxaloacetate into the gluconeogenesis pathway by exporting phosphoenolpyruvate from the mitochondria.

This model was engineered to investigate immunological mechanisms controlling CD4+ T cell differentiation and to study the metabolic profiles of such cells. The model can be recalibrated to experimental cytokine concentrations and provides metabolic profiles for CD4+ T cell phenotypes. It captures the known phenomena that effector CD4+ T cells have higher glycolytic activity while Tregs have more oxidative phosphorylation activity. Therefore, while the initial proof-of-concept was done in CDI, the model is a widely applicable tool for the quantitative analysis of the metabolic and immunological mechanisms of CD4+ T cells in the context of a wide range of inflammatory, infectious, metabolic, neurodegenerative, and autoimmune diseases.

## Methods

The CD4+ T cell bioenergetics model was developed as a continuation of the CD4+ T cell differentiation model developed by A. Carbo et al.^26^ We added several species to the model that were discussed as theoretical by Lu et al.^50^, but never actualized in an SBML compliant model, including TNFa, AhR, and IL-22. We also updated the connections within the model to better represent a more up-to-date understanding of immune signaling and CD4+ T cell differentiation. Many metabolic pathways are represented within the model, including glycolysis, gluconeogenesis, the TCA cycle, oxidative phosphorylation, fatty acid oxidation, fatty acid synthesis, and the malate aspartate shuttle. These two portions of the model were developed in parallel, with the now-realized intention of finding connections between the two models to complete the immuno-metabolic collaboration. Connections from metabolism to immunity were identified using literature mining programs that searched for combinations of keywords within the abstracts of articles on PubMed. A complete list of equations can be found in Supplementary File 2, and corresponding parameter values can be found in Supplementary Table 2. The system of differential equations were organized in an Antimony file^51^, which was solved using Pycotools and tellurium python packages^52,53^. For each experiment, a 72 h time-course simulation was performed, and the final values for each model species were output for comparison. The 72 h simulation facilitates the complete differentiation of the cell and allows time for the metabolic trends to become apparent between simulations.

### Immune layer

Immune signaling pathways account for 31 reactions and 75 species within the model. The model takes as inputs external cytokine concentrations (IFNg, IL-2, IL-4, IL-6, IL-9, IL-10, IL-12, IL-21, IL23, TGFb, and TNFa). These cytokines bind to their respective receptors and influence the activation of immune signaling molecules and transcription factors associated with each CD4+ T cell phenotype that has been included in this model (Th1: T-bet, Th2: GATA3, Th17: RORγt, Th22: AhR, Treg: FOXP3). We utilized Hill Equations to create a structure for rate laws that allowed multiple signals to affect the activation or production of immune signaling molecules. The Hill–Langmuir equation can be written as

1 $$\frac{\rm{d}}{{{\rm{d}}}t}\left[ {X_{\rm{prod}}} \right] = c \times \frac{{A^n}}{{\left( {A^n + \left( {k_a} \right)^n} \right)}},$$

for instances of activation by *A*. Conversely, instances of inhibition by *I* can be expressed in a similar format:

2 $$\frac{\rm{d}}{{{\rm{d}}}t}\left[ {X_{\rm{prod}}} \right] = c \times \frac{{\left( {k_i} \right)^n}}{{\left( {I^n + \left( {k_i} \right)^n} \right).}}$$

By combining these equations together, we created a rate law structure that, for a species with three inhibitors and 3 activators, would have the following structure:

3 $$\begin{array}{l}\frac{\rm{d}}{{\rm{d}}t}\left[ {X_{\rm{prod}}} \right] = V_f \times \frac{{\left( {k_{i1}} \right)^{ni1}}}{{I_1^{ni1} + \left( {k_{i1}} \right)^{ni1}}} \times \frac{{\left( {k_{i2}} \right)^{ni2}}}{{I_2^{ni2} + \left( {k_{i2}} \right)^{ni2}}} \\ \qquad \qquad\qquad\, \, \,\times \frac{{\left( {k_{i3}} \right)^{ni3}}}{{I_3^{ni3} + \left( {k_{i3}} \right)^{ni3}}} \times \left( \begin{array}{l}\frac{{c_1 \times A_1^{na1}}}{{A_1^{na1} + \left( {k_{a1}} \right)^{na1}}}\\ + \frac{{c_1 \times A_2^{na2}}}{{A_2^{na2} + \left( {k_{a2}} \right)^{na2}}} + \\ \frac{{c_3 \times A_3^{na3}}}{{A_3^{na3} + \left( {k_{a3}} \right)^{na3}}}\end{array} \right).\end{array}$$

For each signaling molecule and transcription factor, *A* and *I* values were determined by the concentration of the signaling molecule assigned to that variable, and *k*, *c*, and *n* values were estimated using particle swarm, a parameter estimation algorithm. For each phenotype (including Naïve), an input cytokine cocktail was set as the initial condition corresponding to cocktails used in the lab to induce differentiation of each cell type (Supplementary Table 1). The phenotype values were used as the outputs of the model for calibration. The particle swarm algorithm searched the parameter space for parameter sets that minimized the error between expected model outputs and actual model outputs. Each protocol was expected to output phenotype values of 0.85 for the phenotype associated with the protocol, and 0.00 for the phenotype values that were not targeted. A higher weight (5 times) was applied to the error from the protocol-associated phenotype, as to stop the parameter estimation algorithm from minimizing off-target error more than promoting the desired phenotype. A network diagram shows the directionality of interactions between immune signaling molecules (Supplementary File 1). Other connections, such as the effects of LANCL2 activation, had parameters estimated individually to ensure they recapitulated previously published experimental results^15^.

### Metabolism layer

The metabolic layer of the model consists of glycolysis, gluconeogenesis, TCA cycle, fatty acid oxidation, fatty acid synthesis, gluconeogenesis, pentose phosphate pathway, malate-aspartate shuttle, oxidative phosphorylation, and LANCL2 ligand ABA. Glycolysis, the TCA cycle, and oxidative phosphorylation were the initial focus of this model; additional pathways were added and connected when pathway enrichment analyses indicated there would be meaningful change between cell types, and model results indicated that a node required an additional sink or source for the model to be biologically viable. Supplementary File 1 shows the network diagram for metabolism and its connections to the immune layer. Connections from each phenotype to the metabolic enzymes are generalized as to increase legibility of the diagram. Kinetic parameters were obtained from the Sabio-RK Biochemical Reactions Kinetics Database. Most reactions were found using the standard Enzyme Commission number (EC number), followed by ensuring enzyme, substrates, and products matched the expected values. Parameter values that required estimation included those associated with reactions for import/export of molecules. These boundaries of the model required a reduction of enzymatic activity to maintain biological values such as approximate ATP requirements of a CD4+ T cell. Characteristic concentrations for metabolic enzymes were determined using a normalization of single-cell RNAseq data for CD4+ T cells divided by cell type^28^.

### Automated literature mining

Connections between the immune and metabolic layers were identified using the Natural Language ToolKit (NLTK) and Biopython packages in Python^54,55^. Keywords were exhaustively searched in pairs, restricting the keywords to the same sentence. 2321 abstracts were identified as describing potential connections using automated methods. A subset of 130 articles were found to contain meaningful connections between metabolism and immunity after manual curation, which resulted in 2 connections being added into the model from metabolism to immunity (Lactate to STAT3 activation and NADPH to STAT3 activation).

## Supplementary information

Supplementary Materials

## Data Availability

The publicly available single-cell RNAseq study utilized for creating the metabolic portion of the model is available in GEO under the accession number GSE135390.

## Code availability

The model presented here is available for access from the BioModels database under the accession https://www.ebi.ac.uk/biomodels/MODEL2211070001.

## References

[1] Ghose C (2013). Clostridium difficile infection in the twenty-first century. Emerg. Microbes Infect..

[2] Mada, P. K. & Alam, M. U. Clostridioides Difficile. StatPearls. Treasure Island (FL) (2022).

[3] Aktories K, Schwan C, Jank T (2017). Clostridium difficile Toxin Biology. Annu. Rev. Microbiol..

[4] Cowardin CA, Petri WA (2014). Host recognition of Clostridium difficile and the innate immune response. Anaerobe.

[5] Nakagawa T (2016). Endogenous IL-17 as a factor determining the severity of Clostridium difficile infection in mice. J. Med. Microbiol..

[6] Leber A (2015). Systems modeling of interactions between mucosal immunity and the gut microbiome during clostridium difficile infection. PLoS One.

[7] Round JL, Mazmanian SK (2010). Inducible Foxp3+ regulatory T-cell development by a commensal bacterium of the intestinal microbiota. Proc. Natl Acad. Sci. USA.

[8] Michalek RD (2011). Cutting edge: distinct glycolytic and lipid oxidative metabolic programs are essential for effector and regulatory CD4+ T cell subsets. J. Immunol..

[9] Shin B (2020). Mitochondrial oxidative phosphorylation regulates the fate decision between pathogenic Th17 and regulatory T cells. Cell Rep..

[10] Gerriets VA (2015). Metabolic programming and PDHK1 control CD4+ T cell subsets and inflammation. J. Clin. Invest..

[11] O’Neill LA, Kishton RJ, Rathmell J (2016). A guide to immunometabolism for immunologists. Nat. Rev. Immunol..

[12] Atkinson, F. S. et al. Abscisic acid standardized fig (Ficus carica) extracts ameliorate postprandial glycemic and insulinemic responses in healthy adults. *Nutrients*10.3390/nu11081757 (2019). 10.3390/nu11081757 PMC6722713 31370154

[13] Leber A (2020). Abscisic acid enriched fig extract promotes insulin sensitivity by decreasing systemic inflammation and activating LANCL2 in skeletal muscle. Sci. Rep..

[14] He C (2017). LanCL proteins are not involved in lanthionine synthesis in mammals. Sci. Rep..

[15] Leber A, Hontecillas R, Zoccoli-Rodriguez V, Bassaganya-Riera J (2018). Activation of LANCL2 by BT-11 ameliorates IBD by supporting regulatory T cell stability through immunometabolic mechanisms. Inflamm. Bowel Dis..

[16] Leber A, Hontecillas R, Zoccoli-Rodriguez V, Chauhan J, Bassaganya-Riera J (2019). Oral treatment with BT-11 ameliorates inflammatory bowel disease by enhancing regulatory T cell responses in the gut. J. Immunol..

[17] Tubau-Juni N (2021). First-in-class topical therapeutic omilancor ameliorates disease severity and inflammation through activation of LANCL2 pathway in psoriasis. Sci. Rep..

[18] Tyson JJ, Chen KC, Novak B (2003). Sniffers, buzzers, toggles, and blinkers: dynamics of regulatory and signaling pathways in the cell. Curr. Opin. Cell Biol..

[19] Franke, R., Theis, F. J. & Klamt, S. From binary to multivalued to continuous models: the lac operon as a case study. *J. Integr. Bioinform.*10.2390/biecoll-jib-2010-151 (2010). 10.2390/biecoll-jib-2010-151 21200084

[20] Klipp E, Liebermeister W (2006). Mathematical modeling of intracellular signaling pathways. BMC Neurosci..

[21] Leber A (2016). Modeling the role of lanthionine synthetase C-like 2 (LANCL2) in the modulation of immune responses to helicobacter pylori infection. PLoS One.

[22] Verma M (2017). Modeling the mechanisms by which HIV-associated immunosuppression influences HPV persistence at the oral mucosa. PLoS One.

[23] Verma, M. et al. High-resolution computational modeling of immune responses in the gut. *Gigascience*10.1093/gigascience/giz062 (2019). 10.1093/gigascience/giz062 PMC6559340 31185494

[24] Heiske M, Letellier T, Klipp E (2017). Comprehensive mathematical model of oxidative phosphorylation valid for physiological and pathological conditions. FEBS J..

[25] Nazaret C, Heiske M, Thurley K, Mazat JP (2009). Mitochondrial energetic metabolism: a simplified model of TCA cycle with ATP production. J. Theor. Biol..

[26] Carbo A (2013). Systems modeling of molecular mechanisms controlling cytokine-driven CD4+ T cell differentiation and phenotype plasticity. PLoS Comput. Biol..

[27] Wittig U (2012). SABIO-RK–database for biochemical reaction kinetics. Nucleic Acids Res..

[28] Hollbacher B (2020). Transcriptomic profiling of human effector and regulatory T cell subsets identifies predictive population signatures. Immunohorizons.

[29] Eberhart, J. K. A. R. Particle swarm optimization. In *Proceedings of ICNN'95 - International Conference on Neural Networks*10.1109/ICNN.1995.488968 (1995).

[30] Saleh MM (2019). Colitis-induced Th17 cells increase the risk for severe subsequent clostridium difficile infection. Cell Host Microbe.

[31] Yacyshyn MB (2014). Clostridium difficile recurrence is characterized by pro-inflammatory peripheral blood mononuclear cell (PBMC) phenotype. J. Med. Microbiol..

[32] Bachmann MF, Oxenius A (2007). Interleukin 2: from immunostimulation to immunoregulation and back again. EMBO Rep..

[33] Rodriguez C (2020). Microbiota insights in clostridium difficile infection and inflammatory bowel disease. Gut Microbes.

[34] Haas R (2015). Lactate regulates metabolic and pro-inflammatory circuits in control of T cell migration and effector functions. PLoS Biol..

[35] Multhoff G, Vaupel P (2021). Lactate-avid regulatory T cells: metabolic plasticity controls immunosuppression in tumour microenvironment. Signal Transduct. Target Ther..

[36] Yamada A (2016). Role of regulatory T cell in the pathogenesis of inflammatory bowel disease. World J. Gastroenterol..

[37] Taams LS (2006). Regulatory T cells in human disease and their potential for therapeutic manipulation. Immunology.

[38] Nusrat A (2001). Clostridium difficile toxins disrupt epithelial barrier function by altering membrane microdomain localization of tight junction proteins. Infect. Immun..

[39] Slomiany BL, Slomiany A (1992). Mechanism of Helicobacter pylori pathogenesis: focus on mucus. J. Clin. Gastroenterol..

[40] Wallace KL, Zheng LB, Kanazawa Y, Shih DQ (2014). Immunopathology of inflammatory bowel disease. World J. Gastroenterol..

[41] Carbo A (2014). Computational modeling of heterogeneity and function of CD4+ T cells. Front. Cell Dev. Biol..

[42] Xie Q (2020). A lactate-induced Snail/STAT3 pathway drives GPR81 expression in lung cancer cells. Biochim. Biophys. Acta Mol. Basis Dis..

[43] Jung SN (2015). Sugiol inhibits STAT3 activity via regulation of transketolase and ROS-mediated ERK activation in DU145 prostate carcinoma cells. Biochem. Pharm..

[44] Fox CJ, Hammerman PS, Thompson CB (2005). Fuel feeds function: energy metabolism and the T-cell response. Nat. Rev. Immunol..

[45] Savidge TC (2003). Clostridium difficile toxin B is an inflammatory enterotoxin in human intestine. Gastroenterology.

[46] Pene J (2008). Chronically inflamed human tissues are infiltrated by highly differentiated Th17 lymphocytes. J. Immunol..

[47] Wang S, Deng W, Li F, Chen YE, Wang PU (2021). Blockade of T helper 17 cell function ameliorates recurrent Clostridioides difficile infection in mice. Acta Biochim. Biophys. Sin..

[48] Ross SH, Cantrell DA (2018). Signaling and function of interleukin-2 in T lymphocytes. Annu. Rev. Immunol..

[49] Li C, Park JH (2020). Assessing IL-2-induced STAT5 phosphorylation in fixed, permeabilized Foxp3(+) Treg cells by multiparameter flow. Cytom. STAR Protoc..

[50] Lu P (2015). Supervised learning methods in modeling of CD4+ T cell heterogeneity. BioData Min..

[51] Smith LP, Bergmann FT, Chandran D, Sauro HM (2009). Antimony: a modular model definition language. Bioinformatics.

[52] Welsh CM (2018). PyCoTools: a Python toolbox for COPASI. Bioinformatics.

[53] Choi K (2018). Tellurium: An extensible python-based modeling environment for systems and synthetic biology. Biosystems.

[54] Bird, S., Klein, E. & Loper, E. Natural language processing with Python: analyzing text with the natural language toolkit. “O’Reilly Media, Inc.” (2009).

[55] Cock PJ (2009). Biopython: freely available Python tools for computational molecular biology and bioinformatics. Bioinformatics.

