# Supplementary material for: Computational modeling of complex bioenergetic mechanisms that modulate CD4+ T cell effector and regulatory functions
Source: NPJ Syst Biol Appl. 2022 Nov 22;8:45. doi: 10.1038/s41540-022-00263-4 (PMC9684137; doi:10.1038/s41540-022-00263-4)
Supplement: Supplementary file 1 — Supplementary Materials [file 41540_2022_263_MOESM1_ESM.pdf]

Supp File 1. Network diagram of CD4+ T-cell differentiation and metabolic model.

Supp File 2. List of all equations used in model.

Supp figure 1. Results of pairwise searching from PubMed abstracts using metabolites, metabolic enzymes, and immune molecules from the model.

Supp Figure 2. Results of pairwise search when co-occurring terms were within the same sentence, with a focus on matching immune and metabolic terms together.

Supp Table 1. Input cytokine concentrations. Includes Phenotype Differentiation Protocols in vitro and expression of cytokines during *in vivo C. difficile* infection. Values of zero are substituted with 10-13 in order to facilitate biologically relevant calibration results. This value was chosen due to it being two orders of magnitude lower than the lowest normalized enzyme value within the RNAseq dataset used for the CD4+ T-cells.

Supp Table 2. Parameter values for each reaction. Abbreviations for the pathway associated with each reaction precede the reaction name. They are as follows: allosteric (AL), fatty acid oxidation (FAO), fatty acid synthesis (FAS), gluconeogenesis (GLU), glycolysis (GLY), immunity (IM), malate-aspartate shuttle (MAS), oxidative phosphorylation (OXPHOS), pentose phosphate pathway (PPP), tricarboxylic acid cycle (TCA)

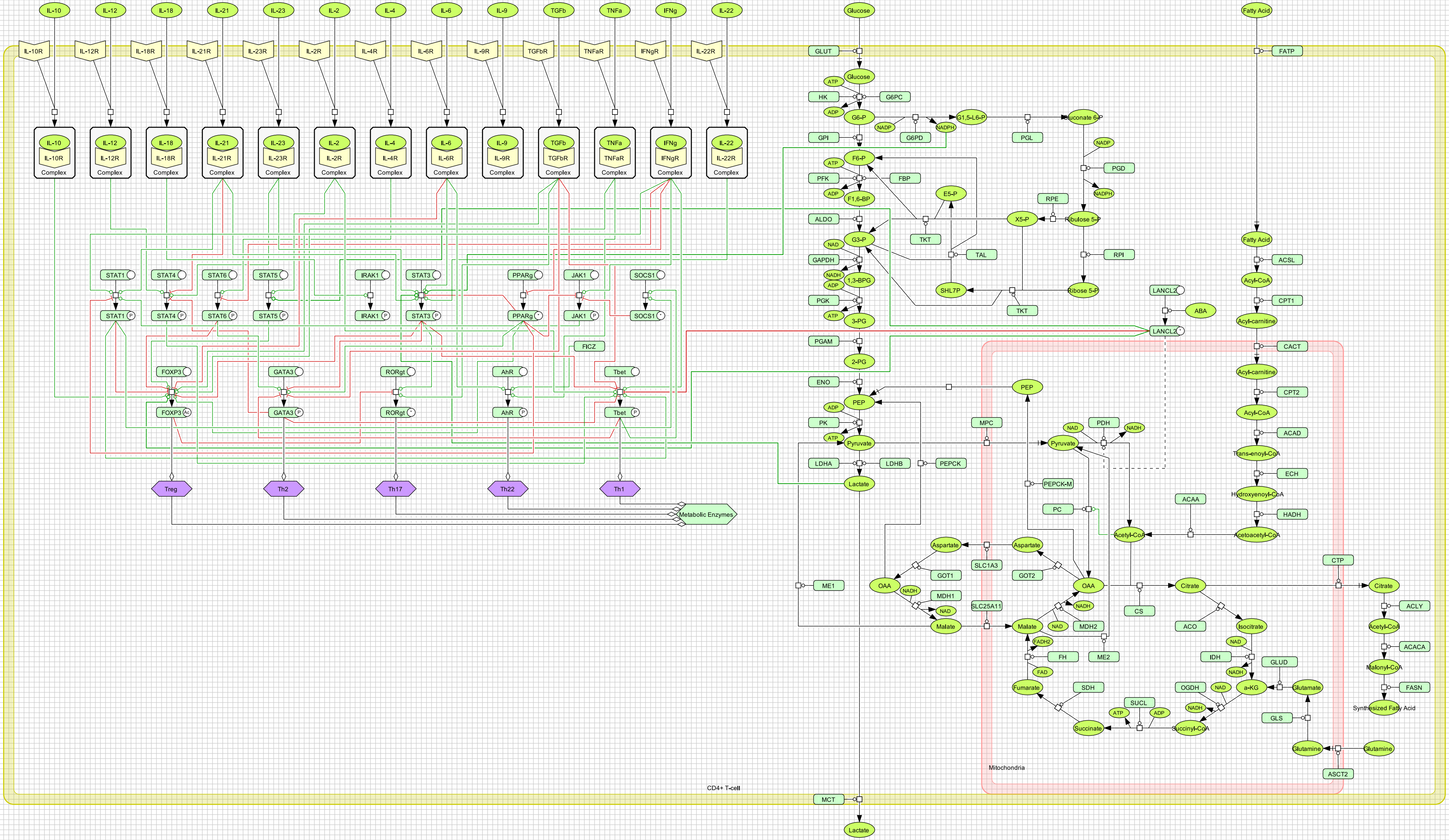

IL-10
IL-12
IL-18
IL-21
IL-23
IL-2
IL-4
IL-6
IL-9
TGFb
TNFa
IFNg
IL-22
IL-10R
IL-12R
IL-18R
IL-21R
IL-23R
IL-2R
IL-4R
IL-6R
IL-9R
TGFbR
TNFaR
IFNgR
IL-22R
Complex
STAT1
STAT4
STAT6
STAT5
IRAK1
STAT3
PPARg
JAK1
SOCS1
FICZ
FOXP3
GATA3
RORgt
AhR
Tbet
Treg
Th2
Th17
Th22
Th1
Metabolic Enzymes
Glucose
GLUT
ATP
ADP
HK
G6PC
G6-P
NADP
NADPH
G6PD
31,5-L6-P
PGL
Gluconate 6-P
PGD
GPI
F6-P
PFK
FBP
F1,6-BP
E5-P
RPE
X5-P
Ribulose 5-P
ALDO
TKT
G3-P
TAL
RPI
NAD
NADH
GAPDH
1,3-BPG
SHL7P
Ribose 5-P
PGK
3-PG
PGAM
2-PG
ENO
PEP
PK
Pyruvate
MPC
PDH
LDHA
LDHB
PEPCK
PEPCK-M
Lactate
PC
Acetyl-CoA
ACAA
Aspartate
SLC1A3
GOT1
GOT2
OAA
MDH1
MDH2
ME1
ME2
SLC25A11
Malate
FADH2
FAD
FH
Fumarate
SDH
Succinate
SUCL
Succinyl-CoA
OGDH
a-KG
IDH
Isocitrate
ACO
Citrate
CS
GLUD
Glutamate
GLS
Glutamine
ASCT2
CTP
ACLY
ACACA
Malonyl-CoA
FASN
Synthesized Fatty Acid
Fatty Acid
FATP
ACSL
Acyl-CoA
CPT1
Acyl-carnitine
CACT
CPT2
ACAD
Trans-enoyl-CoA
ECH
Hydroxyenoyl-CoA
HADH
Acetoacetyl-CoA
LANCL2
ABA
Mitochondria
CD4+ T-cell
MCT

$$\frac{d\left([IL22]\cdot V_{ThCell}\right)}{dt} = -V_{ThCell}\cdot\left(k1_{\text{("(IM) IL22 receptor binding")}}\cdot[IL22]\cdot[IL22R]-k2_{\text{("(IM) IL22 receptor binding")}}\cdot[\text{"IL22-IL22R"}]\right)$$

$$\frac{d\left([\text{"IL12-IL12R"}]\cdot V_{ThCell}\right)}{dt} = +V_{ThCell}\cdot\left(k1_{\text{("(IM) IL12 receptor binding")}}\cdot[IL12\_pool]\cdot[IL12R]-k2_{\text{("(IM) IL12 receptor binding")}}\cdot[\text{"IL12-IL12R"}]\right)$$

$$\frac{d\left([\text{"IL18-IL18R"}]\cdot V_{ThCell}\right)}{dt} = +V_{ThCell}\cdot\left(k1_{\text{("(IM) IL18 receptor binding")}}\cdot[IL18\_pool]\cdot[IL18R]-k2_{\text{("(IM) IL18 receptor binding")}}\cdot[\text{"IL18-IL18R"}]\right)$$

$$\frac{d\left([\text{"IL23-IL23R"}]\cdot V_{ThCell}\right)}{dt} = +V_{ThCell}\cdot\left(k1_{\text{("(IM) IL23 receptor binding")}}\cdot[IL23\_pool]\cdot[IL23R]-k2_{\text{("(IM) IL23 receptor binding")}}\cdot[\text{"IL23-IL23R"}]\right)$$

$$\frac{d\left([\text{"L-PPARg"}]\cdot V_{ThCell}\right)}{dt} = +V_{ThCell}\cdot\left(Vf_{\text{("(IM) PPARg activation")}}\cdot[PPARg]\cdot\frac{ki1_{\text{("(IM) PPARg activation")}}^{n1_{\text{("(IM) PPARg activation")}}}}{[\text{"IFNg-IFNgR"}]^{n1_{\text{("(IM) PPARg activation")}}}+ki1_{\text{("(IM) PPARg activation")}}^{n1_{\text{("(IM) PPARg activation")}}}}\cdot 1-Vr_{\text{("(IM) PPARg activation")}}\cdot[\text{"L-PPARg"}]\right)$$

$$\frac{d\left([\text{"STAT3-P"}]\cdot V_{ThCell}\right)}{dt} = +V_{ThCell}\cdot\left(Vf_{\text{("(IM) STAT3 activation")}}\cdot[STAT3]\cdot\frac{ki1_{\text{("(IM) STAT3 activation")}}^{n1_{\text{("(IM) STAT3 activation")}}}}{[\text{"L-PPARg"}]^{n1_{\text{("(IM) STAT3 activation")}}}+ki1_{\text{("(IM) STAT3 activation")}}^{n1_{\text{("(IM) STAT3 activation")}}}}\cdot\left(\frac{c1_{\text{("(IM) STAT3 activation")}}\cdot[\text{"IL21-IL21R"}]^{n2_{\text{("(IM) STAT3 activation")}}}}{[\text{"IL21-IL21R"}]^{n2_{\text{("(IM) STAT3 activation")}}}+ka1_{\text{("(IM) STAT3 activation")}}^{n2_{\text{("(IM) STAT3 activation")}}}}+\frac{c2_{\text{("(IM) STAT3 activation")}}\cdot[\text{"IL23-IL23R"}]^{n3_{\text{("(IM) STAT3 activation")}}}}{[\text{"IL23-IL23R"}]^{n3_{\text{("(IM) STAT3 activation")}}}+ka2_{\text{("(IM) STAT3 activation")}}^{n3_{\text{("(IM) STAT3 activation")}}}}+\frac{c3_{\text{("(IM) STAT3 activation")}}\cdot[Lactate\{Cytosol\}]^{n4_{\text{("(IM) STAT3 activation")}}}}{[Lactate\{Cytosol\}]^{n4_{\text{("(IM) STAT3 activation")}}}+ka3_{\text{("(IM) STAT3 activation")}}^{n4_{\text{("(IM) STAT3 activation")}}}}+\frac{c4_{\text{("(IM) STAT3 activation")}}\cdot[\text{"IL6-IL6R"}]^{n5_{\text{("(IM) STAT3 activation")}}}}{[\text{"IL6-IL6R"}]^{n5_{\text{("(IM) STAT3 activation")}}}+ka4_{\text{("(IM) STAT3 activation")}}^{n5_{\text{("(IM) STAT3 activation")}}}}+\frac{c5_{\text{("(IM) STAT3 activation")}}\cdot[\text{"IL22-IL22R"}]^{n6_{\text{("(IM) STAT3 activation")}}}}{[\text{"IL22-IL22R"}]^{n6_{\text{("(IM) STAT3 activation")}}}+ka5_{\text{("(IM) STAT3 activation")}}^{n6_{\text{("(IM) STAT3 activation")}}}}+\frac{c6_{\text{("(IM) STAT3 activation")}}\cdot[NADPH\_tot]^{n7_{\text{("(IM) STAT3 activation")}}}}{[NADPH\_tot]^{n7_{\text{("(IM) STAT3 activation")}}}+ka6_{\text{("(IM) STAT3 activation")}}^{n7_{\text{("(IM) STAT3 activation")}}}}\right)-Vr_{\text{("(IM) STAT3 activation")}}\cdot[\text{"STAT3-P"}]\right)$$

$$\frac{d\left([\text{"STAT4-P"}]\cdot V_{ThCell}\right)}{dt} = +V_{ThCell}\cdot\left(Vf_{\text{("(IM) STAT4 activation")}}\cdot[STAT4]\cdot\frac{ki1_{\text{("(IM) STAT4 activation")}}^{n1_{\text{("(IM) STAT4 activation")}}}}{[\text{"IL21-IL21R"}]^{n1_{\text{("(IM) STAT4 activation")}}}+ki1_{\text{("(IM) STAT4 activation")}}^{n1_{\text{("(IM) STAT4 activation")}}}}\cdot\frac{ki2_{\text{("(IM) STAT4 activation")}}^{n2_{\text{("(IM) STAT4 activation")}}}}{[\text{"GATA3-P"}]^{n2_{\text{("(IM) STAT4 activation")}}}+ki2_{\text{("(IM) STAT4 activation")}}^{n2_{\text{("(IM) STAT4 activation")}}}}\cdot^{\text{Type something...}}\left(\frac{c1_{\text{("(IM) STAT4 activation")}}\cdot[\text{"IL12-IL12R"}]^{n3_{\text{("(IM) STAT4 activation")}}}}{[\text{"IL12-IL12R"}]^{n3_{\text{("(IM) STAT4 activation")}}}+ka1_{\text{("(IM) STAT4 activation")}}^{n3_{\text{("(IM) STAT4 activation")}}}}+\frac{c2_{\text{("(IM) STAT4 activation")}}\cdot[\text{"IL23-IL23R"}]^{n4_{\text{("(IM) STAT4 activation")}}}}{[\text{"IL23-IL23R"}]^{n4_{\text{("(IM) STAT4 activation")}}}+ka2_{\text{("(IM) STAT4 activation")}}^{n4_{\text{("(IM) STAT4 activation")}}}}\right)-Vr_{\text{("(IM) STAT4 activation")}}\cdot[\text{"STAT4-P"}]\right)$$

$$\frac{d\left([\text{"Tbet-P"}]\cdot V_{ThCell}\right)}{dt} = +V_{ThCell}\cdot\left(Vf_{\text{("(IM) Tbet activation")}}\cdot[Tbet]\cdot\frac{ki1_{\text{("(IM) Tbet activation")}}^{n1_{\text{("(IM) Tbet activation")}}}}{[\text{"TGFb-TGFbR"}]^{n1_{\text{("(IM) Tbet activation")}}}+ki1_{\text{("(IM) Tbet activation")}}^{n1_{\text{("(IM) Tbet activation")}}}}\cdot\frac{ki2_{\text{("(IM) Tbet activation")}}^{n2_{\text{("(IM) Tbet activation")}}}}{[\text{"STAT3-P"}]^{n2_{\text{("(IM) Tbet activation")}}}+ki2_{\text{("(IM) Tbet activation")}}^{n2_{\text{("(IM) Tbet activation")}}}}\cdot\frac{ki3_{\text{("(IM) Tbet activation")}}^{n3_{\text{("(IM) Tbet activation")}}}}{[\text{"GATA3-P"}]^{n3_{\text{("(IM) Tbet activation")}}}+ki3_{\text{("(IM) Tbet activation")}}^{n3_{\text{("(IM) Tbet activation")}}}}\cdot\frac{ki4_{\text{("(IM) Tbet activation")}}^{n4_{\text{("(IM) Tbet activation")}}}}{[\text{"LANCL2-ABA"}]^{n4_{\text{("(IM) Tbet activation")}}}+ki4_{\text{("(IM) Tbet activation")}}^{n4_{\text{("(IM) Tbet activation")}}}}\cdot\left(\frac{c1_{\text{("(IM) Tbet activation")}}\cdot[\text{"STAT4-P"}]^{n5_{\text{("(IM) Tbet activation")}}}}{[\text{"STAT4-P"}]^{n5_{\text{("(IM) Tbet activation")}}}+ka1_{\text{("(IM) Tbet activation")}}^{n5_{\text{("(IM) Tbet activation")}}}}+\frac{c2_{\text{("(IM) Tbet activation")}}\cdot[\text{"STAT1-P"}]^{n6_{\text{("(IM) Tbet activation")}}}}{[\text{"STAT1-P"}]^{n6_{\text{("(IM) Tbet activation")}}}+ka2_{\text{("(IM) Tbet activation")}}^{n6_{\text{("(IM) Tbet activation")}}}}+\frac{c3_{\text{("(IM) Tbet activation")}}\cdot[\text{"IFNg-IFNgR"}]^{n7_{\text{("(IM) Tbet activation")}}}}{[\text{"IFNg-IFNgR"}]^{n7_{\text{("(IM) Tbet activation")}}}+ka3_{\text{("(IM) Tbet activation")}}^{n7_{\text{("(IM) Tbet activation")}}}}+\frac{c4_{\text{("(IM) Tbet activation")}}\cdot[\text{"IL2-IL2R"}]^{n8_{\text{("(IM) Tbet activation")}}}}{[\text{"IL2-IL2R"}]^{n8_{\text{("(IM) Tbet activation")}}}+ka4_{\text{("(IM) Tbet activation")}}^{n8_{\text{("(IM) Tbet activation")}}}}\right)-Vr_{\text{("(IM) Tbet activation")}}\cdot[\text{"Tbet-P"}]\right)$$

$$\frac{d\left([\text{"IFNg-IFNgR"}]\cdot V_{ThCell}\right)}{dt} = +V_{ThCell}\cdot\left(k1_{\text{("(IM) IFNg receptor binding")}}\cdot[IFNg\_pool]\cdot[IFNgR]-k2_{\text{("(IM) IFNg receptor binding")}}\cdot[\text{"IFNg-IFNgR"}]\right)$$

$$\frac{d\left([\text{IFNgR}]\cdot V_{\text{ThCell}}\right)}{dt} = -V_{\text{ThCell}}\cdot\left(k1_{\text{("(IM) IFNg receptor binding")}}\cdot[\text{IFNg\_pool}]\cdot[\text{IFNgR}] - k2_{\text{("(IM) IFNg receptor binding")}}\cdot[\text{"IFNg-IFNgR"}]\right)$$

$$\frac{d\left([\text{"SOCS1-JAKs"}]\cdot V_{\text{ThCell}}\right)}{dt} = +V_{\text{ThCell}}\cdot\left(Vf_{\text{("(IM) SOCS1 activation")}}\cdot[\text{SOCS1}]\cdot\left(\frac{c1_{\text{("(IM) SOCS1 activation")}}\cdot[\text{"STAT1-P"}]^{n1_{\text{("(IM) SOCS1 activation")}}}}{[\text{"STAT1-P"}]^{n1_{\text{("(IM) SOCS1 activation")}}} + ka1_{\text{("(IM) SOCS1 activation")}}^{n1_{\text{("(IM) SOCS1 activation")}}}} + \frac{c2_{\text{("(IM) SOCS1 activation")}}\cdot[\text{"Tbet-P"}]^{n2_{\text{("(IM) SOCS1 activation")}}}}{[\text{"Tbet-P"}]^{n2_{\text{("(IM) SOCS1 activation")}}} + ka2_{\text{("(IM) SOCS1 activation")}}^{n2_{\text{("(IM) SOCS1 activation")}}}}\right) - Vr_{\text{("(IM) SOCS1 activation")}}\cdot[\text{"SOCS1-JAKs"}]\right)$$

$$\frac{d\left([\text{"AhR-P"}]\cdot V_{\text{ThCell}}\right)}{dt} = +V_{\text{ThCell}}\cdot\left(Vf_{\text{("(IM) AhR activation")}}\cdot[\text{AhR}]\cdot\left(\frac{c1_{\text{("(IM) AhR activation")}}\cdot[\text{"TNFa-TNFaR"}]^{n1_{\text{("(IM) AhR activation")}}}}{[\text{"TNFa-TNFaR"}]^{n1_{\text{("(IM) AhR activation")}}} + ka1_{\text{("(IM) AhR activation")}}^{n1_{\text{("(IM) AhR activation")}}}} + \frac{c2_{\text{("(IM) AhR activation")}}\cdot[\text{"IL6-IL6R"}]^{n2_{\text{("(IM) AhR activation")}}}}{[\text{"IL6-IL6R"}]^{n2_{\text{("(IM) AhR activation")}}} + ka2_{\text{("(IM) AhR activation")}}^{n2_{\text{("(IM) AhR activation")}}}} + \frac{c3_{\text{("(IM) AhR activation")}}\cdot[\text{FICZ}]^{n3_{\text{("(IM) AhR activation")}}}}{[\text{FICZ}]^{n3_{\text{("(IM) AhR activation")}}} + ka3_{\text{("(IM) AhR activation")}}^{n3_{\text{("(IM) AhR activation")}}}}\right) - Vr_{\text{("(IM) AhR activation")}}\cdot[\text{"AhR-P"}]\right)$$

$$\frac{d\left([\text{"GATA3-P"}]\cdot V_{\text{ThCell}}\right)}{dt} = +V_{\text{ThCell}}\cdot\left(Vf_{\text{("(IM) GATA3 activation")}}\cdot[\text{GATA3}]\cdot\frac{ki1_{\text{("(IM) GATA3 activation")}}^{n1_{\text{("(IM) GATA3 activation")}}}}{[\text{"Tbet-P"}]^{n1_{\text{("(IM) GATA3 activation")}}} + ki1_{\text{("(IM) GATA3 activation")}}^{n1_{\text{("(IM) GATA3 activation")}}}}\cdot\frac{ki2_{\text{("(IM) GATA3 activation")}}^{n2_{\text{("(IM) GATA3 activation")}}}}{[\text{"STAT3-P"}]^{n2_{\text{("(IM) GATA3 activation")}}} + ki2_{\text{("(IM) GATA3 activation")}}^{n2_{\text{("(IM) GATA3 activation")}}}}\cdot\frac{ki3_{\text{("(IM) GATA3 activation")}}^{n3_{\text{("(IM) GATA3 activation")}}}}{[\text{"TGFb-TGFbR"}]^{n3_{\text{("(IM) GATA3 activation")}}} + ki3_{\text{("(IM) GATA3 activation")}}^{n3_{\text{("(IM) GATA3 activation")}}}}\cdot\left(\frac{c1_{\text{("(IM) GATA3 activation")}}\cdot[\text{"STAT6-P"}]^{n4_{\text{("(IM) GATA3 activation")}}}}{[\text{"STAT6-P"}]^{n4_{\text{("(IM) GATA3 activation")}}} + ka1_{\text{("(IM) GATA3 activation")}}^{n4_{\text{("(IM) GATA3 activation")}}}} + \frac{c2_{\text{("(IM) GATA3 activation")}}\cdot[\text{"L-PPARg"}]^{n5_{\text{("(IM) GATA3 activation")}}}}{[\text{"L-PPARg"}]^{n5_{\text{("(IM) GATA3 activation")}}} + ka2_{\text{("(IM) GATA3 activation")}}^{n5_{\text{("(IM) GATA3 activation")}}}}\right) - Vr_{\text{("(IM) GATA3 activation")}}\cdot[\text{"GATA3-P"}]\right)$$

$$\frac{d\left([\text{"IL21-IL21R"}]\cdot V_{\text{ThCell}}\right)}{dt} = +V_{\text{ThCell}}\cdot\left(k1_{\text{("(IM) IL21 receptor binding")}}\cdot[\text{IL21\_pool}]\cdot[\text{IL21R}] - k2_{\text{("(IM) IL21 receptor binding")}}\cdot[\text{"IL21-IL21R"}]\right)$$

$$\frac{d\left([\text{"STAT5-P"}]\cdot V_{\text{ThCell}}\right)}{dt} = +V_{\text{ThCell}}\cdot\left(Vf_{\text{("(IM) STAT5 activation")}}\cdot[\text{STAT5}]\cdot\left(\frac{c1_{\text{("(IM) STAT5 activation")}}\cdot[\text{"IL2-IL2R"}]^{n1_{\text{("(IM) STAT5 activation")}}}}{[\text{"IL2-IL2R"}]^{n1_{\text{("(IM) STAT5 activation")}}} + ka1_{\text{("(IM) STAT5 activation")}}^{n1_{\text{("(IM) STAT5 activation")}}}} + \frac{c2_{\text{("(IM) STAT5 activation")}}\cdot[\text{"LANCL2-ABA"}]^{n2_{\text{("(IM) STAT5 activation")}}}}{[\text{"LANCL2-ABA"}]^{n2_{\text{("(IM) STAT5 activation")}}} + ka_{\text{("(IM) STAT5 activation")}}^{n2_{\text{("(IM) STAT5 activation")}}}}\right) - Vr_{\text{("(IM) STAT5 activation")}}\cdot[\text{"STAT5-P"}]\right)$$

$$\frac{d\left([\text{"IL10-IL10R"}]\cdot V_{\text{ThCell}}\right)}{dt} = +V_{\text{ThCell}}\cdot\left(k1_{\text{("(IM) IL10 receptor binding")}}\cdot[\text{IL10\_pool}]\cdot[\text{IL10R}] - k2_{\text{("(IM) IL10 receptor binding")}}\cdot[\text{"IL10-IL10R"}]\right)$$

$$\frac{d\left([\text{IL10R}]\cdot V_{\text{ThCell}}\right)}{dt} = -V_{\text{ThCell}}\cdot\left(k1_{\text{("(IM) IL10 receptor binding")}}\cdot[\text{IL10\_pool}]\cdot[\text{IL10R}] - k2_{\text{("(IM) IL10 receptor binding")}}\cdot[\text{"IL10-IL10R"}]\right)$$

$$\frac{d\left([\text{IL12R}]\cdot V_{\text{ThCell}}\right)}{dt} = -V_{\text{ThCell}}\cdot\left(k1_{\text{("(IM) IL12 receptor binding")}}\cdot[\text{IL12\_pool}]\cdot[\text{IL12R}] - k2_{\text{("(IM) IL12 receptor binding")}}\cdot[\text{"IL12-IL12R"}]\right)$$

$$\frac{d\left(\text{["RORgt-ligand"]}\cdot V_{ThCell}\right)}{dt} = +V_{ThCell}\cdot\left(Vf_{\text{("(IM) RORgt activation")}}\cdot\text{[RORgt]}\cdot\frac{ki1_{\text{("(IM) RORgt activation")}}^{n1_{\text{("(IM) RORgt activation")}}}}{\text{["acetylated-FOXP3"]}^{n1_{\text{("(IM) RORgt activation")}}}+ki1_{\text{("(IM) RORgt activation")}}^{n1_{\text{("(IM) RORgt activation")}}}}\cdot\left(\frac{c1_{\text{("(IM) RORgt activation")}}\cdot\text{["STAT3-P"]}^{n2_{\text{("(IM) RORgt activation")}}}}{\text{["STAT3-P"]}^{n2_{\text{("(IM) RORgt activation")}}}+ka1_{\text{("(IM) RORgt activation")}}^{n2_{\text{("(IM) RORgt activation")}}}}+\frac{c2_{\text{("(IM) RORgt activation")}}\cdot\text{["TGFb-TGFbR"]}^{n3_{\text{("(IM) RORgt activation")}}}}{\text{["TGFb-TGFbR"]}^{n3_{\text{("(IM) RORgt activation")}}}+ka2_{\text{("(IM) RORgt activation")}}^{n3_{\text{("(IM) RORgt activation")}}}}\right)-Vr_{\text{("(IM) RORgt activation")}}\cdot\text{["RORgt-ligand"]}\right)$$

$$\frac{d\left(\text{["IL17-IL17R"]}\cdot V_{ThCell}\right)}{dt} = +V_{ThCell}\cdot\left(k1_{\text{("(IM) IL17 receptor binding")}}\cdot\text{[IL17\_pool]}\cdot\text{[IL17R]}-k2_{\text{("(IM) IL17 receptor binding")}}\cdot\text{["IL17-IL17R"]}\right)$$

$$\frac{d\left(\text{[IL17R]}\cdot V_{ThCell}\right)}{dt} = -V_{ThCell}\cdot\left(k1_{\text{("(IM) IL17 receptor binding")}}\cdot\text{[IL17\_pool]}\cdot\text{[IL17R]}-k2_{\text{("(IM) IL17 receptor binding")}}\cdot\text{["IL17-IL17R"]}\right)$$

$$\frac{d\left(\text{[IL18R]}\cdot V_{ThCell}\right)}{dt} = -V_{ThCell}\cdot\left(k1_{\text{("(IM) IL18 receptor binding")}}\cdot\text{[IL18\_pool]}\cdot\text{[IL18R]}-k2_{\text{("(IM) IL18 receptor binding")}}\cdot\text{["IL18-IL18R"]}\right)$$

$$\frac{d\left(\text{["STAT6-P"]}\cdot V_{ThCell}\right)}{dt} = +V_{ThCell}\cdot\left(Vf_{\text{("(IM) STAT6 activation")}}\cdot\text{[STAT6]}\cdot\frac{ki1_{\text{("(IM) STAT6 activation")}}^{n1_{\text{("(IM) STAT6 activation")}}}}{\text{["IFNg-IFNgR"]}^{n1_{\text{("(IM) STAT6 activation")}}}+ki1_{\text{("(IM) STAT6 activation")}}^{n1_{\text{("(IM) STAT6 activation")}}}}\cdot\frac{c1_{\text{("(IM) STAT6 activation")}}\cdot\text{["IL4-IL4R"]}^{n2_{\text{("(IM) STAT6 activation")}}}}{\text{["IL4-IL4R"]}^{n2_{\text{("(IM) STAT6 activation")}}}+ka1_{\text{("(IM) STAT6 activation")}}^{n2_{\text{("(IM) STAT6 activation")}}}}-Vr_{\text{("(IM) STAT6 activation")}}\cdot\text{["STAT6-P"]}\right)$$

$$\frac{d\left(\text{[IL21R]}\cdot V_{ThCell}\right)}{dt} = -V_{ThCell}\cdot\left(k1_{\text{("(IM) IL21 receptor binding")}}\cdot\text{[IL21\_pool]}\cdot\text{[IL21R]}-k2_{\text{("(IM) IL21 receptor binding")}}\cdot\text{["IL21-IL21R"]}\right)$$

$$\frac{d\left(\text{["acetylated-FOXP3"]}\cdot V_{ThCell}\right)}{dt} = +V_{ThCell}\cdot\left(Vf_{\text{("(IM) FOXP3 activation")}}\cdot\text{[FOXP3]}\cdot\frac{ki1_{\text{("(IM) FOXP3 activation")}}^{n1_{\text{("(IM) FOXP3 activation")}}}}{\text{["STAT6-P"]}^{n1_{\text{("(IM) FOXP3 activation")}}}+ki1_{\text{("(IM) FOXP3 activation")}}^{n1_{\text{("(IM) FOXP3 activation")}}}}\cdot\frac{ki2_{\text{("(IM) FOXP3 activation")}}^{n2_{\text{("(IM) FOXP3 activation")}}}}{\text{["STAT3-P"]}^{n2_{\text{("(IM) FOXP3 activation")}}}+ki2_{\text{("(IM) FOXP3 activation")}}^{n2_{\text{("(IM) FOXP3 activation")}}}}\cdot\frac{ki3_{\text{("(IM) FOXP3 activation")}}^{n3_{\text{("(IM) FOXP3 activation")}}}}{\text{["IL6-IL6R"]}^{n3_{\text{("(IM) FOXP3 activation")}}}+ki3_{\text{("(IM) FOXP3 activation")}}^{n3_{\text{("(IM) FOXP3 activation")}}}}\cdot\frac{ki4_{\text{("(IM) FOXP3 activation")}}^{n4_{\text{("(IM) FOXP3 activation")}}}}{\text{["STAT1-P"]}^{n4_{\text{("(IM) FOXP3 activation")}}}+ki4_{\text{("(IM) FOXP3 activation")}}^{n4_{\text{("(IM) FOXP3 activation")}}}}\cdot\left(\frac{c1_{\text{("(IM) FOXP3 activation")}}\cdot\text{["TGFb-TGFbR"]}^{n5_{\text{("(IM) FOXP3 activation")}}}}{\text{["TGFb-TGFbR"]}^{n5_{\text{("(IM) FOXP3 activation")}}}+ka1_{\text{("(IM) FOXP3 activation")}}^{n5_{\text{("(IM) FOXP3 activation")}}}}+\frac{c2_{\text{("(IM) FOXP3 activation")}}\cdot\text{["STAT5-P"]}^{n6_{\text{("(IM) FOXP3 activation")}}}}{\text{["STAT5-P"]}^{n6_{\text{("(IM) FOXP3 activation")}}}+ka2_{\text{("(IM) FOXP3 activation")}}^{n6_{\text{("(IM) FOXP3 activation")}}}}+\frac{c3_{\text{("(IM) FOXP3 activation")}}\cdot\text{["L-PPARg"]}^{n7_{\text{("(IM) FOXP3 activation")}}}}{\text{["L-PPARg"]}^{n7_{\text{("(IM) FOXP3 activation")}}}+ka3_{\text{("(IM) FOXP3 activation")}}^{n7_{\text{("(IM) FOXP3 activation")}}}}+\frac{c4_{\text{("(IM) FOXP3 activation")}}\cdot\text{["TNFa-TNFaR"]}^{n8_{\text{("(IM) FOXP3 activation")}}}}{\text{["TNFa-TNFaR"]}^{n8_{\text{("(IM) FOXP3 activation")}}}+ka4_{\text{("(IM) FOXP3 activation")}}^{n8_{\text{("(IM) FOXP3 activation")}}}}+\frac{c5_{\text{("(IM) FOXP3 activation")}}\cdot\text{["IL10-IL10R"]}^{n9_{\text{("(IM) FOXP3 activation")}}}}{\text{["IL10-IL10R"]}^{n9_{\text{("(IM) FOXP3 activation")}}}+ka5_{\text{("(IM) FOXP3 activation")}}^{n9_{\text{("(IM) FOXP3 activation")}}}}+\frac{c7_{\text{("(IM) FOXP3 activation")}}\cdot\text{["LANCL2-ABA"]}^{n11_{\text{("(IM) FOXP3 activation")}}}}{\text{["LANCL2-ABA"]}^{n11_{\text{("(IM) FOXP3 activation")}}}+ka7_{\text{("(IM) FOXP3 activation")}}^{n11_{\text{("(IM) FOXP3 activation")}}}}\right)-Vr_{\text{("(IM) FOXP3 activation")}}\cdot\text{["acetylated-FOXP3"]}\right)$$

$$\frac{d\left(\text{["IL2-IL2R"]}\cdot V_{ThCell}\right)}{dt} = +V_{ThCell}\cdot\left(k1_{\text{("(IM) IL2 receptor binding")}}\cdot\text{[IL2\_pool]}\cdot\text{[IL2R]}-k2_{\text{("(IM) IL2 receptor binding")}}\cdot\text{["IL2-IL2R"]}\right)$$

$$\frac{d\left(\text{[IL2R]}\cdot V_{ThCell}\right)}{dt} = -V_{ThCell}\cdot\left(k1_{\text{("(IM) IL2 receptor binding")}}\cdot\text{[IL2\_pool]}\cdot\text{[IL2R]}-k2_{\text{("(IM) IL2 receptor binding")}}\cdot\text{["IL2-IL2R"]}\right)$$

$$\frac{d\left(["\text{STAT1-P}"]\cdot V_{ThCell}\right)}{dt} = +V_{ThCell}\cdot\left(Vf_{\text{("(IM) STAT1 activation")}}\cdot[\text{STAT1}]\cdot\frac{ki1_{\text{("(IM) STAT1 activation")}}^{n1_{\text{("(IM) STAT1 activation")}}}}{["\text{L-PPARg}"]^{n1_{\text{("(IM) STAT1 activation")}}}+ki1_{\text{("(IM) STAT1 activation")}}^{n1_{\text{("(IM) STAT1 activation")}}}}\cdot\left(\frac{c1_{\text{("(IM) STAT1 activation")}}\cdot["\text{JAK1-P}"]^{n2_{\text{("(IM) STAT1 activation")}}}}{["\text{JAK1-P}"]^{n2_{\text{("(IM) STAT1 activation")}}}+ka1_{\text{("(IM) STAT1 activation")}}^{n2_{\text{("(IM) STAT1 activation")}}}}+\frac{c2_{\text{("(IM) STAT1 activation")}}\cdot["\text{IL21-IL21R}"]^{n3_{\text{("(IM) STAT1 activation")}}}}{["\text{IL21-IL21R}"]^{n3_{\text{("(IM) STAT1 activation")}}}+ka2_{\text{("(IM) STAT1 activation")}}^{n3_{\text{("(IM) STAT1 activation")}}}}+\frac{c3_{\text{("(IM) STAT1 activation")}}\cdot["\text{IFNg-IFNgR}"]^{n4_{\text{("(IM) STAT1 activation")}}}}{["\text{IFNg-IFNgR}"]^{n4_{\text{("(IM) STAT1 activation")}}}+ka3_{\text{("(IM) STAT1 activation")}}^{n4_{\text{("(IM) STAT1 activation")}}}}\right)-Vr_{\text{("(IM) STAT1 activation")}}\cdot["\text{STAT1-P}"]\right)$$

$$\frac{d\left(["\text{IL4-IL4R}"]\cdot V_{ThCell}\right)}{dt} = +V_{ThCell}\cdot\left(k1_{\text{("(IM) IL4 receptor binding")}}\cdot[\text{IL4\_pool}]\cdot[\text{IL4R}]-k2_{\text{("(IM) IL4 receptor binding")}}\cdot["\text{IL4-IL4R}"]\right)$$

$$\frac{d\left([\text{IL4R}]\cdot V_{ThCell}\right)}{dt} = -V_{ThCell}\cdot\left(k1_{\text{("(IM) IL4 receptor binding")}}\cdot[\text{IL4\_pool}]\cdot[\text{IL4R}]-k2_{\text{("(IM) IL4 receptor binding")}}\cdot["\text{IL4-IL4R}"]\right)$$

$$\frac{d\left(["\text{IL6-IL6R}"]\cdot V_{ThCell}\right)}{dt} = +V_{ThCell}\cdot\left(k1_{\text{("(IM) IL6 receptor binding")}}\cdot[\text{IL6\_pool}]\cdot[\text{IL6R}]-k2_{\text{("(IM) IL6 receptor binding")}}\cdot["\text{IL6-IL6R}"]\right)$$

$$\frac{d\left([\text{IL6R}]\cdot V_{ThCell}\right)}{dt} = -V_{ThCell}\cdot\left(k1_{\text{("(IM) IL6 receptor binding")}}\cdot[\text{IL6\_pool}]\cdot[\text{IL6R}]-k2_{\text{("(IM) IL6 receptor binding")}}\cdot["\text{IL6-IL6R}"]\right)$$

$$\frac{d\left([\text{IL23R}]\cdot V_{ThCell}\right)}{dt} = -V_{ThCell}\cdot\left(k1_{\text{("(IM) IL23 receptor binding")}}\cdot[\text{IL23\_pool}]\cdot[\text{IL23R}]-k2_{\text{("(IM) IL23 receptor binding")}}\cdot["\text{IL23-IL23R}"]\right)$$

$$\frac{d\left(["\text{IRAK1-P}"]\cdot V_{ThCell}\right)}{dt} = +V_{ThCell}\cdot\left(Vf_{\text{("(IM) IRAK1 activation")}}\cdot[\text{IRAK1}]\cdot\frac{c1_{\text{("(IM) IRAK1 activation")}}\cdot["\text{IL18-IL18R}"]^{n1_{\text{("(IM) IRAK1 activation")}}}}{["\text{IL18-IL18R}"]^{n1_{\text{("(IM) IRAK1 activation")}}}+ka1_{\text{("(IM) IRAK1 activation")}}^{n1_{\text{("(IM) IRAK1 activation")}}}}-Vr_{\text{("(IM) IRAK1 activation")}}\cdot["\text{IRAK1-P}"]\right)$$

$$\frac{d\left(["\text{TGFb-TGFbR}"]\cdot V_{ThCell}\right)}{dt} = +V_{ThCell}\cdot\left(k1_{\text{("(IM) TGFb receptor binding")}}\cdot[\text{TGFb\_pool}]\cdot[\text{TGFbR}]-k2_{\text{("(IM) TGFb receptor binding")}}\cdot["\text{TGFb-TGFbR}"]\right)$$

$$\frac{d\left([\text{TGFbR}]\cdot V_{ThCell}\right)}{dt} = -V_{ThCell}\cdot\left(k1_{\text{("(IM) TGFb receptor binding")}}\cdot[\text{TGFb\_pool}]\cdot[\text{TGFbR}]-k2_{\text{("(IM) TGFb receptor binding")}}\cdot["\text{TGFb-TGFbR}"]\right)$$

$$\frac{d\left(["\text{IL9-IL9R}"]\cdot V_{ThCell}\right)}{dt} = +V_{ThCell}\cdot\left(k1_{\text{("(IM) IL9 receptor binding")}}\cdot[\text{IL9\_pool}]\cdot[\text{IL9R}]-k2_{\text{("(IM) IL9 receptor binding")}}\cdot["\text{IL9-IL9R}"]\right)$$

$$\frac{d\left([\text{IL9R}]\cdot V_{\text{ThCell}}\right)}{dt} = -V_{\text{ThCell}}\cdot\left(k1_{\text{("(IM) IL9 receptor binding")}}\cdot[\text{IL9\_pool}]\cdot[\text{IL9R}]-k2_{\text{("(IM) IL9 receptor binding")}}\cdot[\text{"IL9-IL9R"}]\right)$$

$$\frac{d\left([\text{"IL22-IL22R"}]\cdot V_{\text{ThCell}}\right)}{dt} = +V_{\text{ThCell}}\cdot\left(k1_{\text{("(IM) IL22 receptor binding")}}\cdot[\text{IL22}]\cdot[\text{IL22R}]-k2_{\text{("(IM) IL22 receptor binding")}}\cdot[\text{"IL22-IL22R"}]\right)$$

$$\frac{d\left([\text{IL22R}]\cdot V_{\text{ThCell}}\right)}{dt} = -V_{\text{ThCell}}\cdot\left(k1_{\text{("(IM) IL22 receptor binding")}}\cdot[\text{IL22}]\cdot[\text{IL22R}]-k2_{\text{("(IM) IL22 receptor binding")}}\cdot[\text{"IL22-IL22R"}]\right)$$

$$\frac{d\left([\text{"TNFa-TNFaR"}]\cdot V_{\text{ThCell}}\right)}{dt} = +V_{\text{ThCell}}\cdot\left(k1_{\text{("(IM) TNFa receptor binding")}}\cdot[\text{TNFa\_pool}]\cdot[\text{TNFaR}]-k2_{\text{("(IM) TNFa receptor binding")}}\cdot[\text{"TNFa-TNFaR"}]\right)$$

$$\frac{d\left([\text{TNFaR}]\cdot V_{\text{ThCell}}\right)}{dt} = -V_{\text{ThCell}}\cdot\left(k1_{\text{("(IM) TNFa receptor binding")}}\cdot[\text{TNFa\_pool}]\cdot[\text{TNFaR}]-k2_{\text{("(IM) TNFa receptor binding")}}\cdot[\text{"TNFa-TNFaR"}]\right)$$

$$\frac{d\left([\text{STAT4}]\cdot V_{\text{ThCell}}\right)}{dt} = -V_{\text{ThCell}}\cdot\left(Vf_{\text{("(IM) STAT4 activation")}}\cdot[\text{STAT4}]\cdot\frac{ki1_{\text{("(IM) STAT4 activation")}}^{n1_{\text{("(IM) STAT4 activation")}}}}{[\text{"IL21-IL21R"}]^{n1_{\text{("(IM) STAT4 activation")}}}+ki1_{\text{("(IM) STAT4 activation")}}^{n1_{\text{("(IM) STAT4 activation")}}}}\cdot\frac{ki2_{\text{("(IM) STAT4 activation")}}^{n2_{\text{("(IM) STAT4 activation")}}}}{[\text{"GATA3-P"}]^{n2_{\text{("(IM) STAT4 activation")}}}+ki2_{\text{("(IM) STAT4 activation")}}^{n2_{\text{("(IM) STAT4 activation")}}}}\cdot\left(\frac{c1_{\text{("(IM) STAT4 activation")}}\cdot[\text{"IL12-IL12R"}]^{n3_{\text{("(IM) STAT4 activation")}}}}{[\text{"IL12-IL12R"}]^{n3_{\text{("(IM) STAT4 activation")}}}+ka1_{\text{("(IM) STAT4 activation")}}^{n3_{\text{("(IM) STAT4 activation")}}}}+\frac{c2_{\text{("(IM) STAT4 activation")}}\cdot[\text{"IL23-IL23R"}]^{n4_{\text{("(IM) STAT4 activation")}}}}{[\text{"IL23-IL23R"}]^{n4_{\text{("(IM) STAT4 activation")}}}+ka2_{\text{("(IM) STAT4 activation")}}^{n4_{\text{("(IM) STAT4 activation")}}}}\right)-Vr_{\text{("(IM) STAT4 activation")}}\cdot[\text{"STAT4-P"}]\right)$$

$$\frac{d\left([\text{"JAK1-P"}]\cdot V_{\text{ThCell}}\right)}{dt} = +V_{\text{ThCell}}\cdot\left(Vf_{\text{("(IM) JAK1 activation")}}\cdot[\text{JAK1}]\cdot\frac{ki1_{\text{("(IM) JAK1 activation")}}^{n1_{\text{("(IM) JAK1 activation")}}}}{[\text{"L-PPARg"}]^{n1_{\text{("(IM) JAK1 activation")}}}+ki1_{\text{("(IM) JAK1 activation")}}^{n1_{\text{("(IM) JAK1 activation")}}}}\cdot\frac{ki2_{\text{("(IM) JAK1 activation")}}^{n2_{\text{("(IM) JAK1 activation")}}}}{[\text{"SOCS1-JAKs"}]^{n2_{\text{("(IM) JAK1 activation")}}}+ki2_{\text{("(IM) JAK1 activation")}}^{n2_{\text{("(IM) JAK1 activation")}}}}\cdot\frac{c1_{\text{("(IM) JAK1 activation")}}\cdot[\text{"IFNg-IFNgR"}]^{n3_{\text{("(IM) JAK1 activation")}}}}{[\text{"IFNg-IFNgR"}]^{n3_{\text{("(IM) JAK1 activation")}}}+ka1_{\text{("(IM) JAK1 activation")}}^{n3_{\text{("(IM) JAK1 activation")}}}}-Vr_{\text{("(IM) JAK1 activation")}}\cdot[\text{"JAK1-P"}]\right)$$

$$\frac{d\left([\text{STAT1}]\cdot V_{\text{ThCell}}\right)}{dt} = -V_{\text{ThCell}}\cdot\left(Vf_{\text{("(IM) STAT1 activation")}}\cdot[\text{STAT1}]\cdot\frac{ki1_{\text{("(IM) STAT1 activation")}}^{n1_{\text{("(IM) STAT1 activation")}}}}{[\text{"L-PPARg"}]^{n1_{\text{("(IM) STAT1 activation")}}}+ki1_{\text{("(IM) STAT1 activation")}}^{n1_{\text{("(IM) STAT1 activation")}}}}\cdot\left(\frac{c1_{\text{("(IM) STAT1 activation")}}\cdot[\text{"JAK1-P"}]^{n2_{\text{("(IM) STAT1 activation")}}}}{[\text{"JAK1-P"}]^{n2_{\text{("(IM) STAT1 activation")}}}+ka1_{\text{("(IM) STAT1 activation")}}^{n2_{\text{("(IM) STAT1 activation")}}}}+\frac{c2_{\text{("(IM) STAT1 activation")}}\cdot[\text{"IL21-IL21R"}]^{n3_{\text{("(IM) STAT1 activation")}}}}{[\text{"IL21-IL21R"}]^{n3_{\text{("(IM) STAT1 activation")}}}+ka2_{\text{("(IM) STAT1 activation")}}^{n3_{\text{("(IM) STAT1 activation")}}}}+\frac{c3_{\text{("(IM) STAT1 activation")}}\cdot[\text{"IFNg-IFNgR"}]^{n4_{\text{("(IM) STAT1 activation")}}}}{[\text{"IFNg-IFNgR"}]^{n4_{\text{("(IM) STAT1 activation")}}}+ka3_{\text{("(IM) STAT1 activation")}}^{n4_{\text{("(IM) STAT1 activation")}}}}\right)-Vr_{\text{("(IM) STAT1 activation")}}\cdot[\text{"STAT1-P"}]\right)$$

$$\frac{d\left([\text{Tbet}]\cdot V_{\text{ThCell}}\right)}{dt} = -V_{\text{ThCell}}\cdot\left(Vf_{\text{("(IM) Tbet activation")}}\cdot[\text{Tbet}]\cdot\frac{ki1_{\text{("(IM) Tbet activation")}}^{n1_{\text{("(IM) Tbet activation")}}}}{[\text{"TGFb-TGFbR"}]^{n1_{\text{("(IM) Tbet activation")}}}+ki1_{\text{("(IM) Tbet activation")}}^{n1_{\text{("(IM) Tbet activation")}}}}\cdot\frac{ki2_{\text{("(IM) Tbet activation")}}^{n2_{\text{("(IM) Tbet activation")}}}}{[\text{"STAT3-P"}]^{n2_{\text{("(IM) Tbet activation")}}}+ki2_{\text{("(IM) Tbet activation")}}^{n2_{\text{("(IM) Tbet activation")}}}}\cdot\frac{ki3_{\text{("(IM) Tbet activation")}}^{n3_{\text{("(IM) Tbet activation")}}}}{[\text{"GATA3-P"}]^{n3_{\text{("(IM) Tbet activation")}}}+ki3_{\text{("(IM) Tbet activation")}}^{n3_{\text{("(IM) Tbet activation")}}}}\cdot\frac{ki4_{\text{("(IM) Tbet activation")}}^{n4_{\text{("(IM) Tbet activation")}}}}{[\text{"LANCL2-ABA"}]^{n4_{\text{("(IM) Tbet activation")}}}+ki4_{\text{("(IM) Tbet activation")}}^{n4_{\text{("(IM) Tbet activation")}}}}\cdot\left(\frac{c1_{\text{("(IM) Tbet activation")}}\cdot[\text{"STAT4-P"}]^{n5_{\text{("(IM) Tbet activation")}}}}{[\text{"STAT4-P"}]^{n5_{\text{("(IM) Tbet activation")}}}+ka1_{\text{("(IM) Tbet activation")}}^{n5_{\text{("(IM) Tbet activation")}}}}+\frac{c2_{\text{("(IM) Tbet activation")}}\cdot[\text{"STAT1-P"}]^{n6_{\text{("(IM) Tbet activation")}}}}{[\text{"STAT1-P"}]^{n6_{\text{("(IM) Tbet activation")}}}+ka2_{\text{("(IM) Tbet activation")}}^{n6_{\text{("(IM) Tbet activation")}}}}+\frac{c3_{\text{("(IM) Tbet activation")}}\cdot[\text{"IFNg-IFNgR"}]^{n7_{\text{("(IM) Tbet activation")}}}}{[\text{"IFNg-IFNgR"}]^{n7_{\text{("(IM) Tbet activation")}}}+ka3_{\text{("(IM) Tbet activation")}}^{n7_{\text{("(IM) Tbet activation")}}}}+\frac{c4_{\text{("(IM) Tbet activation")}}\cdot[\text{"IL2-IL2R"}]^{n8_{\text{("(IM) Tbet activation")}}}}{[\text{"IL2-IL2R"}]^{n8_{\text{("(IM) Tbet activation")}}}+ka4_{\text{("(IM) Tbet activation")}}^{n8_{\text{("(IM) Tbet activation")}}}}\right)-Vr_{\text{("(IM) Tbet activation")}}\cdot[\text{"Tbet-P"}]\right)$$

$$\frac{d([JAK1]\cdot V_{ThCell})}{dt} = -V_{ThCell}\cdot\left(Vf_{\text{("(IM) JAK1 activation")}}\cdot[JAK1]\cdot\frac{ki1_{\text{("(IM) JAK1 activation")}}^{n1_{\text{("(IM) JAK1 activation")}}}}{[\text{"L-PPARg"}]^{n1_{\text{("(IM) JAK1 activation")}}}+ki1_{\text{("(IM) JAK1 activation")}}^{n1_{\text{("(IM) JAK1 activation")}}}}\cdot\frac{ki2_{\text{("(IM) JAK1 activation")}}^{n2_{\text{("(IM) JAK1 activation")}}}}{[\text{"SOCS1-JAKs"}]^{n2_{\text{("(IM) JAK1 activation")}}}+ki2_{\text{("(IM) JAK1 activation")}}^{n2_{\text{("(IM) JAK1 activation")}}}}\cdot\frac{c1_{\text{("(IM) JAK1 activation")}}\cdot[\text{"IFNg-IFNgR"}]^{n3_{\text{("(IM) JAK1 activation")}}}}{[\text{"IFNg-IFNgR"}]^{n3_{\text{("(IM) JAK1 activation")}}}+ka1_{\text{("(IM) JAK1 activation")}}^{n3_{\text{("(IM) JAK1 activation")}}}}-Vr_{\text{("(IM) JAK1 activation")}}\cdot[\text{"JAK1-P"}]\right)$$

$$\frac{d([SOCS1]\cdot V_{ThCell})}{dt} = -V_{ThCell}\cdot\left(Vf_{\text{("(IM) SOCS1 activation")}}\cdot[SOCS1]\cdot\left(\frac{c1_{\text{("(IM) SOCS1 activation")}}\cdot[\text{"STAT1-P"}]^{n1_{\text{("(IM) SOCS1 activation")}}}}{[\text{"STAT1-P"}]^{n1_{\text{("(IM) SOCS1 activation")}}}+ka1_{\text{("(IM) SOCS1 activation")}}^{n1_{\text{("(IM) SOCS1 activation")}}}}+\frac{c2_{\text{("(IM) SOCS1 activation")}}\cdot[\text{"Tbet-P"}]^{n2_{\text{("(IM) SOCS1 activation")}}}}{[\text{"Tbet-P"}]^{n2_{\text{("(IM) SOCS1 activation")}}}+ka2_{\text{("(IM) SOCS1 activation")}}^{n2_{\text{("(IM) SOCS1 activation")}}}}\right)-Vr_{\text{("(IM) SOCS1 activation")}}\cdot[\text{"SOCS1-JAKs"}]\right)$$

$$\frac{d([GATA3]\cdot V_{ThCell})}{dt} = -V_{ThCell}\cdot\left(Vf_{\text{("(IM) GATA3 activation")}}\cdot[GATA3]\cdot\frac{ki1_{\text{("(IM) GATA3 activation")}}^{n1_{\text{("(IM) GATA3 activation")}}}}{[\text{"Tbet-P"}]^{n1_{\text{("(IM) GATA3 activation")}}}+ki1_{\text{("(IM) GATA3 activation")}}^{n1_{\text{("(IM) GATA3 activation")}}}}\cdot\frac{ki2_{\text{("(IM) GATA3 activation")}}^{n2_{\text{("(IM) GATA3 activation")}}}}{[\text{"STAT3-P"}]^{n2_{\text{("(IM) GATA3 activation")}}}+ki2_{\text{("(IM) GATA3 activation")}}^{n2_{\text{("(IM) GATA3 activation")}}}}\cdot\frac{ki3_{\text{("(IM) GATA3 activation")}}^{n3_{\text{("(IM) GATA3 activation")}}}}{[\text{"TGFb-TGFbR"}]^{n3_{\text{("(IM) GATA3 activation")}}}+ki3_{\text{("(IM) GATA3 activation")}}^{n3_{\text{("(IM) GATA3 activation")}}}}\cdot\left(\frac{c1_{\text{("(IM) GATA3 activation")}}\cdot[\text{"STAT6-P"}]^{n4_{\text{("(IM) GATA3 activation")}}}}{[\text{"STAT6-P"}]^{n4_{\text{("(IM) GATA3 activation")}}}+ka1_{\text{("(IM) GATA3 activation")}}^{n4_{\text{("(IM) GATA3 activation")}}}}+\frac{c2_{\text{("(IM) GATA3 activation")}}\cdot[\text{"L-PPARg"}]^{n5_{\text{("(IM) GATA3 activation")}}}}{[\text{"L-PPARg"}]^{n5_{\text{("(IM) GATA3 activation")}}}+ka2_{\text{("(IM) GATA3 activation")}}^{n5_{\text{("(IM) GATA3 activation")}}}}\right)-Vr_{\text{("(IM) GATA3 activation")}}\cdot[\text{"GATA3-P"}]\right)$$

$$\frac{d([STAT6]\cdot V_{ThCell})}{dt} = -V_{ThCell}\cdot\left(Vf_{\text{("(IM) STAT6 activation")}}\cdot[STAT6]\cdot\frac{ki1_{\text{("(IM) STAT6 activation")}}^{n1_{\text{("(IM) STAT6 activation")}}}}{[\text{"IFNg-IFNgR"}]^{n1_{\text{("(IM) STAT6 activation")}}}+ki1_{\text{("(IM) STAT6 activation")}}^{n1_{\text{("(IM) STAT6 activation")}}}}\cdot\frac{c1_{\text{("(IM) STAT6 activation")}}\cdot[\text{"IL4-IL4R"}]^{n2_{\text{("(IM) STAT6 activation")}}}}{[\text{"IL4-IL4R"}]^{n2_{\text{("(IM) STAT6 activation")}}}+ka1_{\text{("(IM) STAT6 activation")}}^{n2_{\text{("(IM) STAT6 activation")}}}}-Vr_{\text{("(IM) STAT6 activation")}}\cdot[\text{"STAT6-P"}]\right)$$

$$\frac{d([STAT5]\cdot V_{ThCell})}{dt} = -V_{ThCell}\cdot\left(Vf_{\text{("(IM) STAT5 activation")}}\cdot[STAT5]\cdot\left(\frac{c1_{\text{("(IM) STAT5 activation")}}\cdot[\text{"IL2-IL2R"}]^{n1_{\text{("(IM) STAT5 activation")}}}}{[\text{"IL2-IL2R"}]^{n1_{\text{("(IM) STAT5 activation")}}}+ka1_{\text{("(IM) STAT5 activation")}}^{n1_{\text{("(IM) STAT5 activation")}}}}+\frac{c2_{\text{("(IM) STAT5 activation")}}\cdot[\text{"LANCL2-ABA"}]^{n2_{\text{("(IM) STAT5 activation")}}}}{[\text{"LANCL2-ABA"}]^{n2_{\text{("(IM) STAT5 activation")}}}+ka_{\text{("(IM) STAT5 activation")}}^{n2_{\text{("(IM) STAT5 activation")}}}}\right)-Vr_{\text{("(IM) STAT5 activation")}}\cdot[\text{"STAT5-P"}]\right)$$

$$\frac{d([FOXP3]\cdot V_{ThCell})}{dt} = -V_{ThCell}\cdot\left(Vf_{\text{("(IM) FOXP3 activation")}}\cdot[FOXP3]\cdot\frac{ki1_{\text{("(IM) FOXP3 activation")}}^{n1_{\text{("(IM) FOXP3 activation")}}}}{[\text{"STAT6-P"}]^{n1_{\text{("(IM) FOXP3 activation")}}}+ki1_{\text{("(IM) FOXP3 activation")}}^{n1_{\text{("(IM) FOXP3 activation")}}}}\cdot\frac{ki2_{\text{("(IM) FOXP3 activation")}}^{n2_{\text{("(IM) FOXP3 activation")}}}}{[\text{"STAT3-P"}]^{n2_{\text{("(IM) FOXP3 activation")}}}+ki2_{\text{("(IM) FOXP3 activation")}}^{n2_{\text{("(IM) FOXP3 activation")}}}}\cdot\frac{ki3_{\text{("(IM) FOXP3 activation")}}^{n3_{\text{("(IM) FOXP3 activation")}}}}{[\text{"IL6-IL6R"}]^{n3_{\text{("(IM) FOXP3 activation")}}}+ki3_{\text{("(IM) FOXP3 activation")}}^{n3_{\text{("(IM) FOXP3 activation")}}}}\cdot\frac{ki4_{\text{("(IM) FOXP3 activation")}}^{n4_{\text{("(IM) FOXP3 activation")}}}}{[\text{"STAT1-P"}]^{n4_{\text{("(IM) FOXP3 activation")}}}+ki4_{\text{("(IM) FOXP3 activation")}}^{n4_{\text{("(IM) FOXP3 activation")}}}}\cdot\left(\frac{c1_{\text{("(IM) FOXP3 activation")}}\cdot[\text{"TGFb-TGFbR"}]^{n5_{\text{("(IM) FOXP3 activation")}}}}{[\text{"TGFb-TGFbR"}]^{n5_{\text{("(IM) FOXP3 activation")}}}+ka1_{\text{("(IM) FOXP3 activation")}}^{n5_{\text{("(IM) FOXP3 activation")}}}}+\frac{c2_{\text{("(IM) FOXP3 activation")}}\cdot[\text{"STAT5-P"}]^{n6_{\text{("(IM) FOXP3 activation")}}}}{[\text{"STAT5-P"}]^{n6_{\text{("(IM) FOXP3 activation")}}}+ka2_{\text{("(IM) FOXP3 activation")}}^{n6_{\text{("(IM) FOXP3 activation")}}}}+\frac{c3_{\text{("(IM) FOXP3 activation")}}\cdot[\text{"L-PPARg"}]^{n7_{\text{("(IM) FOXP3 activation")}}}}{[\text{"L-PPARg"}]^{n7_{\text{("(IM) FOXP3 activation")}}}+ka3_{\text{("(IM) FOXP3 activation")}}^{n7_{\text{("(IM) FOXP3 activation")}}}}+\frac{c4_{\text{("(IM) FOXP3 activation")}}\cdot[\text{"TNFa-TNFaR"}]^{n8_{\text{("(IM) FOXP3 activation")}}}}{[\text{"TNFa-TNFaR"}]^{n8_{\text{("(IM) FOXP3 activation")}}}+ka4_{\text{("(IM) FOXP3 activation")}}^{n8_{\text{("(IM) FOXP3 activation")}}}}+\frac{c5_{\text{("(IM) FOXP3 activation")}}\cdot[\text{"IL10-IL10R"}]^{n9_{\text{("(IM) FOXP3 activation")}}}}{[\text{"IL10-IL10R"}]^{n9_{\text{("(IM) FOXP3 activation")}}}+ka5_{\text{("(IM) FOXP3 activation")}}^{n9_{\text{("(IM) FOXP3 activation")}}}}+\frac{c7_{\text{("(IM) FOXP3 activation")}}\cdot[\text{"LANCL2-ABA"}]^{n11_{\text{("(IM) FOXP3 activation")}}}}{[\text{"LANCL2-ABA"}]^{n11_{\text{("(IM) FOXP3 activation")}}}+ka7_{\text{("(IM) FOXP3 activation")}}^{n11_{\text{("(IM) FOXP3 activation")}}}}\right)-Vr_{\text{("(IM) FOXP3 activation")}}\cdot[\text{"acetylated-FOXP3"}]\right)$$

$$\frac{d([IRAK1]\cdot V_{ThCell})}{dt} = -V_{ThCell}\cdot\left(Vf_{\text{("(IM) IRAK1 activation")}}\cdot[IRAK1]\cdot\frac{c1_{\text{("(IM) IRAK1 activation")}}\cdot[\text{"IL18-IL18R"}]^{n1_{\text{("(IM) IRAK1 activation")}}}}{[\text{"IL18-IL18R"}]^{n1_{\text{("(IM) IRAK1 activation")}}}+ka1_{\text{("(IM) IRAK1 activation")}}^{n1_{\text{("(IM) IRAK1 activation")}}}}-Vr_{\text{("(IM) IRAK1 activation")}}\cdot[\text{"IRAK1-P"}]\right)$$

$$\frac{d([\text{STAT3}]\cdot V_{\text{ThCell}})}{d\,t} = -V_{\text{ThCell}}\cdot\left(Vf_{\text{("(IM) STAT3 activation")}}\cdot[\text{STAT3}]\cdot\frac{ki1_{\text{("(IM) STAT3 activation")}}^{n1_{\text{("(IM) STAT3 activation")}}}}{[\text{"L-PPARg"}]^{n1_{\text{("(IM) STAT3 activation")}}}+ki1_{\text{("(IM) STAT3 activation")}}^{n1_{\text{("(IM) STAT3 activation")}}}}\cdot\left(\frac{c1_{\text{("(IM) STAT3 activation")}}\cdot[\text{"IL21-IL21R"}]^{n2_{\text{("(IM) STAT3 activation")}}}}{[\text{"IL21-IL21R"}]^{n2_{\text{("(IM) STAT3 activation")}}}+ka1_{\text{("(IM) STAT3 activation")}}^{n2_{\text{("(IM) STAT3 activation")}}}}+\frac{c2_{\text{("(IM) STAT3 activation")}}\cdot[\text{"IL23-IL23R"}]^{n3_{\text{("(IM) STAT3 activation")}}}}{[\text{"IL23-IL23R"}]^{n3_{\text{("(IM) STAT3 activation")}}}+ka2_{\text{("(IM) STAT3 activation")}}^{n3_{\text{("(IM) STAT3 activation")}}}}+\frac{c3_{\text{("(IM) STAT3 activation")}}\cdot[\text{Lactate\{Cytosol\}}]^{n4_{\text{("(IM) STAT3 activation")}}}}{[\text{Lactate\{Cytosol\}}]^{n4_{\text{("(IM) STAT3 activation")}}}+ka3_{\text{("(IM) STAT3 activation")}}^{n4_{\text{("(IM) STAT3 activation")}}}}+\frac{c4_{\text{("(IM) STAT3 activation")}}\cdot[\text{"IL6-IL6R"}]^{n5_{\text{("(IM) STAT3 activation")}}}}{[\text{"IL6-IL6R"}]^{n5_{\text{("(IM) STAT3 activation")}}}+ka4_{\text{("(IM) STAT3 activation")}}^{n5_{\text{("(IM) STAT3 activation")}}}}+\frac{c5_{\text{("(IM) STAT3 activation")}}\cdot[\text{"IL22-IL22R"}]^{n6_{\text{("(IM) STAT3 activation")}}}}{[\text{"IL22-IL22R"}]^{n6_{\text{("(IM) STAT3 activation")}}}+ka5_{\text{("(IM) STAT3 activation")}}^{n6_{\text{("(IM) STAT3 activation")}}}}+\frac{c6_{\text{("(IM) STAT3 activation")}}\cdot[\text{NADPH\_tot}]^{n7_{\text{("(IM) STAT3 activation")}}}}{[\text{NADPH\_tot}]^{n7_{\text{("(IM) STAT3 activation")}}}+ka6_{\text{("(IM) STAT3 activation")}}^{n7_{\text{("(IM) STAT3 activation")}}}}\right)-Vr_{\text{("(IM) STAT3 activation")}}\cdot[\text{"STAT3-P"}]\right)$$

$$\frac{d([\text{RORgt}]\cdot V_{\text{ThCell}})}{d\,t} = -V_{\text{ThCell}}\cdot\left(Vf_{\text{("(IM) RORgt activation")}}\cdot[\text{RORgt}]\cdot\frac{ki1_{\text{("(IM) RORgt activation")}}^{n1_{\text{("(IM) RORgt activation")}}}}{[\text{"acetylated-FOXP3"}]^{n1_{\text{("(IM) RORgt activation")}}}+ki1_{\text{("(IM) RORgt activation")}}^{n1_{\text{("(IM) RORgt activation")}}}}\cdot\left(\frac{c1_{\text{("(IM) RORgt activation")}}\cdot[\text{"STAT3-P"}]^{n2_{\text{("(IM) RORgt activation")}}}}{[\text{"STAT3-P"}]^{n2_{\text{("(IM) RORgt activation")}}}+ka1_{\text{("(IM) RORgt activation")}}^{n2_{\text{("(IM) RORgt activation")}}}}+\frac{c2_{\text{("(IM) RORgt activation")}}\cdot[\text{"TGFb-TGFbR"}]^{n3_{\text{("(IM) RORgt activation")}}}}{[\text{"TGFb-TGFbR"}]^{n3_{\text{("(IM) RORgt activation")}}}+ka2_{\text{("(IM) RORgt activation")}}^{n3_{\text{("(IM) RORgt activation")}}}}\right)-Vr_{\text{("(IM) RORgt activation")}}\cdot[\text{"RORgt-ligand"}]\right)$$

$$\frac{d([\text{PPARg}]\cdot V_{\text{ThCell}})}{d\,t} = -V_{\text{ThCell}}\cdot\left(Vf_{\text{("(IM) PPARg activation")}}\cdot[\text{PPARg}]\cdot\frac{ki1_{\text{("(IM) PPARg activation")}}^{n1_{\text{("(IM) PPARg activation")}}}}{[\text{"IFNg-IFNgR"}]^{n1_{\text{("(IM) PPARg activation")}}}+ki1_{\text{("(IM) PPARg activation")}}^{n1_{\text{("(IM) PPARg activation")}}}}\cdot 1-Vr_{\text{("(IM) PPARg activation")}}\cdot[\text{"L-PPARg"}]\right)$$

$$\frac{d([\text{AhR}]\cdot V_{\text{ThCell}})}{d\,t} = -V_{\text{ThCell}}\cdot\left(Vf_{\text{("(IM) AhR activation")}}\cdot[\text{AhR}]\cdot\left(\frac{c1_{\text{("(IM) AhR activation")}}\cdot[\text{"TNFa-TNFaR"}]^{n1_{\text{("(IM) AhR activation")}}}}{[\text{"TNFa-TNFaR"}]^{n1_{\text{("(IM) AhR activation")}}}+ka1_{\text{("(IM) AhR activation")}}^{n1_{\text{("(IM) AhR activation")}}}}+\frac{c2_{\text{("(IM) AhR activation")}}\cdot[\text{"IL6-IL6R"}]^{n2_{\text{("(IM) AhR activation")}}}}{[\text{"IL6-IL6R"}]^{n2_{\text{("(IM) AhR activation")}}}+ka2_{\text{("(IM) AhR activation")}}^{n2_{\text{("(IM) AhR activation")}}}}+\frac{c3_{\text{("(IM) AhR activation")}}\cdot[\text{FICZ}]^{n3_{\text{("(IM) AhR activation")}}}}{[\text{FICZ}]^{n3_{\text{("(IM) AhR activation")}}}+ka3_{\text{("(IM) AhR activation")}}^{n3_{\text{("(IM) AhR activation")}}}}\right)-Vr_{\text{("(IM) AhR activation")}}\cdot[\text{"AhR-P"}]\right)$$

$$[\text{enz\_LANCL2}] = \text{Naive}\cdot 5.73\text{E-}08+\text{Th1}\cdot 5.66\text{E-}08+\text{Th2}\cdot 4.9\text{E-}08+\text{Th17}\cdot 5.16\text{E-}08+\text{Th22}\cdot 5.42\text{E-}08+\text{Treg}\cdot 6.71\text{E-}08-\text{"[LANCL2-ABA]"}$$

$$\frac{d([\text{"LANCL2-ABA"}]\cdot V_{\text{ThCell}})}{d\,t} = +V_{\text{ThCell}}\cdot\left(k1_{\text{("(IM) LANCL2 binding")}}\cdot[\text{ABA\_pool}]\cdot[\text{enz\_LANCL2}]-k2_{\text{("(IM) LANCL2 binding")}}\cdot[\text{"LANCL2-ABA"}]\right)$$

$$[\text{enz\_GAPDH}] = \text{Naive}\cdot 2.24\text{E-}06+\text{Th1}\cdot 4.03\text{E-}06+\text{Th2}\cdot 3.95\text{E-}06+\text{Th17}\cdot 4.60\text{E-}06+\text{Th22}\cdot 4.64\text{E-}06+\text{Treg}\cdot 5.57\text{E-}06$$

$$[\text{enz\_LDHA}] = (\text{Naive}\cdot 4.37\text{E-}07+\text{Th1}\cdot 5.45\text{E-}07+\text{Th2}\cdot 5.32\text{E-}07+\text{Th17}\cdot 5.35\text{E-}07+\text{Th22}\cdot 5.66\text{E-}07+\text{Treg}\cdot 3.93\text{E-}07)\cdot \text{ldha\_on}+(1-\text{ldha\_on})\cdot\text{temp}$$

$$\frac{d([\text{G1,5L6P}]\cdot V_{\text{Cytosol}})}{d\,t} = +V_{\text{Cytosol}}\cdot\left(kcat_{\text{("(PPP) G6PD")}}\cdot[\text{enz\_G6PD}]\cdot\frac{[\text{"G6-P"}]}{Km_{\text{("(PPP) G6PD")}}+[\text{"G6-P"}]}\right) - V_{\text{Cytosol}}\cdot\left(kcat_{\text{("(PPP) PGL")}}\cdot[\text{enz\_PGL}]\cdot\frac{[\text{G1,5L6P}]}{Km_{\text{("(PPP) PGL")}}+[\text{G1,5L6P}]}\right)$$

$$[\text{enz\_PGL}] = \text{Naive}\cdot 2.22\text{E-}07+\text{Th1}\cdot 2.27\text{E-}07+\text{Th2}\cdot 2.53\text{E-}07+\text{Th17}\cdot 2.58\text{E-}07+\text{Th22}\cdot 2.24\text{E-}07+\text{Treg}\cdot 2.81\text{E-}07$$

$$\frac{d\left(\text{[Malate\{Mitochondria\}]}\cdot V_{\text{Mitochondria}}\right)}{d\,t} = -V_{\text{Mitochondria}}\cdot\left(\text{kcat}_{\text{("(TCA) MDH2")}}\cdot\text{[enz\_MDH2]}\cdot\frac{\text{[Malate\{Mitochondria\}]}\cdot\text{[NAD\_S\{Mitochondria\}]}}{\text{KmB}_{\text{("(TCA) MDH2")}}\cdot\text{[Malate\{Mitochondria\}]}+\text{KmA}_{\text{("(TCA) MDH2")}}\cdot\text{[NAD\_S\{Mitochondria\}]}+\text{[Malate\{Mitochondria\}]}\cdot\text{[NAD\_S\{Mitochondria\}]}+0.0000001}\right)$$

$$+V_{\text{Mitochondria}}\cdot\left(\text{kcat}_{\text{("(TCA) FH\_F")}}\cdot\text{[enz\_FH]}\cdot\frac{\text{[Fumarate]}}{\text{Km}_{\text{("(TCA) FH\_F")}}+\text{[Fumarate]}}\right)$$

$$-V_{\text{Mitochondria}}\cdot\left(\text{kcat}_{\text{("(TCA) ME")}}\cdot\text{[enz\_ME2]}\cdot\frac{\text{[Malate\{Mitochondria\}]}}{\text{Km}_{\text{("(TCA) ME")}}+\text{[Malate\{Mitochondria\}]}}\right)$$

$$-V_{\text{Mitochondria}}\cdot\left(\text{kcat}_{\text{("(TCA) FH\_R")}}\cdot\text{[enz\_FH]}\cdot\frac{\text{[Malate\{Mitochondria\}]}}{\text{Km}_{\text{("(TCA) FH\_R")}}+\text{[Malate\{Mitochondria\}]}}\right)$$

$$+V_{\text{Cytosol}}\cdot\left(\text{kcat}_{\text{("(MAS) M-aKG Transport")}}\cdot\text{[enz\_SLC25A11]}\cdot\frac{\text{[Malate\{Cytosol\}]}\cdot\text{["a-KG\{Mitochondria\}"]}}{\text{KmB}_{\text{("(MAS) M-aKG Transport")}}\cdot\text{[Malate\{Cytosol\}]}+\text{KmA}_{\text{("(MAS) M-aKG Transport")}}\cdot\text{["a-KG\{Mitochondria\}"]}+\text{[Malate\{Cytosol\}]}\cdot\text{["a-KG\{Mitochondria\}"]}+0.0000001}\right)$$

$$\frac{d\left(\text{["Ribulose-5P"]}\cdot V_{\text{Cytosol}}\right)}{d\,t} = -V_{\text{Cytosol}}\cdot\left(\text{kcat}_{\text{("(PPP) RPI")}}\cdot\text{[enz\_RPI]}\cdot\frac{\text{["Ribulose-5P"]}}{\text{Km}_{\text{("(PPP) RPI")}}+\text{["Ribulose-5P"]}}\right)$$

$$-V_{\text{Cytosol}}\cdot\left(\text{kcat}_{\text{("(PPP) RPE")}}\cdot\text{[enz\_RPE]}\cdot\frac{\text{["Ribulose-5P"]}}{\text{Km}_{\text{("(PPP) RPE")}}+\text{["Ribulose-5P"]}}\right)$$

$$+V_{\text{Cytosol}}\cdot\left(\text{kcat}_{\text{("(PPP) PGD")}}\cdot\text{[enz\_PGD]}\cdot\frac{\text{["Gluconate-6P"]}}{\text{Km}_{\text{("(PPP) PGD")}}+\text{["Gluconate-6P"]}}\right)$$

$$\frac{d\left(\text{["Gluconate-6P"]}\cdot V_{\text{Cytosol}}\right)}{d\,t} = -V_{\text{Cytosol}}\cdot\left(\text{kcat}_{\text{("(PPP) PGD")}}\cdot\text{[enz\_PGD]}\cdot\frac{\text{["Gluconate-6P"]}}{\text{Km}_{\text{("(PPP) PGD")}}+\text{["Gluconate-6P"]}}\right)$$

$$+V_{\text{Cytosol}}\cdot\left(\text{kcat}_{\text{("(PPP) PGL")}}\cdot\text{[enz\_PGL]}\cdot\frac{\text{[G1,5L6P]}}{\text{Km}_{\text{("(PPP) PGL")}}+\text{[G1,5L6P]}}\right)$$

$$\text{[enz\_PGAM]} = \text{Naive}\cdot 1.54\text{E-}07+\text{Th1}\cdot 2.86\text{E-}07+\text{Th2}\cdot 2.85\text{E-}07+\text{Th17}\cdot 3.34\text{E-}07+\text{Th22}\cdot 3.15\text{E-}07+\text{Treg}\cdot 3.37\text{E-}07$$

$$[\text{enz\_PGD}] = \text{Naive}\cdot 7.65\text{E-}08 + \text{Th1}\cdot 8.06\text{E-}08 + \text{Th2}\cdot 9.18\text{E-}08 + \text{Th17}\cdot 9.14\text{E-}08 + \text{Th22}\cdot 9.21\text{E-}08 + \text{Treg}\cdot 8.00\text{E-}08$$

$$[\text{enz\_ACSL}] = \text{Naive}\cdot 4.21\text{E-}08 + \text{Th1}\cdot 4.84\text{E-}08 + \text{Th2}\cdot 4.14\text{E-}08 + \text{Th17}\cdot 3.91\text{E-}08 + \text{Th22}\cdot 5.00\text{E-}08 + \text{Treg}\cdot 7.12\text{E-}08$$

$$[\text{NADH\_S\_OXPHOS}] = \begin{cases} [\text{NADH\_P\_IDH}] + [\text{NADH\_P\_MDH}] + [\text{NADH\_P\_OGDH}] + [\text{NADH\_P\_PDH}] - [\text{NAD\_P\_OXPHOS}] > 0.0, & [\text{NADH\_P\_IDH}] + [\text{NADH\_P\_MDH}] + [\text{NADH\_P\_OGDH}] + [\text{NADH\_P\_PDH}] - [\text{NAD\_P\_OXPHOS}] \\ \text{else}, & 0.0 \end{cases}$$

$$\frac{d\left([\text{NADH\_P\_IDH}]\cdot V_{\text{Mitochondria}}\right)}{d\,t} = +V_{\text{Mitochondria}}\cdot\left(\text{kcat}_{(\text{"(TCA) IDH"})}\cdot[\text{enz\_IDH}]\cdot\frac{[\text{Isocitrate}]\cdot[\text{NAD\_S\{Mitochondria\}}]}{\text{KmB}_{(\text{"(TCA) IDH"})}\cdot[\text{Isocitrate}] + \text{KmA}_{(\text{"(TCA) IDH"})}\cdot[\text{NAD\_S\{Mitochondria\}}] + [\text{Isocitrate}]\cdot[\text{NAD\_S\{Mitochondria\}}] + 0.0000001}\right)$$

$$\frac{d\left([\text{NADH\_P\_MDH}]\cdot V_{\text{Mitochondria}}\right)}{d\,t} = +V_{\text{Mitochondria}}\cdot\left(\text{kcat}_{(\text{"(TCA) MDH2"})}\cdot[\text{enz\_MDH2}]\cdot\frac{[\text{Malate\{Mitochondria\}}]\cdot[\text{NAD\_S\{Mitochondria\}}]}{\text{KmB}_{(\text{"(TCA) MDH2"})}\cdot[\text{Malate\{Mitochondria\}}] + \text{KmA}_{(\text{"(TCA) MDH2"})}\cdot[\text{NAD\_S\{Mitochondria\}}] + [\text{Malate\{Mitochondria\}}]\cdot[\text{NAD\_S\{Mitochondria\}}] + 0.0000001}\right)$$

$$\frac{d\left([\text{NADH\_P\_OGDH}]\cdot V_{\text{Mitochondria}}\right)}{d\,t} = +V_{\text{Cytosol}}\cdot\left(\text{kcat}_{(\text{"(TCA) OGDH"})}\cdot[\text{enz\_OGDH}]\cdot\frac{[\text{"a-KG\{Mitochondria\}"}]\cdot[\text{NAD\_S\{Mitochondria\}}]}{\text{KmB}_{(\text{"(TCA) OGDH"})}\cdot[\text{"a-KG\{Mitochondria\}"}] + \text{KmA}_{(\text{"(TCA) OGDH"})}\cdot[\text{NAD\_S\{Mitochondria\}}] + [\text{"a-KG\{Mitochondria\}"}]\cdot[\text{NAD\_S\{Mitochondria\}}] + 0.0000001}\right)$$

$$\frac{d\left([\text{NADH\_P\_PDH}]\cdot V_{\text{Mitochondria}}\right)}{d\,t} = +V_{\text{Mitochondria}}\cdot\left(\text{kcat}_{(\text{"(TCA) PDH"})}\cdot[\text{enz\_PDH}]\cdot\frac{[\text{Pyruvate\{Mitochondria\}}]}{\text{Km}_{(\text{"(TCA) PDH"})} + [\text{Pyruvate\{Mitochondria\}}]}\cdot\left(1 + \frac{\text{c1}_{(\text{"(TCA) PDH"})}\cdot[\text{"LANCL2-ABA"}]^{\text{n1}_{(\text{"(TCA) PDH"})}}}{[\text{"LANCL2-ABA"}]^{\text{n1}_{(\text{"(TCA) PDH"})}} + \text{ka}_{(\text{"(TCA) PDH"})}{}^{\text{n1}_{(\text{"(TCA) PDH"})}}}\right)\right)$$

$$\frac{d\left([\text{NAD\_P\_OXPHOS}]\cdot V_{\text{Cytosol}}\right)}{d\,t} = +V_{\text{Mitochondria}}\cdot\left(\text{kcat}_{(\text{"(OXPHOS) I-IV"})}\cdot[\text{enz\_ComplexI}]\cdot\frac{[\text{NADH\_S\_OXPHOS}]}{\text{Km}_{(\text{"(OXPHOS) I-IV"})} + [\text{NADH\_S\_OXPHOS}]}\cdot\frac{[\text{H\{Mitochondria\}}]}{\text{Km2}_{(\text{"(OXPHOS) I-IV"})} + [\text{H\{Mitochondria\}}]}\right)$$

$$\frac{d\left([\text{"Succinyl-CoA"}]\cdot V_{\text{Mitochondria}}\right)}{d\,t} = +V_{\text{Cytosol}}\cdot\left(\text{kcat}_{(\text{"(TCA) OGDH"})}\cdot[\text{enz\_OGDH}]\cdot\frac{[\text{"a-KG\{Mitochondria\}"}]\cdot[\text{NAD\_S\{Mitochondria\}}]}{\text{KmB}_{(\text{"(TCA) OGDH"})}\cdot[\text{"a-KG\{Mitochondria\}"}] + \text{KmA}_{(\text{"(TCA) OGDH"})}\cdot[\text{NAD\_S\{Mitochondria\}}] + [\text{"a-KG\{Mitochondria\}"}]\cdot[\text{NAD\_S\{Mitochondria\}}] + 0.0000001}\right)$$
$$-V_{\text{Mitochondria}}\cdot\left(\text{kcat}_{(\text{"(TCA) SUCL\_F"})}\cdot[\text{enz\_SUCL}]\cdot\frac{[\text{"Succinyl-CoA"}]}{\text{Km}_{(\text{"(TCA) SUCL\_F"})} + [\text{"Succinyl-CoA"}]}\right)$$
$$+V_{\text{Mitochondria}}\cdot\left(\text{kcat}_{(\text{"(TCA) SUCL\_R"})}\cdot[\text{enz\_SUCL}]\cdot\frac{[\text{Succinate}]}{\text{Km}_{(\text{"(TCA) SUCL\_R"})} + [\text{Succinate}]}\right)$$

$$
\begin{aligned}
\frac{d\left([\text{OAA}\{\text{Mitochondria}\}]\cdot V_{\text{Mitochondria}}\right)}{d\,t} = &+V_{\text{Mitochondria}}\cdot\left(\text{kcat}_{(\text{"(TCA) PC-A"})}\cdot[\text{"enz\_PC-A"}]\cdot\frac{[\text{Pyruvate}\{\text{Mitochondria}\}]}{\text{Km}_{(\text{"(TCA) PC-A"})}+[\text{Pyruvate}\{\text{Mitochondria}\}]}\right)\\
&-V_{\text{Mitochondria}}\cdot\left(\text{kcat}_{(\text{"(GLU) PEPCK\_M"})}\cdot[\text{enz\_PCK2}]\cdot\frac{[\text{OAA}\{\text{Mitochondria}\}]}{\text{Km}_{(\text{"(GLU) PEPCK\_M"})}+[\text{OAA}\{\text{Mitochondria}\}]}\right)\\
&+V_{\text{Mitochondria}}\cdot\left(\text{kcat}_{(\text{"(TCA) MDH2"})}\cdot[\text{enz\_MDH2}]\cdot\frac{[\text{Malate}\{\text{Mitochondria}\}]\cdot[\text{NAD\_S}\{\text{Mitochondria}\}]}{\text{KmB}_{(\text{"(TCA) MDH2"})}\cdot[\text{Malate}\{\text{Mitochondria}\}]+\text{KmA}_{(\text{"(TCA) MDH2"})}\cdot[\text{NAD\_S}\{\text{Mitochondria}\}]+[\text{Malate}\{\text{Mitochondria}\}]\cdot[\text{NAD\_S}\{\text{Mitochondria}\}]+0.0000001}\right)\\
&-V_{\text{Mitochondria}}\cdot\left(\text{kcat}_{(\text{"(TCA) CS"})}\cdot[\text{enz\_CS}]\cdot\frac{[\text{OAA}\{\text{Mitochondria}\}]\cdot[\text{"Acetyl-CoA}\{\text{Mitochondria}\}\text{"}]}{\text{KmB}_{(\text{"(TCA) CS"})}\cdot[\text{OAA}\{\text{Mitochondria}\}]+\text{KmA}_{(\text{"(TCA) CS"})}\cdot[\text{"Acetyl-CoA}\{\text{Mitochondria}\}\text{"}]+[\text{OAA}\{\text{Mitochondria}\}]\cdot[\text{"Acetyl-CoA}\{\text{Mitochondria}\}\text{"}]+0.0000001}\right)\\
&-V_{\text{Mitochondria}}\cdot\left(\text{kcat}_{(\text{"(MAS) GOT2"})}\cdot[\text{enz\_GOT2}]\cdot\frac{[\text{OAA}\{\text{Mitochondria}\}]\cdot[\text{Glutamate}\{\text{Mitochondria}\}]}{\text{KmB}_{(\text{"(MAS) GOT2"})}\cdot[\text{OAA}\{\text{Mitochondria}\}]+\text{KmA}_{(\text{"(MAS) GOT2"})}\cdot[\text{Glutamate}\{\text{Mitochondria}\}]+[\text{OAA}\{\text{Mitochondria}\}]\cdot[\text{Glutamate}\{\text{Mitochondria}\}]+0.0000001}\right)\\
&+V_{\text{Mitochondria}}\cdot\left(\text{kcat}_{(\text{"(TCA) PC"})}\cdot[\text{enz\_PC}]\cdot\frac{[\text{Pyruvate}\{\text{Mitochondria}\}]}{\text{Km}_{(\text{"(TCA) PC"})}+[\text{Pyruvate}\{\text{Mitochondria}\}]}\right)
\end{aligned}
$$

$$
\begin{aligned}
\frac{d\left([\text{Succinate}]\cdot V_{\text{Mitochondria}}\right)}{d\,t} = &-V_{\text{Cytosol}}\cdot\left(\text{kcat}_{(\text{"(TCA) SDH\_F"})}\cdot[\text{enz\_SDH}]\cdot\frac{[\text{Succinate}]}{\text{Km}_{(\text{"(TCA) SDH\_F"})}+[\text{Succinate}]}\right)\\
&+V_{\text{Mitochondria}}\cdot\left(\text{kcat}_{(\text{"(TCA) SUCL\_F"})}\cdot[\text{enz\_SUCL}]\cdot\frac{[\text{"Succinyl-CoA"}]}{\text{Km}_{(\text{"(TCA) SUCL\_F"})}+[\text{"Succinyl-CoA"}]}\right)\\
&-V_{\text{Mitochondria}}\cdot\left(\text{kcat}_{(\text{"(TCA) SUCL\_R"})}\cdot[\text{enz\_SUCL}]\cdot\frac{[\text{Succinate}]}{\text{Km}_{(\text{"(TCA) SUCL\_R"})}+[\text{Succinate}]}\right)\\
&+V_{\text{Cytosol}}\cdot\left(\text{kcat}_{(\text{"(TCA) SDH\_R"})}\cdot[\text{enz\_SDH}]\cdot\frac{[\text{Fumarate}]}{\text{Km}_{(\text{"(TCA) SDH\_R"})}+[\text{Fumarate}]}\right)
\end{aligned}
$$

$$\frac{d\left([\text{Isocitrate}]\cdot V_{\text{Mitochondria}}\right)}{d\,t} = -V_{\text{Mitochondria}}\cdot\left(\text{kcat}_{(\text{"(TCA) IDH"})}\cdot[\text{enz\_IDH}]\cdot\frac{[\text{Isocitrate}]\cdot[\text{NAD\_S\{Mitochondria\}}]}{\text{KmB}_{(\text{"(TCA) IDH"})}\cdot[\text{Isocitrate}]+\text{KmA}_{(\text{"(TCA) IDH"})}\cdot[\text{NAD\_S\{Mitochondria\}}]+[\text{Isocitrate}]\cdot[\text{NAD\_S\{Mitochondria\}}]+0.0000001}\right)$$

$$+V_{\text{Mitochondria}}\cdot\left(\text{kcat}_{(\text{"(TCA) ACO"})}\cdot[\text{enz\_ACO}]\cdot\frac{[\text{Citrate\{Mitochondria\}}]}{\text{Km}_{(\text{"(TCA) ACO"})}+[\text{Citrate\{Mitochondria\}}]}\right)$$

$$\frac{d\left([\text{Citrate\{Mitochondria\}}]\cdot V_{\text{Mitochondria}}\right)}{d\,t} = -V_{\text{Mitochondria}}\cdot\left(\text{kcat}_{(\text{"(FAS) SLC25A1\_F"})}\cdot[\text{enz\_SLC25A1}]\cdot\frac{[\text{Citrate\{Mitochondria\}}]}{\text{Km}_{(\text{"(FAS) SLC25A1\_F"})}+[\text{Citrate\{Mitochondria\}}]}\right)$$

$$+V_{\text{Mitochondria}}\cdot\left(\text{kcat}_{(\text{"(FAS) SLC25A1\_R"})}\cdot[\text{enz\_SLC25A1}]\cdot\frac{[\text{Citrate\{Cytosol\}}]}{\text{Km}_{(\text{"(FAS) SLC25A1\_R"})}+[\text{Citrate\{Cytosol\}}]}\right)$$

$$+V_{\text{Mitochondria}}\cdot\left(\text{kcat}_{(\text{"(TCA) CS"})}\cdot[\text{enz\_CS}]\cdot\frac{[\text{OAA\{Mitochondria\}}]\cdot[\text{"Acetyl-CoA\{Mitochondria\}"}]}{\text{KmB}_{(\text{"(TCA) CS"})}\cdot[\text{OAA\{Mitochondria\}}]+\text{KmA}_{(\text{"(TCA) CS"})}\cdot[\text{"Acetyl-CoA\{Mitochondria\}"}]+[\text{OAA\{Mitochondria\}}]\cdot[\text{"Acetyl-CoA\{Mitochondria\}"}]+0.0000001}\right)$$

$$-V_{\text{Mitochondria}}\cdot\left(\text{kcat}_{(\text{"(TCA) ACO"})}\cdot[\text{enz\_ACO}]\cdot\frac{[\text{Citrate\{Mitochondria\}}]}{\text{Km}_{(\text{"(TCA) ACO"})}+[\text{Citrate\{Mitochondria\}}]}\right)$$

$$[\text{enz\_G6PC}] = \text{Naive}\cdot 1.00\text{E-}10+\text{Th1}\cdot 1.00\text{E-}10+\text{Th2}\cdot 1.00\text{E-}10+\text{Th17}\cdot 1.00\text{E-}10+\text{Th22}\cdot 1.00\text{E-}10+\text{Treg}\cdot 1.00\text{E-}10$$

$$[\text{enz\_G6PD}] = (\text{Naive}\cdot 9.88\text{E-}08+\text{Th1}\cdot 8.57\text{E-}08+\text{Th2}\cdot 7.52\text{E-}08+\text{Th17}\cdot 8.66\text{E-}08+\text{Th22}\cdot 7.63\text{E-}08+\text{Treg}\cdot 1.15\text{E-}07)\cdot\text{g6pd\_on}+(1-\text{g6pd\_on})\cdot\text{Values[temp].InitialValue}$$

$$\frac{d\left([\text{Pyruvate\{Mitochondria\}}]\cdot V_{\text{Mitochondria}}\right)}{d\,t} = -V_{\text{Mitochondria}}\cdot\left(\text{kcat}_{(\text{"(TCA) PC-A"})}\cdot[\text{"enz\_PC-A"}]\cdot\frac{[\text{Pyruvate\{Mitochondria\}}]}{\text{Km}_{(\text{"(TCA) PC-A"})}+[\text{Pyruvate\{Mitochondria\}}]}\right)$$

$$-V_{\text{Mitochondria}}\cdot\left(\text{kcat}_{(\text{"(TCA) PDH"})}\cdot[\text{enz\_PDH}]\cdot\frac{[\text{Pyruvate\{Mitochondria\}}]}{\text{Km}_{(\text{"(TCA) PDH"})}+[\text{Pyruvate\{Mitochondria\}}]}\cdot\left(1+\frac{\text{c1}_{(\text{"(TCA) PDH"})}\cdot[\text{"LANCL2-ABA"}]^{\text{n1}_{(\text{"(TCA) PDH"})}}}{[\text{"LANCL2-ABA"}]^{\text{n1}_{(\text{"(TCA) PDH"})}}+\text{ka}_{(\text{"(TCA) PDH"})}^{\text{n1}_{(\text{"(TCA) PDH"})}}}\right)\right)$$

$$+V_{\text{Cytosol}}\cdot\left(\text{kcat}_{(\text{"(Mito Transport) MPC\_F"})}\cdot[\text{enz\_MPC}]\cdot\frac{[\text{Pyruvate\{Cytosol\}}]}{\text{Km}_{(\text{"(Mito Transport) MPC\_F"})}+[\text{Pyruvate\{Cytosol\}}]}\right)$$

$$+V_{\text{Mitochondria}}\cdot\left(\text{kcat}_{(\text{"(TCA) ME"})}\cdot[\text{enz\_ME2}]\cdot\frac{[\text{Malate}\{\text{Mitochondria}\}]}{\text{Km}_{(\text{"(TCA) ME"})}+[\text{Malate}\{\text{Mitochondria}\}]}\right)$$

$$-V_{\text{Mitochondria}}\cdot\left(\text{kcat}_{(\text{"(TCA) PC"})}\cdot[\text{enz\_PC}]\cdot\frac{[\text{Pyruvate}\{\text{Mitochondria}\}]}{\text{Km}_{(\text{"(TCA) PC"})}+[\text{Pyruvate}\{\text{Mitochondria}\}]}\right)$$

$$\frac{d\left([\text{NADPH\_P\_PGD}]\cdot V_{\text{Cytosol}}\right)}{d\,t}=-V_{\text{Cytosol}}\cdot\left(\text{k1}_{(\text{"(IM) NADPH\_P6D\_deg"})}\cdot[\text{NADPH\_P\_PGD}]\right)$$

$$+V_{\text{Cytosol}}\cdot\left(\text{kcat}_{(\text{"(PPP) PGD"})}\cdot[\text{enz\_PGD}]\cdot\frac{[\text{"Gluconate-6P"}]}{\text{Km}_{(\text{"(PPP) PGD"})}+[\text{"Gluconate-6P"}]}\right)$$

$$\frac{d\left([\text{NADH\_P\_LDHB}]\cdot V_{\text{Cytosol}}\right)}{d\,t}=+V_{\text{Cytosol}}\cdot\left(\text{kcat}_{(\text{"(GLU) LDHB"})}\cdot[\text{enz\_LDHB}]\cdot\frac{[\text{Lactate}\{\text{Cytosol}\}]\cdot[\text{NAD\_S}\{\text{Cytosol}\}]}{\text{KmB}_{(\text{"(GLU) LDHB"})}\cdot[\text{Lactate}\{\text{Cytosol}\}]+\text{KmA}_{(\text{"(GLU) LDHB"})}\cdot[\text{NAD\_S}\{\text{Cytosol}\}]+[\text{Lactate}\{\text{Cytosol}\}]\cdot[\text{NAD\_S}\{\text{Cytosol}\}]+0.0000001}\right)$$

$$\frac{d\left([\text{NADH\_P\_GAPDH}]\cdot V_{\text{Cytosol}}\right)}{d\,t}=+V_{\text{Cytosol}}\cdot\left(\text{kcat}_{(\text{"(GLY) GAPDH\_F"})}\cdot[\text{enz\_GAPDH}]\cdot\frac{[\text{"G3-P"}]\cdot[\text{NAD\_S}\{\text{Cytosol}\}]}{\text{KmB}_{(\text{"(GLY) GAPDH\_F"})}\cdot[\text{"G3-P"}]+\text{KmA}_{(\text{"(GLY) GAPDH\_F"})}\cdot[\text{NAD\_S}\{\text{Cytosol}\}]+[\text{"G3-P"}]\cdot[\text{NAD\_S}\{\text{Cytosol}\}]+0.0000001}\right)$$

$$\frac{d\left([\text{FAD\_P\_OXPHOS}]\cdot V_{\text{Mitochondria}}\right)}{d\,t}=+V_{\text{Mitochondria}}\cdot\left(\text{kcat}_{(\text{"(OXPHOS) II-IV"})}\cdot[\text{enz\_ComplexII}]\cdot\frac{[\text{FADH2\_S\_OXPHOS}]}{\text{Km}_{(\text{"(OXPHOS) II-IV"})}+[\text{FADH2\_S\_OXPHOS}]}\cdot\frac{[\text{H}\{\text{Mitochondria}\}]}{\text{Km2}_{(\text{"(OXPHOS) II-IV"})}+[\text{H}\{\text{Mitochondria}\}]}\right)$$

$$\frac{d\left([\text{ADP\_P\_PFK}]\cdot V_{\text{Cytosol}}\right)}{d\,t}=+V_{\text{Cytosol}}\cdot\left(\text{kcat}_{(\text{"(GLY) PFK"})}\cdot[\text{enz\_PFK}]\cdot\frac{[\text{"F6-P"}]}{\text{Km}_{(\text{"(GLY) PFK"})}+[\text{"F6-P"}]}\right)$$

$$\frac{d\left([\text{NADPH\_P\_G6PD}]\cdot V_{\text{Cytosol}}\right)}{d\,t}=-V_{\text{Cytosol}}\cdot\left(\text{k1}_{(\text{"(IM) NADPH\_G6PD\_deg"})}\cdot[\text{NADPH\_P\_G6PD}]\right)$$

$$+V_{\text{Cytosol}}\cdot\left(\text{kcat}_{(\text{"(PPP) G6PD"})}\cdot[\text{enz\_G6PD}]\cdot\frac{[\text{"G6-P"}]}{\text{Km}_{(\text{"(PPP) G6PD"})}+[\text{"G6-P"}]}\right)$$

$$[\text{enz\_LDHB}]=\text{Naive}\cdot 3.57\text{E-}06+\text{Th1}\cdot 2.80\text{E-}06+\text{Th2}\cdot 3.44\text{E-}06+\text{Th17}\cdot 3.58\text{E-}06+\text{Th22}\cdot 2.46\text{E-}06+\text{Treg}\cdot 3.32\text{E-}06$$

$$\frac{d\left([\text{ADP\_P\_HK}] \cdot V_{\text{Cytosol}}\right)}{d\,t} = +V_{\text{Cytosol}} \cdot \left(\text{kcat}_{(\text{"(GLY) HK"})} \cdot [\text{enz\_HK}] \cdot \frac{[\text{Glucose\{Cytosol\}}]}{\text{Km}_{(\text{"(GLY) HK"})} + [\text{Glucose\{Cytosol\}}]}\right)$$

$$\frac{d\left([\text{NAD\_P\_LDHA}] \cdot V_{\text{Cytosol}}\right)}{d\,t} = +V_{\text{Cytosol}} \cdot \left(\text{kcat}_{(\text{"(GLY) LDHA"})} \cdot [\text{enz\_LDHA}] \cdot \frac{[\text{Pyruvate\{Cytosol\}}]}{\text{Km}_{(\text{"(GLY) LDHA"})} + [\text{Pyruvate\{Cytosol\}}]}\right)$$

$$[\text{FADH2\_S\_OXPHOS}] = \begin{cases} [\text{FADH2\_P\_SDH}] - [\text{FAD\_P\_OXPHOS}] > 0.0, & [\text{FADH2\_P\_SDH}] - [\text{FAD\_P\_OXPHOS}] \\ \text{else}, & 0.0 \end{cases}$$

$$\frac{d\left([\text{FADH2\_P\_SDH}] \cdot V_{\text{Mitochondria}}\right)}{d\,t} = +V_{\text{Cytosol}} \cdot \left(\text{kcat}_{(\text{"(TCA) SDH\_F"})} \cdot [\text{enz\_SDH}] \cdot \frac{[\text{Succinate}]}{\text{Km}_{(\text{"(TCA) SDH\_F"})} + [\text{Succinate}]}\right)$$

$$\frac{d\left([\text{ATP\_P\_PGK}] \cdot V_{\text{Cytosol}}\right)}{d\,t} = +V_{\text{Cytosol}} \cdot \left(\text{kcat}_{(\text{"(GLY) PGK\_F"})} \cdot [\text{enz\_PGK}] \cdot \frac{[\text{"1,3-BPG"}]}{\text{Km}_{(\text{"(GLY) PGK\_F"})} + [\text{"1,3-BPG"}]}\right)$$

$$\frac{d\left([\text{ATP\_P\_PK}] \cdot V_{\text{Cytosol}}\right)}{d\,t} = +V_{\text{Cytosol}} \cdot \left(\text{kcat}_{(\text{"(GLY) PK"})} \cdot [\text{enz\_PK}] \cdot \frac{[\text{PEP\{Cytosol\}}]}{\text{Km}_{(\text{"(GLY) PK"})} + [\text{PEP\{Cytosol\}}]} \cdot \left(1 + \frac{\text{c1}_{(\text{"(GLY) PK"})} \cdot [\text{"LANCL2-ABA"}]^{\text{n1}_{(\text{"(GLY) PK"})}}}{[\text{"LANCL2-ABA"}]^{\text{n1}_{(\text{"(GLY) PK"})}} + \text{ka}_{(\text{"(GLY) PK"})}^{\text{n1}_{(\text{"(GLY) PK"})}}}\right)\right)$$

$$\frac{d\left([\text{ATP\_P\_SUCL}] \cdot V_{\text{Mitochondria}}\right)}{d\,t} = +V_{\text{Mitochondria}} \cdot \left(\text{kcat}_{(\text{"(TCA) SUCL\_F"})} \cdot [\text{enz\_SUCL}] \cdot \frac{[\text{"Succinyl-CoA"}]}{\text{Km}_{(\text{"(TCA) SUCL\_F"})} + [\text{"Succinyl-CoA"}]}\right)$$

$$\begin{aligned} \frac{d\left([\text{Glucose\{Cytosol\}}] \cdot V_{\text{Cytosol}}\right)}{d\,t} = & +V_{\text{Cytosol}} \cdot \left(\text{kcat}_{(\text{"(GLY) GLUT"})} \cdot [\text{enz\_GLUT}] \cdot \frac{[\text{Glucose\{Extracellular\}}]}{\text{Km}_{(\text{"(GLY) GLUT"})} + [\text{Glucose\{Extracellular\}}]}\right) \\ & -V_{\text{Cytosol}} \cdot \left(\text{kcat}_{(\text{"(GLY) HK"})} \cdot [\text{enz\_HK}] \cdot \frac{[\text{Glucose\{Cytosol\}}]}{\text{Km}_{(\text{"(GLY) HK"})} + [\text{Glucose\{Cytosol\}}]}\right) \\ & +V_{\text{Cytosol}} \cdot \left(\text{kcat}_{(\text{"(GLU) G6PC"})} \cdot [\text{enz\_G6PC}] \cdot \frac{[\text{"G6-P"}]}{\text{Km}_{(\text{"(GLU) G6PC"})} + [\text{"G6-P"}]}\right) \end{aligned}$$

$$- V_{\text{Cytosol}} \cdot \left(\text{kcat}_{(\text{"(GLY) GLUT\_R"})} \cdot [\text{enz\_GLUT}] \cdot \frac{[\text{Glucose\{Cytosol\}}]}{\text{Km}_{(\text{"(GLY) GLUT\_R"})} + [\text{Glucose\{Cytosol\}}]} \right)$$

$$\frac{d \left([\text{"F1,6-BP"}] \cdot V_{\text{Cytosol}} \right)}{d\, t} = - V_{\text{Cytosol}} \cdot \left(\text{kcat}_{(\text{"(GLY) ALDO\_F"})} \cdot [\text{enz\_ALDO}] \cdot \frac{[\text{"F1,6-BP"}]}{\text{Km}_{(\text{"(GLY) ALDO\_F"})} + [\text{"F1,6-BP"}]} \right)$$

$$+ V_{\text{Cytosol}} \cdot \left(\text{kcat}_{(\text{"(GLY) PFK"})} \cdot [\text{enz\_PFK}] \cdot \frac{[\text{"F6-P"}]}{\text{Km}_{(\text{"(GLY) PFK"})} + [\text{"F6-P"}]} \right)$$

$$+ V_{\text{Cytosol}} \cdot \left(\text{kcat}_{(\text{"(GLY) ALDO\_R"})} \cdot [\text{enz\_ALDO}] \cdot \frac{[\text{"G3-P"}]}{\text{Km}_{(\text{"(GLY) ALDO\_R"})} + [\text{"G3-P"}]} \right)$$

$$- V_{\text{Cytosol}} \cdot \left(\text{kcat}_{(\text{"(GLU) FBP"})} \cdot [\text{enz\_FBP}] \cdot \frac{[\text{"F1,6-BP"}]}{\text{Km}_{(\text{"(GLU) FBP"})} + [\text{"F1,6-BP"}]} \right)$$

$$\frac{d \left([\text{"G6-P"}] \cdot V_{\text{Cytosol}} \right)}{d\, t} = - V_{\text{Cytosol}} \cdot \left(\text{kcat}_{(\text{"(GLY) GPI\_F"})} \cdot [\text{enz\_GPI}] \cdot \frac{[\text{"G6-P"}]}{\text{Km}_{(\text{"(GLY) GPI\_F"})} + [\text{"G6-P"}]} \right)$$

$$+ V_{\text{Cytosol}} \cdot \left(\text{kcat}_{(\text{"(GLY) HK"})} \cdot [\text{enz\_HK}] \cdot \frac{[\text{Glucose\{Cytosol\}}]}{\text{Km}_{(\text{"(GLY) HK"})} + [\text{Glucose\{Cytosol\}}]} \right)$$

$$- V_{\text{Cytosol}} \cdot \left(\text{kcat}_{(\text{"(PPP) G6PD"})} \cdot [\text{enz\_G6PD}] \cdot \frac{[\text{"G6-P"}]}{\text{Km}_{(\text{"(PPP) G6PD"})} + [\text{"G6-P"}]} \right)$$

$$- V_{\text{Cytosol}} \cdot \left(\text{kcat}_{(\text{"(GLU) G6PC"})} \cdot [\text{enz\_G6PC}] \cdot \frac{[\text{"G6-P"}]}{\text{Km}_{(\text{"(GLU) G6PC"})} + [\text{"G6-P"}]} \right)$$

$$+ V_{\text{Cytosol}} \cdot \left(\text{kcat}_{(\text{"(GLY) GPI\_R"})} \cdot [\text{enz\_GPI}] \cdot \frac{[\text{"F6-P"}]}{\text{Km}_{(\text{"(GLY) GPI\_R"})} + [\text{"F6-P"}]} \right)$$

$$\begin{aligned}
\frac{d\left(["2\text{-}PG"]\cdot V_{Cytosol}\right)}{dt} = & -V_{Cytosol}\cdot\left(kcat_{("(GLY)\ ENO_F")}\cdot[enz_ENO1]\cdot\frac{["2\text{-}PG"]}{Km_{("(GLY)\ ENO_F")}+["2\text{-}PG"]}\right)\\
& +V_{Cytosol}\cdot\left(kcat_{("(GLY)\ PGAM_F")}\cdot[enz_PGAM]\cdot\frac{["3\text{-}PG"]}{Km_{("(GLY)\ PGAM_F")}+["3\text{-}PG"]}\right)\\
& -V_{Cytosol}\cdot\left(kcat_{("(GLY)\ PGAM_R")}\cdot[enz_PGAM]\cdot\frac{["2\text{-}PG"]}{Km_{("(GLY)\ PGAM_R")}+["2\text{-}PG"]}\right)\\
& +V_{Cytosol}\cdot\left(kcat_{("(GLY)\ ENO_R")}\cdot[enz_ENO1]\cdot\frac{[PEP\{Cytosol\}]}{Km_{("(GLY)\ ENO_R")}+[PEP\{Cytosol\}]}\right)
\end{aligned}$$

$$\begin{aligned}
\frac{d\left([Pyruvate\{Cytosol\}]\cdot V_{Cytosol}\right)}{dt} = & +V_{Cytosol}\cdot\left(kcat_{("(GLY)\ PK")}\cdot[enz_PK]\cdot\frac{[PEP\{Cytosol\}]}{Km_{("(GLY)\ PK")}+[PEP\{Cytosol\}]}\cdot\left(1+\frac{c1_{("(GLY)\ PK")}\cdot["LANCL2\text{-}ABA"]^{n1_{("(GLY)\ PK")}}}{["LANCL2\text{-}ABA"]^{n1_{("(GLY)\ PK")}}+ka_{("(GLY)\ PK")}^{\ n1_{("(GLY)\ PK")}}}\right)\right)\\
& -V_{Cytosol}\cdot\left(kcat_{("(GLY)\ LDHA")}\cdot[enz_LDHA]\cdot\frac{[Pyruvate\{Cytosol\}]}{Km_{("(GLY)\ LDHA")}+[Pyruvate\{Cytosol\}]}\right)\\
& +V_{Cytosol}\cdot\left(kcat_{("(GLU)\ LDHB")}\cdot[enz_LDHB]\cdot\frac{[Lactate\{Cytosol\}]\cdot[NAD_S\{Cytosol\}]}{KmB_{("(GLU)\ LDHB")}\cdot[Lactate\{Cytosol\}]+KmA_{("(GLU)\ LDHB")}\cdot[NAD_S\{Cytosol\}]+[Lactate\{Cytosol\}]\cdot[NAD_S\{Cytosol\}]+0.0000001}\right)\\
& -V_{Cytosol}\cdot\left(kcat_{("(Mito\ Transport)\ MPC_F")}\cdot[enz_MPC]\cdot\frac{[Pyruvate\{Cytosol\}]}{Km_{("(Mito\ Transport)\ MPC_F")}+[Pyruvate\{Cytosol\}]}\right)\\
& +V_{Mitochondria}\cdot\left(kcat_{("(Glutaminolysis)\ ME")}\cdot[enz_ME1]\cdot\frac{[Malate\{Cytosol\}]}{Km_{("(Glutaminolysis)\ ME")}+[Malate\{Cytosol\}]}\right)
\end{aligned}$$

$$\frac{d\left(["Trans\text{-}enoyl\text{-}CoA"]\cdot V_{Mitochondria}\right)}{dt} = -V_{Mitochondria}\cdot\left(kcat_{("(FAO)\ ECH")}\cdot[enz\_ECH]\cdot\frac{["Trans\text{-}enoyl\text{-}CoA"]}{Km_{("(FAO)\ ECH")}+["Trans\text{-}enoyl\text{-}CoA"]}\right)$$

$$+V_{\text{Mitochondria}}\cdot\left(\text{kcat}_{\text{"(FAO) ACAD"}}\cdot[\text{enz\_ACAD}]\cdot\frac{[\text{"Acyl-CoA\{Mitochondria\}"}]}{\text{Km}_{\text{"(FAO) ACAD"}}+[\text{"Acyl-CoA\{Mitochondria\}"}]}\right)$$

$$\frac{d\left([\text{"1,3-BPG"}]\cdot V_{\text{Cytosol}}\right)}{d\,t} = +V_{\text{Cytosol}}\cdot\left(\text{kcat}_{\text{"(GLY) GAPDH\_F"}}\cdot[\text{enz\_GAPDH}]\cdot\frac{[\text{"G3-P"}]\cdot[\text{NAD\_S\{Cytosol\}}]}{\text{KmB}_{\text{"(GLY) GAPDH\_F"}}\cdot[\text{"G3-P"}]+\text{KmA}_{\text{"(GLY) GAPDH\_F"}}\cdot[\text{NAD\_S\{Cytosol\}}]+[\text{"G3-P"}]\cdot[\text{NAD\_S\{Cytosol\}}]+0.0000001}\right)$$

$$-V_{\text{Cytosol}}\cdot\left(\text{kcat}_{\text{"(GLY) PGK\_F"}}\cdot[\text{enz\_PGK}]\cdot\frac{[\text{"1,3-BPG"}]}{\text{Km}_{\text{"(GLY) PGK\_F"}}+[\text{"1,3-BPG"}]}\right)$$

$$-V_{\text{Cytosol}}\cdot\left(\text{kcat}_{\text{"(GLY) GAPDH\_R"}}\cdot[\text{enz\_GAPDH}]\cdot\frac{[\text{"1,3-BPG"}]}{\text{Km}_{\text{"(GLY) GAPDH\_R"}}+[\text{"1,3-BPG"}]}\right)$$

$$+V_{\text{Cytosol}}\cdot\left(\text{kcat}_{\text{"(GLY) PGK\_R"}}\cdot[\text{enz\_PGK}]\cdot\frac{[\text{"3-PG"}]}{\text{Km}_{\text{"(GLY) PGK\_R"}}+[\text{"3-PG"}]}\right)$$

$$\frac{d\left([\text{"3-PG"}]\cdot V_{\text{Cytosol}}\right)}{d\,t} = +V_{\text{Cytosol}}\cdot\left(\text{kcat}_{\text{"(GLY) PGK\_F"}}\cdot[\text{enz\_PGK}]\cdot\frac{[\text{"1,3-BPG"}]}{\text{Km}_{\text{"(GLY) PGK\_F"}}+[\text{"1,3-BPG"}]}\right)$$

$$-V_{\text{Cytosol}}\cdot\left(\text{kcat}_{\text{"(GLY) PGAM\_F"}}\cdot[\text{enz\_PGAM}]\cdot\frac{[\text{"3-PG"}]}{\text{Km}_{\text{"(GLY) PGAM\_F"}}+[\text{"3-PG"}]}\right)$$

$$-V_{\text{Cytosol}}\cdot\left(\text{kcat}_{\text{"(GLY) PGK\_R"}}\cdot[\text{enz\_PGK}]\cdot\frac{[\text{"3-PG"}]}{\text{Km}_{\text{"(GLY) PGK\_R"}}+[\text{"3-PG"}]}\right)$$

$$+V_{\text{Cytosol}}\cdot\left(\text{kcat}_{\text{"(GLY) PGAM\_R"}}\cdot[\text{enz\_PGAM}]\cdot\frac{[\text{"2-PG"}]}{\text{Km}_{\text{"(GLY) PGAM\_R"}}+[\text{"2-PG"}]}\right)$$

$$\frac{d\left([\text{"Acetoacetyl-CoA"}]\cdot V_{\text{Mitochondria}}\right)}{d\,t} = -V_{\text{Mitochondria}}\cdot\left(\text{kcat}_{\text{"(FAO) ACAA"}}\cdot[\text{enz\_ACAA}]\cdot\frac{[\text{"Acetoacetyl-CoA"}]}{\text{Km}_{\text{"(FAO) ACAA"}}+[\text{"Acetoacetyl-CoA"}]}\right)$$

$$+V_{\text{Mitochondria}}\cdot\left(\text{kcat}_{(\text{"(FAO) HADH"})}\cdot[\text{enz\_HADH}]\cdot\frac{[\text{"L-3-Hydroxyacyl-CoA"}]}{\text{Km}_{(\text{"(FAO) HADH"})}+[\text{"L-3-Hydroxyacyl-CoA"}]}\right)$$

$$\frac{d\left([\text{FA\{Cytosol\}}]\cdot V_{\text{Cytosol}}\right)}{d\,t} = -V_{\text{Cytosol}}\cdot\left(\text{kcat}_{(\text{"(FAO) ACSL"})}\cdot[\text{enz\_ACSL}]\cdot\frac{[\text{FA\{Cytosol\}}]}{\text{Km}_{(\text{"(FAO) ACSL"})}+[\text{FA\{Cytosol\}}]}\right)$$

$$+V_{\text{Cytosol}}\cdot\left(\text{kcat}_{(\text{"(FAO) FATP"})}\cdot[\text{enz\_SLC27A1}]\cdot\frac{[\text{FA\{Extracellular\}}]}{\text{Km}_{(\text{"(FAO) FATP"})}+[\text{FA\{Extracellular\}}]}\right)$$

$$-V_{\text{Cytosol}}\cdot\left(\text{kcat}_{(\text{"(FAO) FATP\_R"})}\cdot[\text{enz\_SLC27A1}]\cdot\frac{[\text{FA\{Cytosol\}}]}{\text{Km}_{(\text{"(FAO) FATP\_R"})}+[\text{FA\{Cytosol\}}]}\right)$$

$$\frac{d\left([\text{"Acetyl-CoA\{Mitochondria\}"}]\cdot V_{\text{Mitochondria}}\right)}{d\,t} = -V_{\text{Mitochondria}}\cdot\left(\text{k1}_{(\text{"(AL) PDK activation"})}\cdot[\text{"Acetyl-CoA\{Mitochondria\}"}]\cdot[\text{enz\_PDK}]-\text{k2}_{(\text{"(AL) PDK activation"})}\cdot[\text{"enz\_PDK-A"}]\right)$$

$$-V_{\text{Mitochondria}}\cdot\left(\text{k1}_{(\text{"(AL) PC activation"})}\cdot[\text{"Acetyl-CoA\{Mitochondria\}"}]\cdot[\text{enz\_PC}]-\text{k2}_{(\text{"(AL) PC activation"})}\cdot[\text{"enz\_PC-A"}]\right)$$

$$+V_{\text{Mitochondria}}\cdot\left(\text{kcat}_{(\text{"(FAO) ACAA"})}\cdot[\text{enz\_ACAA}]\cdot\frac{[\text{"Acetoacetyl-CoA"}]}{\text{Km}_{(\text{"(FAO) ACAA"})}+[\text{"Acetoacetyl-CoA"}]}\right)$$

$$+V_{\text{Mitochondria}}\cdot\left(\text{kcat}_{(\text{"(TCA) PDH"})}\cdot[\text{enz\_PDH}]\cdot\frac{[\text{Pyruvate\{Mitochondria\}}]}{\text{Km}_{(\text{"(TCA) PDH"})}+[\text{Pyruvate\{Mitochondria\}}]}\cdot\left(1+\frac{\text{c1}_{(\text{"(TCA) PDH"})}\cdot[\text{"LANCL2-ABA"}]^{\text{n1}_{(\text{"(TCA) PDH"})}}}{[\text{"LANCL2-ABA"}]^{\text{n1}_{(\text{"(TCA) PDH"})}}+\text{ka}_{(\text{"(TCA) PDH"})}^{\text{n1}_{(\text{"(TCA) PDH"})}}}\right)\right)$$

$$-V_{\text{Mitochondria}}\cdot\left(\text{kcat}_{(\text{"(TCA) CS"})}\cdot[\text{enz\_CS}]\cdot\frac{[\text{OAA\{Mitochondria\}}]\cdot[\text{"Acetyl-CoA\{Mitochondria\}"}]}{\text{KmB}_{(\text{"(TCA) CS"})}\cdot[\text{OAA\{Mitochondria\}}]+\text{KmA}_{(\text{"(TCA) CS"})}\cdot[\text{"Acetyl-CoA\{Mitochondria\}"}]+[\text{OAA\{Mitochondria\}}]\cdot[\text{"Acetyl-CoA\{Mitochondria\}"}]+0.0000001}\right)$$

$$\frac{d\left([\text{PEP\{Cytosol\}}]\cdot V_{\text{Cytosol}}\right)}{d\,t} = -V_{\text{Cytosol}}\cdot\left(\text{kcat}_{(\text{"(GLY) PK"})}\cdot[\text{enz\_PK}]\cdot\frac{[\text{PEP\{Cytosol\}}]}{\text{Km}_{(\text{"(GLY) PK"})}+[\text{PEP\{Cytosol\}}]}\cdot\left(1+\frac{\text{c1}_{(\text{"(GLY) PK"})}\cdot[\text{"LANCL2-ABA"}]^{\text{n1}_{(\text{"(GLY) PK"})}}}{[\text{"LANCL2-ABA"}]^{\text{n1}_{(\text{"(GLY) PK"})}}+\text{ka}_{(\text{"(GLY) PK"})}^{\text{n1}_{(\text{"(GLY) PK"})}}}\right)\right)$$

$$
\begin{aligned}
&+V_{\text{Cytosol}}\cdot\left(\text{kcat}_{(\text{"(GLY) ENO\_F"})}\cdot[\text{enz\_ENO1}]\cdot\frac{[\text{"2-PG"}]}{\text{Km}_{(\text{"(GLY) ENO\_F"})}+[\text{"2-PG"}]}\right)\\
&+V_{\text{Cytosol}}\cdot\left(\text{kcat}_{(\text{"(GLU) PEPCK"})}\cdot[\text{enz\_PCK1}]\cdot\frac{[\text{OAA}\{\text{Cytosol}\}]}{\text{Km}_{(\text{"(GLU) PEPCK"})}+[\text{OAA}\{\text{Cytosol}\}]}\right)\\
&-V_{\text{Cytosol}}\cdot\left(\text{kcat}_{(\text{"(GLY) ENO\_R"})}\cdot[\text{enz\_ENO1}]\cdot\frac{[\text{PEP}\{\text{Cytosol}\}]}{\text{Km}_{(\text{"(GLY) ENO\_R"})}+[\text{PEP}\{\text{Cytosol}\}]}\right)
\end{aligned}
$$

$$
\begin{aligned}
\frac{d\left([\text{"F6-P"}]\cdot V_{\text{Cytosol}}\right)}{d\,t} = &-V_{\text{Cytosol}}\cdot\left(\text{kcat}_{(\text{"(GLY) PFK"})}\cdot[\text{enz\_PFK}]\cdot\frac{[\text{"F6-P"}]}{\text{Km}_{(\text{"(GLY) PFK"})}+[\text{"F6-P"}]}\right)\\
&+V_{\text{Cytosol}}\cdot\left(\text{kcat}_{(\text{"(GLY) GPI\_F"})}\cdot[\text{enz\_GPI}]\cdot\frac{[\text{"G6-P"}]}{\text{Km}_{(\text{"(GLY) GPI\_F"})}+[\text{"G6-P"}]}\right)\\
&+V_{\text{Cytosol}}\cdot\left(\text{kcat}_{(\text{"(PPP) TKT\_F6-P"})}\cdot[\text{enz\_TKT}]\cdot\frac{[\text{"X-5P"}]\cdot[\text{"E-5P"}]}{\text{KmB}_{(\text{"(PPP) TKT\_F6-P"})}\cdot[\text{"X-5P"}]+\text{KmA}_{(\text{"(PPP) TKT\_F6-P"})}\cdot[\text{"E-5P"}]+[\text{"X-5P"}]\cdot[\text{"E-5P"}]+0.0000001}\right)\\
&+V_{\text{Cytosol}}\cdot\left(\text{kcat}_{(\text{"(GLU) FBP"})}\cdot[\text{enz\_FBP}]\cdot\frac{[\text{"F1,6-BP"}]}{\text{Km}_{(\text{"(GLU) FBP"})}+[\text{"F1,6-BP"}]}\right)\\
&+V_{\text{Cytosol}}\cdot\left(\text{kcat}_{(\text{"(PPP) TAL"})}\cdot[\text{enz\_TAL}]\cdot\frac{[\text{SHL7P}]\cdot[\text{"G3-P"}]}{\text{KmB}_{(\text{"(PPP) TAL"})}\cdot[\text{SHL7P}]+\text{KmA}_{(\text{"(PPP) TAL"})}\cdot[\text{"G3-P"}]+[\text{SHL7P}]\cdot[\text{"G3-P"}]+0.0000001}\right)\\
&-V_{\text{Cytosol}}\cdot\left(\text{kcat}_{(\text{"(GLY) GPI\_R"})}\cdot[\text{enz\_GPI}]\cdot\frac{[\text{"F6-P"}]}{\text{Km}_{(\text{"(GLY) GPI\_R"})}+[\text{"F6-P"}]}\right)
\end{aligned}
$$

$$
\frac{d\left([\text{"L-3-Hydroxyacyl-CoA"}]\cdot V_{\text{Mitochondria}}\right)}{d\,t} = +V_{\text{Mitochondria}}\cdot\left(\text{kcat}_{(\text{"(FAO) ECH"})}\cdot[\text{enz\_ECH}]\cdot\frac{[\text{"Trans-enoyl-CoA"}]}{\text{Km}_{(\text{"(FAO) ECH"})}+[\text{"Trans-enoyl-CoA"}]}\right)
$$

$$- V_{\text{Mitochondria}} \cdot \left(\text{kcat}_{(\text{"(FAO) HADH"})} \cdot [\text{enz\_HADH}] \cdot \frac{[\text{"L-3-Hydroxyacyl-CoA"}]}{\text{Km}_{(\text{"(FAO) HADH"})} + [\text{"L-3-Hydroxyacyl-CoA"}]} \right)$$

$$[\text{enz\_OGDH}] = \text{Naive} \cdot 6.64\text{E-}08 + \text{Th1} \cdot 1.98\text{E-}07 + \text{Th2} \cdot 1.83\text{E-}07 + \text{Th17} \cdot 2.58\text{E-}07 + \text{Th22} \cdot 2.74\text{E-}07 + \text{Treg} \cdot 4.28\text{E-}07$$

$$\frac{d\left([\text{Lactate\{Cytosol\}}] \cdot V_{\text{Cytosol}}\right)}{d\,t} = - V_{\text{Cytosol}} \cdot \left(\text{kcat}_{(\text{"(GLY) MCT4"})} \cdot [\text{enz\_MCT4}] \cdot \frac{[\text{Lactate\{Cytosol\}}]}{\text{Km}_{(\text{"(GLY) MCT4"})} + [\text{Lactate\{Cytosol\}}]} \right)$$

$$+ V_{\text{Cytosol}} \cdot \left(\text{kcat}_{(\text{"(GLY) MCT1"})} \cdot [\text{enz\_MCT1}] \cdot \frac{[\text{Lactate\{Extracellular\}}]}{\text{Km}_{(\text{"(GLY) MCT1"})} + [\text{Lactate\{Extracellular\}}]} \right)$$

$$+ V_{\text{Cytosol}} \cdot \left(\text{kcat}_{(\text{"(GLY) LDHA"})} \cdot [\text{enz\_LDHA}] \cdot \frac{[\text{Pyruvate\{Cytosol\}}]}{\text{Km}_{(\text{"(GLY) LDHA"})} + [\text{Pyruvate\{Cytosol\}}]} \right)$$

$$- V_{\text{Cytosol}} \cdot \left(\text{kcat}_{(\text{"(GLU) LDHB"})} \cdot [\text{enz\_LDHB}] \cdot \frac{[\text{Lactate\{Cytosol\}}] \cdot [\text{NAD\_S\{Cytosol\}}]}{\text{KmB}_{(\text{"(GLU) LDHB"})} \cdot [\text{Lactate\{Cytosol\}}] + \text{KmA}_{(\text{"(GLU) LDHB"})} \cdot [\text{NAD\_S\{Cytosol\}}] + [\text{Lactate\{Cytosol\}}] \cdot [\text{NAD\_S\{Cytosol\}}] + 0.0000001} \right)$$

$$\frac{d\left([\text{Fumarate}] \cdot V_{\text{Mitochondria}}\right)}{d\,t} = - V_{\text{Mitochondria}} \cdot \left(\text{kcat}_{(\text{"(TCA) FH\_F"})} \cdot [\text{enz\_FH}] \cdot \frac{[\text{Fumarate}]}{\text{Km}_{(\text{"(TCA) FH\_F"})} + [\text{Fumarate}]} \right)$$

$$+ V_{\text{Cytosol}} \cdot \left(\text{kcat}_{(\text{"(TCA) SDH\_F"})} \cdot [\text{enz\_SDH}] \cdot \frac{[\text{Succinate}]}{\text{Km}_{(\text{"(TCA) SDH\_F"})} + [\text{Succinate}]} \right)$$

$$+ V_{\text{Mitochondria}} \cdot \left(\text{kcat}_{(\text{"(TCA) FH\_R"})} \cdot [\text{enz\_FH}] \cdot \frac{[\text{Malate\{Mitochondria\}}]}{\text{Km}_{(\text{"(TCA) FH\_R"})} + [\text{Malate\{Mitochondria\}}]} \right)$$

$$- V_{\text{Cytosol}} \cdot \left(\text{kcat}_{(\text{"(TCA) SDH\_R"})} \cdot [\text{enz\_SDH}] \cdot \frac{[\text{Fumarate}]}{\text{Km}_{(\text{"(TCA) SDH\_R"})} + [\text{Fumarate}]} \right)$$

$$\frac{d\left([\text{"G3-P"}] \cdot V_{\text{Cytosol}}\right)}{d\,t} = +2 \cdot V_{\text{Cytosol}} \cdot \left(\text{kcat}_{(\text{"(GLY) ALDO\_F"})} \cdot [\text{enz\_ALDO}] \cdot \frac{[\text{"F1,6-BP"}]}{\text{Km}_{(\text{"(GLY) ALDO\_F"})} + [\text{"F1,6-BP"}]} \right)$$

$$-V_{Cytosol} \cdot \left(kcat_{\text{("(GLY) GAPDH\_F")}} \cdot [\text{enz\_GAPDH}] \cdot \frac{[\text{"G3-P"}] \cdot [\text{NAD\_S\{Cytosol\}}]}{KmB_{\text{("(GLY) GAPDH\_F")}} \cdot [\text{"G3-P"}] + KmA_{\text{("(GLY) GAPDH\_F")}} \cdot [\text{NAD\_S\{Cytosol\}}] + [\text{"G3-P"}] \cdot [\text{NAD\_S\{Cytosol\}}] + 0.0000001} \right)$$

$$+V_{Cytosol} \cdot \left(kcat_{\text{("(PPP) TKT\_SHL7P")}} \cdot [\text{enz\_TKT}] \cdot \frac{[\text{"Ribose-5P"}] \cdot [\text{"X-5P"}]}{KmB_{\text{("(PPP) TKT\_SHL7P")}} \cdot [\text{"Ribose-5P"}] + KmA_{\text{("(PPP) TKT\_SHL7P")}} \cdot [\text{"X-5P"}] + [\text{"Ribose-5P"}] \cdot [\text{"X-5P"}] + 0.0000001} \right)$$

$$+V_{Cytosol} \cdot \left(kcat_{\text{("(PPP) TKT\_F6-P")}} \cdot [\text{enz\_TKT}] \cdot \frac{[\text{"X-5P"}] \cdot [\text{"E-5P"}]}{KmB_{\text{("(PPP) TKT\_F6-P")}} \cdot [\text{"X-5P"}] + KmA_{\text{("(PPP) TKT\_F6-P")}} \cdot [\text{"E-5P"}] + [\text{"X-5P"}] \cdot [\text{"E-5P"}] + 0.0000001} \right)$$

$$-2 \cdot V_{Cytosol} \cdot \left(kcat_{\text{("(GLY) ALDO\_R")}} \cdot [\text{enz\_ALDO}] \cdot \frac{[\text{"G3-P"}]}{Km_{\text{("(GLY) ALDO\_R")}} + [\text{"G3-P"}]} \right)$$

$$+V_{Cytosol} \cdot \left(kcat_{\text{("(GLY) GAPDH\_R")}} \cdot [\text{enz\_GAPDH}] \cdot \frac{[\text{"1,3-BPG"}]}{Km_{\text{("(GLY) GAPDH\_R")}} + [\text{"1,3-BPG"}]} \right)$$

$$-V_{Cytosol} \cdot \left(kcat_{\text{("(PPP) TAL")}} \cdot [\text{enz\_TAL}] \cdot \frac{[\text{SHL7P}] \cdot [\text{"G3-P"}]}{KmB_{\text{("(PPP) TAL")}} \cdot [\text{SHL7P}] + KmA_{\text{("(PPP) TAL")}} \cdot [\text{"G3-P"}] + [\text{SHL7P}] \cdot [\text{"G3-P"}] + 0.0000001} \right)$$

$$[\text{enz\_ECH}] = Naive \cdot 1.03E\text{-}07 + Th1 \cdot 1.31E\text{-}07 + Th2 \cdot 1.29E\text{-}07 + Th17 \cdot 1.33E\text{-}07 + Th22 \cdot 1.34E\text{-}07 + Treg \cdot 1.97E\text{-}07$$

$$[\text{enz\_PFK}] = Naive \cdot 4.03E\text{-}07 + Th1 \cdot 5.47E\text{-}07 + Th2 \cdot 5.95E\text{-}07 + Th17 \cdot 7.20E\text{-}07 + Th22 \cdot 9.29E\text{-}07 + Treg \cdot 9.29E\text{-}07$$

$$[\text{enz\_HADH}] = Naive \cdot 6.57E\text{-}08 + Th1 \cdot 4.76E\text{-}08 + Th2 \cdot 4.58E\text{-}08 + Th17 \cdot 4.63E\text{-}08 + Th22 \cdot 4.83E\text{-}08 + Treg \cdot 5.19E\text{-}08$$

$$[\text{enz\_CACT}] = \begin{cases} Naive \cdot 1.49E\text{-}08 + Th1 \cdot 2.59E\text{-}08 + Th2 \cdot 2.24E\text{-}08 + Th17 \cdot 2.66E\text{-}08 + Th22 \cdot 3.13E\text{-}08 + Treg \cdot 5.50E\text{-}08 - \text{"[enz\_CACT-M]"} <= 1e\text{-}13, & 1e\text{-}13 \\ \text{else,} & Naive \cdot 1.49E\text{-}08 + Th1 \cdot 2.59E\text{-}08 + Th2 \cdot 2.24E\text{-}08 + Th17 \cdot 2.66E\text{-}08 + Th22 \cdot 3.13E\text{-}08 + Treg \cdot 5.50E\text{-}08 - \text{"[enz\_CACT-M]"} \end{cases}$$

$$[\text{enz\_CPT1a}] = Naive \cdot 5.16E\text{-}08 + Th1 \cdot 8.98E\text{-}08 + Th2 \cdot 8.12E\text{-}08 + Th17 \cdot 7.52E\text{-}08 + Th22 \cdot 1.02E\text{-}07 + Treg \cdot 7.26E\text{-}08$$

$$[\text{enz\_CPT2}] = Naive \cdot 5.16E\text{-}08 + Th1 \cdot 4.69E\text{-}08 + Th2 \cdot 4.79E\text{-}08 + Th17 \cdot 3.91E\text{-}08 + Th22 \cdot 3.66E\text{-}08 + Treg \cdot 7.85E\text{-}08$$

$$[\text{enz\_ENO1}] = Naive \cdot 8.08E\text{-}07 + Th1 \cdot 1.05E\text{-}06 + Th2 \cdot 1.09E\text{-}06 + Th17 \cdot 1.30E\text{-}06 + Th22 \cdot 1.10E\text{-}06 + Treg \cdot 1.60E\text{-}06$$

$$[\text{enz\_GLUT}] = Naive \cdot 2.34E\text{-}07 + Th1 \cdot 2.36E\text{-}07 + Th2 \cdot 2.51E\text{-}07 + Th17 \cdot 2.38E\text{-}07 + Th22 \cdot 2.25E\text{-}07 + Treg \cdot 2.19E\text{-}07$$

$$[\text{enz\_SDH}] = Naive \cdot 2.69E\text{-}07 + Th1 \cdot 2.87E\text{-}07 + Th2 \cdot 2.34E\text{-}07 + Th17 \cdot 2.32E\text{-}07 + Th22 \cdot 2.45E\text{-}07 + Treg \cdot 3.21E\text{-}07$$

$$[\text{enz\_PDH}] = \begin{cases} \text{Naive} \cdot 1.98\text{E-}07 + \text{Th1} \cdot 1.53\text{E-}07 + \text{Th2} \cdot 1.53\text{E-}07 + \text{Th17} \cdot 1.54\text{E-}07 + \text{Th22} \cdot 1.30\text{E-}07 + \text{Treg} \cdot 2.23\text{E-}07 - \text{"[PDH-P]"} <= 1\text{e-}13, & 1\text{e-}13 \\ \text{else}, & \text{Naive} \cdot 1.98\text{E-}07 + \text{Th1} \cdot 1.53\text{E-}07 + \text{Th2} \cdot 1.53\text{E-}07 + \text{Th17} \cdot 1.54\text{E-}07 + \text{Th22} \cdot 1.30\text{E-}07 + \text{Treg} \cdot 2.23\text{E-}07 - \text{"[PDH-P]"} \end{cases}$$

$$[\text{enz\_ACAA}] = \text{Naive} \cdot 2.71\text{E-}07 + \text{Th1} \cdot 1.97\text{E-}07 + \text{Th2} \cdot 2.14\text{E-}07 + \text{Th17} \cdot 2.47\text{E-}07 + \text{Th22} \cdot 2.43\text{E-}07 + \text{Treg} \cdot 3.24\text{E-}07$$

$$[\text{enz\_HK}] = \text{Naive} \cdot 3.07\text{E-}07 + \text{Th1} \cdot 2.41\text{E-}07 + \text{Th2} \cdot 2.84\text{E-}07 + \text{Th17} \cdot 3.27\text{E-}07 + \text{Th22} \cdot 3.67\text{E-}07 + \text{Treg} \cdot 3.29\text{E-}07$$

$$[\text{enz\_FH}] = \text{Naive} \cdot 8.19\text{E-}08 + \text{Th1} \cdot 6.81\text{E-}08 + \text{Th2} \cdot 6.00\text{E-}08 + \text{Th17} \cdot 7.29\text{E-}08 + \text{Th22} \cdot 5.59\text{E-}08 + \text{Treg} \cdot 9.82\text{E-}08$$

$$[\text{enz\_ACAD}] = \text{Naive} \cdot 3.05\text{E-}08 + \text{Th1} \cdot 3.70\text{E-}08 + \text{Th2} \cdot 3.48\text{E-}08 + \text{Th17} \cdot 3.83\text{E-}08 + \text{Th22} \cdot 2.97\text{E-}08 + \text{Treg} \cdot 4.94\text{E-}08$$

$$[\text{enz\_PK}] = \text{Naive} \cdot 5.52\text{E-}07 + \text{Th1} \cdot 7.61\text{E-}07 + \text{Th2} \cdot 9.91\text{E-}07 + \text{Th17} \cdot 1.07\text{E-}06 + \text{Th22} \cdot 1.21\text{E-}06 + \text{Treg} \cdot 1.15\text{E-}06$$

$$[\text{enz\_ACO}] = \text{Naive} \cdot 1.83\text{E-}07 + \text{Th1} \cdot 2.42\text{E-}07 + \text{Th2} \cdot 2.40\text{E-}07 + \text{Th17} \cdot 2.32\text{E-}07 + \text{Th22} \cdot 2.32\text{E-}07 + \text{Treg} \cdot 2.47\text{E-}07$$

$$[\text{enz\_GPI}] = \text{Naive} \cdot 1.69\text{E-}07 + \text{Th1} \cdot 1.90\text{E-}07 + \text{Th2} \cdot 2.21\text{E-}07 + \text{Th17} \cdot 2.32\text{E-}07 + \text{Th22} \cdot 2.30\text{E-}07 + \text{Treg} \cdot 2.47\text{E-}07$$

$$[\text{enz\_PGK}] = \text{Naive} \cdot 3.55\text{E-}07 + \text{Th1} \cdot 4.60\text{E-}07 + \text{Th2} \cdot 5.38\text{E-}07 + \text{Th17} \cdot 4.45\text{E-}07 + \text{Th22} \cdot 5.48\text{E-}07 + \text{Treg} \cdot 4.09\text{E-}07$$

$$[\text{enz\_IDH}] = \text{Naive} \cdot 1.85\text{E-}07 + \text{Th1} \cdot 2.47\text{E-}07 + \text{Th2} \cdot 2.36\text{E-}07 + \text{Th17} \cdot 2.13\text{E-}07 + \text{Th22} \cdot 2.11\text{E-}07 + \text{Treg} \cdot 2.29\text{E-}07$$

$$[\text{enz\_ALDO}] = \text{Naive} \cdot 1.42\text{E-}06 + \text{Th1} \cdot 2.08\text{E-}06 + \text{Th2} \cdot 2.31\text{E-}06 + \text{Th17} \cdot 2.14\text{E-}06 + \text{Th22} \cdot 2.12\text{E-}06 + \text{Treg} \cdot 2.46\text{E-}06$$

$$[\text{enz\_SUCL}] = \text{Naive} \cdot 4.33\text{E-}08 + \text{Th1} \cdot 4.68\text{E-}08 + \text{Th2} \cdot 4.90\text{E-}08 + \text{Th17} \cdot 4.10\text{E-}08 + \text{Th22} \cdot 4.40\text{E-}08 + \text{Treg} \cdot 5.75\text{E-}08$$

$$[\text{enz\_CS}] = \text{Naive} \cdot 5.33\text{E-}07 + \text{Th1} \cdot 6.05\text{E-}07 + \text{Th2} \cdot 6.34\text{E-}07 + \text{Th17} \cdot 6.97\text{E-}07 + \text{Th22} \cdot 6.49\text{E-}07 + \text{Treg} \cdot 6.45\text{E-}07$$

$$\frac{d\left([\text{Glutamine\{Mitochondria\}}] \cdot V_{\text{Mitochondria}}\right)}{d\,t} = + V_{\text{Cytosol}} \cdot \left(\text{kcat}_{(\text{"(Glutaminolysis) ASCT2\_F"})} \cdot [\text{enz\_ASCT2}] \cdot \frac{[\text{Glutamine\{Cytosol\}}]}{\text{Km}_{(\text{"(Glutaminolysis) ASCT2\_F"})} + [\text{Glutamine\{Cytosol\}}]}\right)$$
$$- V_{\text{Cytosol}} \cdot \left(\text{kcat}_{(\text{"(Glutaminolysis) GLS\_F"})} \cdot [\text{enz\_GLS}] \cdot \frac{[\text{Glutamine\{Mitochondria\}}]}{\text{Km}_{(\text{"(Glutaminolysis) GLS\_F"})} + [\text{Glutamine\{Mitochondria\}}]}\right)$$
$$+ V_{\text{Cytosol}} \cdot \left(\text{kcat}_{(\text{"(Glutaminolysis) GLS\_R"})} \cdot [\text{enz\_GLS}] \cdot \frac{[\text{Glutamate\{Mitochondria\}}]}{\text{Km}_{(\text{"(Glutaminolysis) GLS\_R"})} + [\text{Glutamate\{Mitochondria\}}]}\right)$$
$$- V_{\text{Cytosol}} \cdot \left(\text{kcat}_{(\text{"(Glutaminolysis) ASCT2\_R"})} \cdot [\text{enz\_ASCT2}] \cdot \frac{[\text{Glutamine\{Mitochondria\}}]}{\text{Km}_{(\text{"(Glutaminolysis) ASCT2\_R"})} + [\text{Glutamine\{Mitochondria\}}]}\right)$$

$$\frac{d\left(\text{[NAD\_P\_GAPDH]}\cdot V_{\text{Cytosol}}\right)}{dt} = +V_{\text{Cytosol}}\cdot\left(\text{kcat}_{\text{("(GLY) GAPDH\_R")}}\cdot\text{[enz\_GAPDH]}\cdot\frac{\text{["1,3-BPG"]}}{\text{Km}_{\text{("(GLY) GAPDH\_R")}}+\text{["1,3-BPG"]}}\right)$$

$$\text{[enz\_ComplexII]} = \text{Naive}\cdot 7.71\text{E-}08+\text{Th1}\cdot 7.32\text{E-}08+\text{Th2}\cdot 6.47\text{E-}08+\text{Th17}\cdot 8.81\text{E-}08+\text{Th22}\cdot 1.74\text{E-}08+\text{Treg}\cdot 1.20\text{E-}07$$

$$\frac{d\left(\text{[ATP\_P\_OXPHOS]}\cdot V_{\text{Mitochondria}}\right)}{dt} = +V_{\text{Intermembrane}}\cdot\left(0\cdot\left(\text{[H\{Intermembrane\}]}+\text{[H\{Intermembrane\}]}+\text{[H\{Intermembrane\}]}+\text{[H\{Mitochondria\}]}+\text{[H\{Mitochondria\}]}+\text{[H\{Mitochondria\}]}\right)+\text{[enz\_ATPase]}\cdot\text{kcat}_{\text{("(OXPHOS) V")}}\cdot\left(\text{Vf}_{\text{("(OXPHOS) V")}}\cdot\text{[H\{Intermembrane\}]}\cdot\frac{\text{[ADP\_S\{Mitochondria\}]}}{\text{[ADP\_S\{Mitochondria\}]}+\text{Km1}_{\text{("(OXPHOS) V")}}}-\text{Vr}_{\text{("(OXPHOS) V")}}\cdot\text{[H\{Mitochondria\}]}\cdot\frac{\text{[ATP\_P\_OXPHOS]}}{\text{[ATP\_P\_OXPHOS]}+\text{Km2}_{\text{("(OXPHOS) V")}}}\right)\right)$$

$$\text{[enz\_GLUD]} = \text{Naive}\cdot 3.12\text{E-}07+\text{Th1}\cdot 3.14\text{E-}07+\text{Th2}\cdot 3.43\text{E-}07+\text{Th17}\cdot 3.49\text{E-}07+\text{Th22}\cdot 3.99\text{E-}07+\text{Treg}\cdot 3.89\text{E-}07$$

$$\frac{d\left(\text{["a-KG\{Cytosol\}"]}\cdot V_{\text{Cytosol}}\right)}{dt} = -V_{\text{Cytosol}}\cdot\left(\text{kcat}_{\text{("(MAS) GOT1")}}\cdot\text{[enz\_GOT1]}\cdot\frac{\text{[Aspartate\{Cytosol\}]}\cdot\text{["a-KG\{Cytosol\}"]}}{\text{KmB}_{\text{("(MAS) GOT1")}}\cdot\text{[Aspartate\{Cytosol\}]}+\text{KmA}_{\text{("(MAS) GOT1")}}\cdot\text{["a-KG\{Cytosol\}"]}+\text{[Aspartate\{Cytosol\}]}\cdot\text{["a-KG\{Cytosol\}"]}+0.0000001}\right)$$

$$+V_{\text{Cytosol}}\cdot\left(\text{kcat}_{\text{("(MAS) M-aKG Transport")}}\cdot\text{[enz\_SLC25A11]}\cdot\frac{\text{[Malate\{Cytosol\}]}\cdot\text{["a-KG\{Mitochondria\}"]}}{\text{KmB}_{\text{("(MAS) M-aKG Transport")}}\cdot\text{[Malate\{Cytosol\}]}+\text{KmA}_{\text{("(MAS) M-aKG Transport")}}\cdot\text{["a-KG\{Mitochondria\}"]}+\text{[Malate\{Cytosol\}]}\cdot\text{["a-KG\{Mitochondria\}"]}+0.0000001}\right)$$

$$\text{[enz\_GLS]} = \text{Naive}\cdot 2.26\text{E-}07+\text{Th1}\cdot 2.48\text{E-}07+\text{Th2}\cdot 2.29\text{E-}07+\text{Th17}\cdot 2.58\text{E-}07+\text{Th22}\cdot 2.97\text{E-}07+\text{Treg}\cdot 4.03\text{E-}07$$

$$\frac{d\left(\text{[NADH\_P\_GLUD]}\cdot V_{\text{Mitochondria}}\right)}{dt} = +V_{\text{Mitochondria}}\cdot\left(\text{kcat}_{\text{("(Glutaminolysis) GLUD\_F")}}\cdot\text{[enz\_GLUD]}\cdot\frac{\text{[Glutamate\{Mitochondria\}]}\cdot\text{[NAD\_S\{Mitochondria\}]}}{\text{KmB}_{\text{("(Glutaminolysis) GLUD\_F")}}\cdot\text{[Glutamate\{Mitochondria\}]}+\text{KmA}_{\text{("(Glutaminolysis) GLUD\_F")}}\cdot\text{[NAD\_S\{Mitochondria\}]}+\text{[Glutamate\{Mitochondria\}]}\cdot\text{[NAD\_S\{Mitochondria\}]}+0.0000001}\right)$$

$$\text{[enz\_MDH2]} = \text{Naive}\cdot 3.40\text{E-}07+\text{Th1}\cdot 3.61\text{E-}07+\text{Th2}\cdot 3.88\text{E-}07+\text{Th17}\cdot 4.34\text{E-}07+\text{Th22}\cdot 3.52\text{E-}07+\text{Treg}\cdot 4.99\text{E-}07$$

$$\frac{d\left(\text{[SHL7P]}\cdot V_{\text{Cytosol}}\right)}{dt} = +V_{\text{Cytosol}}\cdot\left(\text{kcat}_{\text{("(PPP) TKT\_SHL7P")}}\cdot\text{[enz\_TKT]}\cdot\frac{\text{["Ribose-5P"]}\cdot\text{["X-5P"]}}{\text{KmB}_{\text{("(PPP) TKT\_SHL7P")}}\cdot\text{["Ribose-5P"]}+\text{KmA}_{\text{("(PPP) TKT\_SHL7P")}}\cdot\text{["X-5P"]}+\text{["Ribose-5P"]}\cdot\text{["X-5P"]}+0.0000001}\right)$$

$$-V_{\text{Cytosol}}\cdot\left(\text{kcat}_{\text{("(PPP) TAL")}}\cdot\text{[enz\_TAL]}\cdot\frac{\text{[SHL7P]}\cdot\text{["G3-P"]}}{\text{KmB}_{\text{("(PPP) TAL")}}\cdot\text{[SHL7P]}+\text{KmA}_{\text{("(PPP) TAL")}}\cdot\text{["G3-P"]}+\text{[SHL7P]}\cdot\text{["G3-P"]}+0.0000001}\right)$$

$$\frac{d\left(\text{[Malate\{Cytosol\}]}\cdot V_{\text{Cytosol}}\right)}{dt} = +V_{\text{Cytosol}}\cdot\left(\text{kcat}_{\text{("(MAS) MDH1")}}\cdot\text{[enz\_MDH1]}\cdot\frac{\text{[OAA\{Cytosol\}]}}{\text{Km}_{\text{("(MAS) MDH1")}}+\text{[OAA\{Cytosol\}]}}\right)$$

$$-V_{\text{Cytosol}}\cdot\left(\text{kcat}_{(\text{"(MAS) M-aKG Transport"})}\cdot[\text{enz\_SLC25A11}]\cdot\frac{[\text{Malate\{Cytosol\}}]\cdot[\text{"a-KG\{Mitochondria\}"}]}{\text{KmB}_{(\text{"(MAS) M-aKG Transport"})}\cdot[\text{Malate\{Cytosol\}}]+\text{KmA}_{(\text{"(MAS) M-aKG Transport"})}\cdot[\text{"a-KG\{Mitochondria\}"}]+[\text{Malate\{Cytosol\}}]\cdot[\text{"a-KG\{Mitochondria\}"}]+0.0000001}\right)$$

$$-V_{\text{Mitochondria}}\cdot\left(\text{kcat}_{(\text{"(Glutaminolysis) ME"})}\cdot[\text{enz\_ME1}]\cdot\frac{[\text{Malate\{Cytosol\}}]}{\text{Km}_{(\text{"(Glutaminolysis) ME"})}+[\text{Malate\{Cytosol\}}]}\right)$$

$$\frac{d\left([\text{NAD\_P\_GLUD}]\cdot V_{\text{Mitochondria}}\right)}{d\,t}=+V_{\text{Mitochondria}}\cdot\left(\text{kcat}_{(\text{"(Glutaminolysis) GLUD\_R"})}\cdot[\text{enz\_GLUD}]\cdot\frac{[\text{"a-KG\{Mitochondria\}"}]}{\text{Km}_{(\text{"(Glutaminolysis) GLUD\_R"})}+[\text{"a-KG\{Mitochondria\}"}]}\right)$$

$$\frac{d\left([\text{OAA\{Cytosol\}}]\cdot V_{\text{Cytosol}}\right)}{d\,t}=+V_{\text{Cytosol}}\cdot\left(\text{kcat}_{(\text{"(FAS) ACLY\_F"})}\cdot[\text{enz\_ACLY}]\cdot\frac{[\text{Citrate\{Cytosol\}}]}{\text{Km}_{(\text{"(FAS) ACLY\_F"})}+[\text{Citrate\{Cytosol\}}]}\right)$$

$$-V_{\text{Cytosol}}\cdot\left(\text{kcat}_{(\text{"(FAS) ACLY\_R"})}\cdot[\text{enz\_ACLY}]\cdot\frac{[\text{"Acetyl-CoA\{Cytosol\}"}]\cdot[\text{OAA\{Cytosol\}}]}{\text{kia}_{(\text{"(FAS) ACLY\_R"})}\cdot\text{KmB}_{(\text{"(FAS) ACLY\_R"})}+\text{KmB}_{(\text{"(FAS) ACLY\_R"})}\cdot[\text{"Acetyl-CoA\{Cytosol\}"}]+\text{KmA}_{(\text{"(FAS) ACLY\_R"})}\cdot[\text{OAA\{Cytosol\}}]+[\text{"Acetyl-CoA\{Cytosol\}"}]\cdot[\text{OAA\{Cytosol\}}]}\right)$$

$$-V_{\text{Cytosol}}\cdot\left(\text{kcat}_{(\text{"(GLU) PEPCK"})}\cdot[\text{enz\_PCK1}]\cdot\frac{[\text{OAA\{Cytosol\}}]}{\text{Km}_{(\text{"(GLU) PEPCK"})}+[\text{OAA\{Cytosol\}}]}\right)$$

$$+V_{\text{Cytosol}}\cdot\left(\text{kcat}_{(\text{"(MAS) GOT1"})}\cdot[\text{enz\_GOT1}]\cdot\frac{[\text{Aspartate\{Cytosol\}}]\cdot[\text{"a-KG\{Cytosol\}"}]}{\text{KmB}_{(\text{"(MAS) GOT1"})}\cdot[\text{Aspartate\{Cytosol\}}]+\text{KmA}_{(\text{"(MAS) GOT1"})}\cdot[\text{"a-KG\{Cytosol\}"}]+[\text{Aspartate\{Cytosol\}}]\cdot[\text{"a-KG\{Cytosol\}"}]+0.0000001}\right)$$

$$-V_{\text{Cytosol}}\cdot\left(\text{kcat}_{(\text{"(MAS) MDH1"})}\cdot[\text{enz\_MDH1}]\cdot\frac{[\text{OAA\{Cytosol\}}]}{\text{Km}_{(\text{"(MAS) MDH1"})}+[\text{OAA\{Cytosol\}}]}\right)$$

$$\frac{d\left([\text{"Acyl-CoA\{Mitochondria\}"}]\cdot V_{\text{Mitochondria}}\right)}{d\,t}=+V_{\text{Cytosol}}\cdot\left(\text{kcat}_{(\text{"(FAO) CPT2"})}\cdot[\text{enz\_CPT2}]\cdot\frac{[\text{Acyl\_Carnitine\{Mitochondria\}}]}{\text{Km}_{(\text{"(FAO) CPT2"})}+[\text{Acyl\_Carnitine\{Mitochondria\}}]}\right)$$

$$-V_{\text{Mitochondria}}\cdot\left(\text{kcat}_{(\text{"(FAO) ACAD"})}\cdot[\text{enz\_ACAD}]\cdot\frac{[\text{"Acyl-CoA\{Mitochondria\}"}]}{\text{Km}_{(\text{"(FAO) ACAD"})}+[\text{"Acyl-CoA\{Mitochondria\}"}]}\right)$$

$$\begin{aligned}\frac{d\left(\text{["Ribose-5P"]}\cdot V_{\text{Cytosol}}\right)}{d\,t} &= +V_{\text{Cytosol}}\cdot\left(\text{kcat}_{\text{("(PPP) RPI")}}\cdot\text{[enz\_RPI]}\cdot\frac{\text{["Ribulose-5P"]}}{\text{Km}_{\text{("(PPP) RPI")}}+\text{["Ribulose-5P"]}}\right)\\ &\quad -V_{\text{Cytosol}}\cdot\left(\text{kcat}_{\text{("(PPP) TKT\_SHL7P")}}\cdot\text{[enz\_TKT]}\cdot\frac{\text{["Ribose-5P"]}\cdot\text{["X-5P"]}}{\text{KmB}_{\text{("(PPP) TKT\_SHL7P")}}\cdot\text{["Ribose-5P"]}+\text{KmA}_{\text{("(PPP) TKT\_SHL7P")}}\cdot\text{["X-5P"]}+\text{["Ribose-5P"]}\cdot\text{["X-5P"]}+0.0000001}\right)\end{aligned}$$

$$\begin{aligned}\frac{d\left(\text{["E-5P"]}\cdot V_{\text{Cytosol}}\right)}{d\,t} &= -V_{\text{Cytosol}}\cdot\left(\text{kcat}_{\text{("(PPP) TKT\_F6-P")}}\cdot\text{[enz\_TKT]}\cdot\frac{\text{["X-5P"]}\cdot\text{["E-5P"]}}{\text{KmB}_{\text{("(PPP) TKT\_F6-P")}}\cdot\text{["X-5P"]}+\text{KmA}_{\text{("(PPP) TKT\_F6-P")}}\cdot\text{["E-5P"]}+\text{["X-5P"]}\cdot\text{["E-5P"]}+0.0000001}\right)\\ &\quad +V_{\text{Cytosol}}\cdot\left(\text{kcat}_{\text{("(PPP) TAL")}}\cdot\text{[enz\_TAL]}\cdot\frac{\text{[SHL7P]}\cdot\text{["G3-P"]}}{\text{KmB}_{\text{("(PPP) TAL")}}\cdot\text{[SHL7P]}+\text{KmA}_{\text{("(PPP) TAL")}}\cdot\text{["G3-P"]}+\text{[SHL7P]}\cdot\text{["G3-P"]}+0.0000001}\right)\end{aligned}$$

$$\begin{aligned}\frac{d\left(\text{[Glutamate\{Mitochondria\}]}\cdot V_{\text{Mitochondria}}\right)}{d\,t} &= -V_{\text{Mitochondria}}\cdot\left(\text{kcat}_{\text{("(MAS) GOT2")}}\cdot\text{[enz\_GOT2]}\cdot\frac{\text{[OAA\{Mitochondria\}]}\cdot\text{[Glutamate\{Mitochondria\}]}}{\text{KmB}_{\text{("(MAS) GOT2")}}\cdot\text{[OAA\{Mitochondria\}]}+\text{KmA}_{\text{("(MAS) GOT2")}}\cdot\text{[Glutamate\{Mitochondria\}]}+\text{[OAA\{Mitochondria\}]}\cdot\text{[Glutamate\{Mitochondria\}]}+0.0000001}\right)\\ &\quad +V_{\text{Mitochondria}}\cdot\left(\text{kcat}_{\text{("(MAS) A-G Transport")}}\cdot\text{[enz\_SLC1A3]}\cdot\frac{\text{[Aspartate\{Mitochondria\}]}\cdot\text{[Glutamate\{Cytosol\}]}}{\text{KmB}_{\text{("(MAS) A-G Transport")}}\cdot\text{[Aspartate\{Mitochondria\}]}+\text{KmA}_{\text{("(MAS) A-G Transport")}}\cdot\text{[Glutamate\{Cytosol\}]}+\text{[Aspartate\{Mitochondria\}]}\cdot\text{[Glutamate\{Cytosol\}]}+0.0000001}\right)\\ &\quad +V_{\text{Cytosol}}\cdot\left(\text{kcat}_{\text{("(Glutaminolysis) GLS\_F")}}\cdot\text{[enz\_GLS]}\cdot\frac{\text{[Glutamine\{Mitochondria\}]}}{\text{Km}_{\text{("(Glutaminolysis) GLS\_F")}}+\text{[Glutamine\{Mitochondria\}]}}\right)\\ &\quad -V_{\text{Cytosol}}\cdot\left(\text{kcat}_{\text{("(Glutaminolysis) GLS\_R")}}\cdot\text{[enz\_GLS]}\cdot\frac{\text{[Glutamate\{Mitochondria\}]}}{\text{Km}_{\text{("(Glutaminolysis) GLS\_R")}}+\text{[Glutamate\{Mitochondria\}]}}\right)\\ &\quad +V_{\text{Mitochondria}}\cdot\left(\text{kcat}_{\text{("(Glutaminolysis) GLUD\_R")}}\cdot\text{[enz\_GLUD]}\cdot\frac{\text{["a-KG\{Mitochondria\}"]}}{\text{Km}_{\text{("(Glutaminolysis) GLUD\_R")}}+\text{["a-KG\{Mitochondria\}"]}}\right)\\ &\quad -V_{\text{Mitochondria}}\cdot\left(\text{kcat}_{\text{("(Glutaminolysis) GLUD\_F")}}\cdot\text{[enz\_GLUD]}\cdot\frac{\text{[Glutamate\{Mitochondria\}]}\cdot\text{[NAD\_S\{Mitochondria\}]}}{\text{KmB}_{\text{("(Glutaminolysis) GLUD\_F")}}\cdot\text{[Glutamate\{Mitochondria\}]}+\text{KmA}_{\text{("(Glutaminolysis) GLUD\_F")}}\cdot\text{[NAD\_S\{Mitochondria\}]}+\text{[Glutamate\{Mitochondria\}]}\cdot\text{[NAD\_S\{Mitochondria\}]}+0.0000001}\right)\end{aligned}$$

$$\frac{d\left([\text{ADP\_P\_PGK}]\cdot V_{\text{Cytosol}}\right)}{dt} = +V_{\text{Cytosol}}\cdot\left(kcat_{(\text{"(GLY) PGK\_R"})}\cdot[\text{enz\_PGK}]\cdot\frac{[\text{"3-PG"}]}{Km_{(\text{"(GLY) PGK\_R"})}+[\text{"3-PG"}]}\right)$$

$$\frac{d\left([\text{"Acyl-CoA\{Cytosol\}"}]\cdot V_{\text{Cytosol}}\right)}{dt} = +V_{\text{Cytosol}}\cdot\left(kcat_{(\text{"(FAO) ACSL"})}\cdot[\text{enz\_ACSL}]\cdot\frac{[\text{FA\{Cytosol\}}]}{Km_{(\text{"(FAO) ACSL"})}+[\text{FA\{Cytosol\}}]}\right)$$

$$-V_{\text{Cytosol}}\cdot\left(kcat_{(\text{"(FAO) CPT1a"})}\cdot[\text{enz\_CPT1a}]\cdot\frac{[\text{"Acyl-CoA\{Cytosol\}"}]}{Km_{(\text{"(FAO) CPT1a"})}+[\text{"Acyl-CoA\{Cytosol\}"}]}\right)$$

$$[\text{enz\_FBP}] = \text{Naive}\cdot 3.52\text{E-}08+\text{Th1}\cdot 1.10\text{E-}08+\text{Th2}\cdot 5.47\text{E-}09+\text{Th17}\cdot 5.29\text{E-}09+\text{Th22}\cdot 3.06\text{E-}09+\text{Treg}\cdot 6.11\text{E-}10$$

$$[\text{enz\_SLC1A3}] = \text{Naive}\cdot 1.00\text{E-}10+\text{Th1}\cdot 1.00\text{E-}10+\text{Th2}\cdot 1.00\text{E-}10+\text{Th17}\cdot 1.00\text{E-}10+\text{Th22}\cdot 1.00\text{E-}10+\text{Treg}\cdot 1.00\text{E-}10$$

$$[\text{enz\_GOT1}] = \text{Naive}\cdot 3.11\text{E-}08+\text{Th1}\cdot 4.86\text{E-}08+\text{Th2}\cdot 4.41\text{E-}08+\text{Th17}\cdot 4.40\text{E-}08+\text{Th22}\cdot 5.94\text{E-}08+\text{Treg}\cdot 7.03\text{E-}08$$

$$\frac{d\left([\text{NAD\_P\_MDH1}]\cdot V_{\text{Cytosol}}\right)}{dt} = +V_{\text{Cytosol}}\cdot\left(kcat_{(\text{"(MAS) MDH1"})}\cdot[\text{enz\_MDH1}]\cdot\frac{[\text{OAA\{Cytosol\}}]}{Km_{(\text{"(MAS) MDH1"})}+[\text{OAA\{Cytosol\}}]}\right)$$

$$[\text{enz\_GOT2}] = \text{Naive}\cdot 7.81\text{E-}08+\text{Th1}\cdot 1.09\text{E-}07+\text{Th2}\cdot 1.02\text{E-}07+\text{Th17}\cdot 1.38\text{E-}07+\text{Th22}\cdot 1.11\text{E-}07+\text{Treg}\cdot 1.52\text{E-}07$$

$$[\text{enz\_SLC27A1}] = \text{Naive}\cdot 1.98\text{E-}08+\text{Th1}\cdot 1.47\text{E-}08+\text{Th2}\cdot 1.45\text{E-}08+\text{Th17}\cdot 1.53\text{E-}08+\text{Th22}\cdot 1.80\text{E-}08+\text{Treg}\cdot 1.18\text{E-}08$$

$$[\text{enz\_MDH1}] = \text{Naive}\cdot 4.04\text{E-}07+\text{Th1}\cdot 4.18\text{E-}07+\text{Th2}\cdot 4.60\text{E-}07+\text{Th17}\cdot 3.97\text{E-}07+\text{Th22}\cdot 4.10\text{E-}07+\text{Treg}\cdot 5.61\text{E-}07$$

$$[\text{enz\_ASCT2}] = \text{Naive}\cdot 2.47\text{E-}08+\text{Th1}\cdot 6.73\text{E-}08+\text{Th2}\cdot 8.63\text{E-}08+\text{Th17}\cdot 6.35\text{E-}08+\text{Th22}\cdot 9.93\text{E-}08+\text{Treg}\cdot 8.53\text{E-}08$$

$$[\text{enz\_RPI}] = \text{Naive}\cdot 2.85\text{E-}07+\text{Th1}\cdot 2.31\text{E-}07+\text{Th2}\cdot 2.27\text{E-}07+\text{Th17}\cdot 2.57\text{E-}07+\text{Th22}\cdot 2.47\text{E-}07+\text{Treg}\cdot 2.58\text{E-}07$$

$$[\text{enz\_TKT}] = \text{Naive}\cdot 3.69\text{E-}07+\text{Th1}\cdot 2.93\text{E-}07+\text{Th2}\cdot 3.59\text{E-}07+\text{Th17}\cdot 3.84\text{E-}07+\text{Th22}\cdot 3.57\text{E-}07+\text{Treg}\cdot 4.03\text{E-}07$$

$$\frac{d\left([\text{Aspartate\{Mitochondria\}}]\cdot V_{\text{Mitochondria}}\right)}{dt} = +V_{\text{Mitochondria}}\cdot\left(kcat_{(\text{"(MAS) GOT2"})}\cdot[\text{enz\_GOT2}]\cdot\frac{[\text{OAA\{Mitochondria\}}]\cdot[\text{Glutamate\{Mitochondria\}}]}{KmB_{(\text{"(MAS) GOT2"})}\cdot[\text{OAA\{Mitochondria\}}]+KmA_{(\text{"(MAS) GOT2"})}\cdot[\text{Glutamate\{Mitochondria\}}]+[\text{OAA\{Mitochondria\}}]\cdot[\text{Glutamate\{Mitochondria\}}]+0.0000001}\right)$$

$$-V_{\text{Mitochondria}}\cdot\left(kcat_{(\text{"(MAS) A-G Transport"})}\cdot[\text{enz\_SLC1A3}]\cdot\frac{[\text{Aspartate\{Mitochondria\}}]\cdot[\text{Glutamate\{Cytosol\}}]}{KmB_{(\text{"(MAS) A-G Transport"})}\cdot[\text{Aspartate\{Mitochondria\}}]+KmA_{(\text{"(MAS) A-G Transport"})}\cdot[\text{Glutamate\{Cytosol\}}]+[\text{Aspartate\{Mitochondria\}}]\cdot[\text{Glutamate\{Cytosol\}}]+0.0000001}\right)$$

$$\frac{d\left([\text{Aspartate\{Cytosol\}}]\cdot V_{\text{Cytosol}}\right)}{d\,t} = -V_{\text{Cytosol}}\cdot\left(\text{kcat}_{\text{("(MAS) GOT1")}}\cdot[\text{enz\_GOT1}]\cdot\frac{[\text{Aspartate\{Cytosol\}}]\cdot[\text{"a-KG\{Cytosol\}"}]}{\text{KmB}_{\text{("(MAS) GOT1")}}\cdot[\text{Aspartate\{Cytosol\}}]+\text{KmA}_{\text{("(MAS) GOT1")}}\cdot[\text{"a-KG\{Cytosol\}"}]+[\text{Aspartate\{Cytosol\}}]\cdot[\text{"a-KG\{Cytosol\}"}]+0.0000001}\right)$$

$$+V_{\text{Mitochondria}}\cdot\left(\text{kcat}_{\text{("(MAS) A-G Transport")}}\cdot[\text{enz\_SLC1A3}]\cdot\frac{[\text{Aspartate\{Mitochondria\}}]\cdot[\text{Glutamate\{Cytosol\}}]}{\text{KmB}_{\text{("(MAS) A-G Transport")}}\cdot[\text{Aspartate\{Mitochondria\}}]+\text{KmA}_{\text{("(MAS) A-G Transport")}}\cdot[\text{Glutamate\{Cytosol\}}]+[\text{Aspartate\{Mitochondria\}}]\cdot[\text{Glutamate\{Cytosol\}}]+0.0000001}\right)$$

$$\frac{d\left([\text{Acyl\_Carnitine\{Cytosol\}}]\cdot V_{\text{Cytosol}}\right)}{d\,t} = +V_{\text{Cytosol}}\cdot\left(\text{kcat}_{\text{("(FAO) CPT1a")}}\cdot[\text{enz\_CPT1a}]\cdot\frac{[\text{"Acyl-CoA\{Cytosol\}"}]}{\text{Km}_{\text{("(FAO) CPT1a")}}+[\text{"Acyl-CoA\{Cytosol\}"}]}\right)$$

$$-V_{\text{Cytosol}}\cdot\left(\text{kcat}_{\text{("(FAO) CACT")}}\cdot[\text{enz\_CACT}]\cdot\frac{[\text{Acyl\_Carnitine\{Cytosol\}}]}{\text{Km}_{\text{("(FAO) CACT")}}+[\text{Acyl\_Carnitine\{Cytosol\}}]}\right)$$

$$[\text{enz\_TAL}] = \text{Naive}\cdot 1.87\text{E-}10+\text{Th1}\cdot 1.26\text{E-}09+\text{Th2}\cdot 3.34\text{E-}10+\text{Th17}\cdot 3.87\text{E-}10+\text{Th22}\cdot 1.00\text{E-}10+\text{Treg}\cdot 3.13\text{E-}10$$

$$[\text{enz\_ComplexI}] = \text{Naive}\cdot 5.92\text{E-}07+\text{Th1}\cdot 8.19\text{E-}07+\text{Th2}\cdot 8.36\text{E-}07+\text{Th17}\cdot 8.50\text{E-}07+\text{Th22}\cdot 7.81\text{E-}07+\text{Treg}\cdot 1.03\text{E-}06$$

$$\frac{d\left([\text{H\{Mitochondria\}}]\cdot V_{\text{Mitochondria}}\right)}{d\,t} = -6\cdot V_{\text{Mitochondria}}\cdot\left(\text{kcat}_{\text{("(OXPHOS) II-IV")}}\cdot[\text{enz\_ComplexII}]\cdot\frac{[\text{FADH2\_S\_OXPHOS}]}{\text{Km}_{\text{("(OXPHOS) II-IV")}}+[\text{FADH2\_S\_OXPHOS}]}\cdot\frac{[\text{H\{Mitochondria\}}]}{\text{Km2}_{\text{("(OXPHOS) II-IV")}}+[\text{H\{Mitochondria\}}]}\right)$$

$$+V_{\text{Mitochondria}}\cdot\left(\text{Vf}_{\text{("(OXPHOS) H pool")}}-[\text{H\{Mitochondria\}}]\cdot\text{Vr}_{\text{("(OXPHOS) H pool")}}\right)$$

$$-10\cdot V_{\text{Mitochondria}}\cdot\left(\text{kcat}_{\text{("(OXPHOS) I-IV")}}\cdot[\text{enz\_ComplexI}]\cdot\frac{[\text{NADH\_S\_OXPHOS}]}{\text{Km}_{\text{("(OXPHOS) I-IV")}}+[\text{NADH\_S\_OXPHOS}]}\cdot\frac{[\text{H\{Mitochondria\}}]}{\text{Km2}_{\text{("(OXPHOS) I-IV")}}+[\text{H\{Mitochondria\}}]}\right)$$

$$+4\cdot V_{\text{Intermembrane}}\cdot\left(0\cdot\left([\text{H\{Intermembrane\}}]+[\text{H\{Intermembrane\}}]+[\text{H\{Intermembrane\}}]+[\text{H\{Mitochondria\}}]+[\text{H\{Mitochondria\}}]+[\text{H\{Mitochondria\}}]\right)+[\text{enz\_ATPase}]\cdot\text{kcat}_{\text{("(OXPHOS) V")}}\cdot\left(\text{Vf}_{\text{("(OXPHOS) V")}}\cdot[\text{H\{Intermembrane\}}]\cdot\frac{[\text{ADP\_S\{Mitochondria\}}]}{[\text{ADP\_S\{Mitochondria\}}]+\text{Km1}_{\text{("(OXPHOS) V")}}}-\text{Vr}_{\text{("(OXPHOS) V")}}\cdot[\text{H\{Mitochondria\}}]\cdot\frac{[\text{ATP\_P\_OXPHOS}]}{[\text{ATP\_P\_OXPHOS}]+\text{Km2}_{\text{("(OXPHOS) V")}}}\right)\right)$$

$$[\text{enz\_RPE}] = \text{Naive}\cdot 1.89\text{E-}08+\text{Th1}\cdot 1.38\text{E-}08+\text{Th2}\cdot 1.27\text{E-}08+\text{Th17}\cdot 1.02\text{E-}08+\text{Th22}\cdot 1.15\text{E-}08+\text{Treg}\cdot 1.77\text{E-}08$$

$$[\text{enz\_MPC}] = \text{Naive}\cdot 7.00\text{E-}08+\text{Th1}\cdot 1.29\text{E-}07+\text{Th2}\cdot 6.89\text{E-}08+\text{Th17}\cdot 8.85\text{E-}08+\text{Th22}\cdot 1.19\text{E-}07+\text{Treg}\cdot 1.91\text{E-}07$$

$$\frac{d\left([\text{Glutamate\{Cytosol\}}]\cdot V_{\text{Cytosol}}\right)}{d\,t} = +V_{\text{Cytosol}}\cdot\left(\text{kcat}_{\text{("(MAS) GOT1")}}\cdot[\text{enz\_GOT1}]\cdot\frac{[\text{Aspartate\{Cytosol\}}]\cdot[\text{"a-KG\{Cytosol\}"}]}{\text{KmB}_{\text{("(MAS) GOT1")}}\cdot[\text{Aspartate\{Cytosol\}}]+\text{KmA}_{\text{("(MAS) GOT1")}}\cdot[\text{"a-KG\{Cytosol\}"}]+[\text{Aspartate\{Cytosol\}}]\cdot[\text{"a-KG\{Cytosol\}"}]+0.0000001}\right)$$

$$-V_{Mitochondria}\cdot\left(kcat_{\text{"(MAS) A-G Transport"}}\cdot[\text{enz\_SLC1A3}]\cdot\frac{[\text{Aspartate\{Mitochondria\}}]\cdot[\text{Glutamate\{Cytosol\}}]}{KmB_{\text{"(MAS) A-G Transport"}}\cdot[\text{Aspartate\{Mitochondria\}}]+KmA_{\text{"(MAS) A-G Transport"}}\cdot[\text{Glutamate\{Cytosol\}}]+[\text{Aspartate\{Mitochondria\}}]\cdot[\text{Glutamate\{Cytosol\}}]+0.0000001}\right)$$

$$\frac{d\left([\text{H\{Intermembrane\}}]\cdot V_{Intermembrane}\right)}{dt} = +6\cdot V_{Mitochondria}\cdot\left(kcat_{\text{"(OXPHOS) II-IV"}}\cdot[\text{enz\_ComplexII}]\cdot\frac{[\text{FADH2\_S\_OXPHOS}]}{Km_{\text{"(OXPHOS) II-IV"}}+[\text{FADH2\_S\_OXPHOS}]}\cdot\frac{[\text{H\{Mitochondria\}}]}{Km2_{\text{"(OXPHOS) II-IV"}}+[\text{H\{Mitochondria\}}]}\right)$$

$$+10\cdot V_{Mitochondria}\cdot\left(kcat_{\text{"(OXPHOS) I-IV"}}\cdot[\text{enz\_ComplexI}]\cdot\frac{[\text{NADH\_S\_OXPHOS}]}{Km_{\text{"(OXPHOS) I-IV"}}+[\text{NADH\_S\_OXPHOS}]}\cdot\frac{[\text{H\{Mitochondria\}}]}{Km2_{\text{"(OXPHOS) I-IV"}}+[\text{H\{Mitochondria\}}]}\right)$$

$$-4\cdot V_{Intermembrane}\cdot\left(0\cdot([\text{H\{Intermembrane\}}]+[\text{H\{Intermembrane\}}]+[\text{H\{Intermembrane\}}]+[\text{H\{Mitochondria\}}]+[\text{H\{Mitochondria\}}]+[\text{H\{Mitochondria\}}])+[\text{enz\_ATPase}]\cdot kcat_{\text{"(OXPHOS) V"}}\cdot\left(Vf_{\text{"(OXPHOS) V"}}\cdot[\text{H\{Intermembrane\}}]\cdot\frac{[\text{ADP\_S\{Mitochondria\}}]}{[\text{ADP\_S\{Mitochondria\}}]+Km1_{\text{"(OXPHOS) V"}}}-Vr_{\text{"(OXPHOS) V"}}\cdot[\text{H\{Mitochondria\}}]\cdot\frac{[\text{ATP\_P\_OXPHOS}]}{[\text{ATP\_P\_OXPHOS}]+Km2_{\text{"(OXPHOS) V"}}}\right)\right)$$

$$[\text{enz\_ATPase}] = Naive\cdot1.07E\text{-}07+Th1\cdot1.53E\text{-}07+Th2\cdot1.25E\text{-}07+Th17\cdot1.02E\text{-}07+Th22\cdot7.93E\text{-}08+Treg\cdot2.25E\text{-}07$$

$$\frac{d\left([\text{Acyl\_Carnitine\{Mitochondria\}}]\cdot V_{Mitochondria}\right)}{dt} = -V_{Cytosol}\cdot\left(kcat_{\text{"(FAO) CPT2"}}\cdot[\text{enz\_CPT2}]\cdot\frac{[\text{Acyl\_Carnitine\{Mitochondria\}}]}{Km_{\text{"(FAO) CPT2"}}+[\text{Acyl\_Carnitine\{Mitochondria\}}]}\right)$$

$$+V_{Cytosol}\cdot\left(kcat_{\text{"(FAO) CACT"}}\cdot[\text{enz\_CACT}]\cdot\frac{[\text{Acyl\_Carnitine\{Cytosol\}}]}{Km_{\text{"(FAO) CACT"}}+[\text{Acyl\_Carnitine\{Cytosol\}}]}\right)$$

$$\frac{d\left([\text{"X-5P"}]\cdot V_{Cytosol}\right)}{dt} = +V_{Cytosol}\cdot\left(kcat_{\text{"(PPP) RPE"}}\cdot[\text{enz\_RPE}]\cdot\frac{[\text{"Ribulose-5P"}]}{Km_{\text{"(PPP) RPE"}}+[\text{"Ribulose-5P"}]}\right)$$

$$-V_{Cytosol}\cdot\left(kcat_{\text{"(PPP) TKT\_SHL7P"}}\cdot[\text{enz\_TKT}]\cdot\frac{[\text{"Ribose-5P"}]\cdot[\text{"X-5P"}]}{KmB_{\text{"(PPP) TKT\_SHL7P"}}\cdot[\text{"Ribose-5P"}]+KmA_{\text{"(PPP) TKT\_SHL7P"}}\cdot[\text{"X-5P"}]+[\text{"Ribose-5P"}]\cdot[\text{"X-5P"}]+0.0000001}\right)$$

$$-V_{Cytosol}\cdot\left(kcat_{\text{"(PPP) TKT\_F6-P"}}\cdot[\text{enz\_TKT}]\cdot\frac{[\text{"X-5P"}]\cdot[\text{"E-5P"}]}{KmB_{\text{"(PPP) TKT\_F6-P"}}\cdot[\text{"X-5P"}]+KmA_{\text{"(PPP) TKT\_F6-P"}}\cdot[\text{"E-5P"}]+[\text{"X-5P"}]\cdot[\text{"E-5P"}]+0.0000001}\right)$$

$$\frac{d\left([\text{ADP\_P\_SUCL}]\cdot V_{Mitochondria}\right)}{dt} = +V_{Mitochondria}\cdot\left(kcat_{\text{"(TCA) SUCL\_R"}}\cdot[\text{enz\_SUCL}]\cdot\frac{[\text{Succinate}]}{Km_{\text{"(TCA) SUCL\_R"}}+[\text{Succinate}]}\right)$$

$$[\text{enz\_SLC25A11}] = \text{Naive}\cdot 9.28\text{E-}08 + \text{Th1}\cdot 9.36\text{E-}08 + \text{Th2}\cdot 8.56\text{E-}08 + \text{Th17}\cdot 7.80\text{E-}08 + \text{Th22}\cdot 6.50\text{E-}08 + \text{Treg}\cdot 1.01\text{E-}07$$

$$\frac{d\left(["\text{PDH-P}"]\cdot V_{\text{Cytosol}}\right)}{d\,t} = +V_{\text{Mitochondria}}\cdot\left(\text{Vf}_{(\text{"PDH phosphorylation-A"})}\cdot[\text{enz\_PDH}]\cdot\frac{["\text{enz\_PDK-A}"]^{n1_{(\text{"PDH phosphorylation-A"})}}}{["\text{enz\_PDK-A}"]^{n1_{(\text{"PDH phosphorylation-A"})}} + \text{ka1}_{(\text{"PDH phosphorylation-A"})}^{n1_{(\text{"PDH phosphorylation-A"})}}} - \text{Vr}_{(\text{"PDH phosphorylation-A"})}\cdot["\text{PDH-P}"]\right)$$

$$[\text{enz\_PC}] = \begin{cases} 1\text{e-}13 & \text{Naive}\cdot 1.12\text{E-}08 + \text{Th1}\cdot 4.74\text{E-}09 + \text{Th2}\cdot 7.05\text{E-}09 + \text{Th17}\cdot 5.21\text{E-}09 + \text{Th22}\cdot 4.00\text{E-}09 + \text{Treg}\cdot 6.19\text{E-}09 - ["\text{enz\_PC-A}"] <= 1\text{e-}13, \\ \text{Naive}\cdot 1.12\text{E-}08 + \text{Th1}\cdot 4.74\text{E-}09 + \text{Th2}\cdot 7.05\text{E-}09 + \text{Th17}\cdot 5.21\text{E-}09 + \text{Th22}\cdot 4.00\text{E-}09 + \text{Treg}\cdot 6.19\text{E-}09 - ["\text{enz\_PC-A}"] & \text{else,} \end{cases}$$

$$\frac{d\left([\text{FAD\_P\_SDH}]\cdot V_{\text{Mitochondria}}\right)}{d\,t} = +V_{\text{Cytosol}}\cdot\left(\text{kcat}_{(\text{"(TCA) SDH\_R"})}\cdot[\text{enz\_SDH}]\cdot\frac{[\text{Fumarate}]}{\text{Km}_{(\text{"(TCA) SDH\_R"})} + [\text{Fumarate}]}\right)$$

$$[\text{enz\_MCT4}] = \text{mct4\_activity}\cdot(\text{Naive}\cdot 3.57\text{E-}08 + \text{Th1}\cdot 1.22\text{E-}07 + \text{Th2}\cdot 1.05\text{E-}07 + \text{Th17}\cdot 3.08\text{E-}08 + \text{Th22}\cdot 1.42\text{E-}07 + \text{Treg}\cdot 7.96\text{E-}08)$$

$$[\text{enz\_PDK}] = \begin{cases} 1\text{e-}13 & \text{Naive}\cdot 8.95\text{E-}08 + \text{Th1}\cdot 6.29\text{E-}08 + \text{Th2}\cdot 6.4\text{E-}08 + \text{Th17}\cdot 9.18\text{E-}08 + \text{Th22}\cdot 3.05\text{E-}07 + \text{Treg}\cdot 1.89\text{E-}07 - ["\text{PDK-P}"] - ["\text{enz\_PDK-A}"] <= 1\text{e-}13, \\ \text{Naive}\cdot 8.95\text{E-}08 + \text{Th1}\cdot 6.29\text{E-}08 + \text{Th2}\cdot 6.4\text{E-}08 + \text{Th17}\cdot 9.18\text{E-}08 + \text{Th22}\cdot 3.05\text{E-}07 + \text{Treg}\cdot 1.89\text{E-}07 - ["\text{PDK-P}"] - ["\text{enz\_PDK-A}"] & \text{else,} \end{cases}$$

$$[\text{enz\_PCK1}] = \text{Naive}\cdot 1.00\text{E-}10 + \text{Th1}\cdot 1.00\text{E-}10 + \text{Th2}\cdot 1.00\text{E-}10 + \text{Th17}\cdot 1.00\text{E-}10 + \text{Th22}\cdot 1.00\text{E-}10 + \text{Treg}\cdot 1.00\text{E-}10$$

$$\frac{d\left([\text{ADP\_P\_PC}]\cdot V_{\text{Mitochondria}}\right)}{d\,t} = +V_{\text{Mitochondria}}\cdot\left(\text{kcat}_{(\text{"(TCA) PC-A"})}\cdot["\text{enz\_PC-A}"]\cdot\frac{[\text{Pyruvate\{Mitochondria\}}]}{\text{Km}_{(\text{"(TCA) PC-A"})} + [\text{Pyruvate\{Mitochondria\}}]}\right)$$
$$+V_{\text{Mitochondria}}\cdot\left(\text{kcat}_{(\text{"(TCA) PC"})}\cdot[\text{enz\_PC}]\cdot\frac{[\text{Pyruvate\{Mitochondria\}}]}{\text{Km}_{(\text{"(TCA) PC"})} + [\text{Pyruvate\{Mitochondria\}}]}\right)$$

$$[\text{enz\_ME2}] = \text{Naive}\cdot 6.61\text{E-}08 + \text{Th1}\cdot 5.56\text{E-}08 + \text{Th2}\cdot 6.58\text{E-}08 + \text{Th17}\cdot 5.42\text{E-}08 + \text{Th22}\cdot 4.75\text{E-}08 + \text{Treg}\cdot 6.80\text{E-}08$$

$$\frac{d\left([\text{Lactate\{Extracellular\}}]\cdot V_{\text{Extracellular}}\right)}{d\,t} = +V_{\text{Cytosol}}\cdot\left(\text{kcat}_{(\text{"(GLY) MCT4"})}\cdot[\text{enz\_MCT4}]\cdot\frac{[\text{Lactate\{Cytosol\}}]}{\text{Km}_{(\text{"(GLY) MCT4"})} + [\text{Lactate\{Cytosol\}}]}\right)$$
$$-V_{\text{Cytosol}}\cdot\left(\text{kcat}_{(\text{"(GLY) MCT1"})}\cdot[\text{enz\_MCT1}]\cdot\frac{[\text{Lactate\{Extracellular\}}]}{\text{Km}_{(\text{"(GLY) MCT1"})} + [\text{Lactate\{Extracellular\}}]}\right)$$

$$[\text{enz\_ME1}] = \text{Naive}\cdot 1.00\text{E-}10 + \text{Th1}\cdot 8.03\text{E-}10 + \text{Th2}\cdot 1.00\text{E-}10 + \text{Th17}\cdot 1.00\text{E-}10 + \text{Th22}\cdot 1.00\text{E-}10 + \text{Treg}\cdot 1.00\text{E-}10$$

$$[\text{enz\_ACLY}] = \text{Naive}\cdot 1.11\text{E-}07 + \text{Th1}\cdot 1.93\text{E-}07 + \text{Th2}\cdot 2.02\text{E-}07 + \text{Th17}\cdot 2.19\text{E-}07 + \text{Th22}\cdot 2.16\text{E-}07 + \text{Treg}\cdot 2.52\text{E-}07$$

$$\frac{d\left([\text{"Acetyl-CoA\{Cytosol\}"}]\cdot V_{\text{Cytosol}}\right)}{d\,t} = +V_{\text{Cytosol}}\cdot\left(\text{kcat}_{(\text{"(FAS) ACLY\_F"})}\cdot[\text{enz\_ACLY}]\cdot\frac{[\text{Citrate\{Cytosol\}}]}{\text{Km}_{(\text{"(FAS) ACLY\_F"})}+[\text{Citrate\{Cytosol\}}]}\right)$$
$$-V_{\text{Cytosol}}\cdot\left(\text{kcat}_{(\text{"(FAS) ACACA"})}\cdot[\text{enz\_ACACA}]\cdot\frac{[\text{"Acetyl-CoA\{Cytosol\}"}]}{\text{Km}_{(\text{"(FAS) ACACA"})}+[\text{"Acetyl-CoA\{Cytosol\}"}]}\right)$$
$$-V_{\text{Cytosol}}\cdot\left(\text{kcat}_{(\text{"(FAS) ACLY\_R"})}\cdot[\text{enz\_ACLY}]\cdot\frac{[\text{"Acetyl-CoA\{Cytosol\}"}]\cdot[\text{OAA\{Cytosol\}}]}{\text{kia}_{(\text{"(FAS) ACLY\_R"})}\cdot\text{KmB}_{(\text{"(FAS) ACLY\_R"})}+\text{KmB}_{(\text{"(FAS) ACLY\_R"})}\cdot[\text{"Acetyl-CoA\{Cytosol\}"}]+\text{KmA}_{(\text{"(FAS) ACLY\_R"})}\cdot[\text{OAA\{Cytosol\}}]+[\text{"Acetyl-CoA\{Cytosol\}"}]\cdot[\text{OAA\{Cytosol\}}]}\right)$$

$$[\text{NADPH\_tot}] = [\text{NADPH\_P\_G6PD}] + [\text{NADPH\_P\_PGD}]$$

$$\frac{d\left([\text{Citrate\{Cytosol\}}]\cdot V_{\text{Cytosol}}\right)}{d\,t} = +V_{\text{Mitochondria}}\cdot\left(\text{kcat}_{(\text{"(FAS) SLC25A1\_F"})}\cdot[\text{enz\_SLC25A1}]\cdot\frac{[\text{Citrate\{Mitochondria\}}]}{\text{Km}_{(\text{"(FAS) SLC25A1\_F"})}+[\text{Citrate\{Mitochondria\}}]}\right)$$
$$-V_{\text{Cytosol}}\cdot\left(\text{kcat}_{(\text{"(FAS) ACLY\_F"})}\cdot[\text{enz\_ACLY}]\cdot\frac{[\text{Citrate\{Cytosol\}}]}{\text{Km}_{(\text{"(FAS) ACLY\_F"})}+[\text{Citrate\{Cytosol\}}]}\right)$$
$$+V_{\text{Cytosol}}\cdot\left(\text{kcat}_{(\text{"(FAS) ACLY\_R"})}\cdot[\text{enz\_ACLY}]\cdot\frac{[\text{"Acetyl-CoA\{Cytosol\}"}]\cdot[\text{OAA\{Cytosol\}}]}{\text{kia}_{(\text{"(FAS) ACLY\_R"})}\cdot\text{KmB}_{(\text{"(FAS) ACLY\_R"})}+\text{KmB}_{(\text{"(FAS) ACLY\_R"})}\cdot[\text{"Acetyl-CoA\{Cytosol\}"}]+\text{KmA}_{(\text{"(FAS) ACLY\_R"})}\cdot[\text{OAA\{Cytosol\}}]+[\text{"Acetyl-CoA\{Cytosol\}"}]\cdot[\text{OAA\{Cytosol\}}]}\right)$$
$$-V_{\text{Mitochondria}}\cdot\left(\text{kcat}_{(\text{"(FAS) SLC25A1\_R"})}\cdot[\text{enz\_SLC25A1}]\cdot\frac{[\text{Citrate\{Cytosol\}}]}{\text{Km}_{(\text{"(FAS) SLC25A1\_R"})}+[\text{Citrate\{Cytosol\}}]}\right)$$

$$[\text{enz\_SLC25A1}] = (1.23\text{E-}07\cdot\text{Th1} + 1.23\text{E-}07\cdot\text{Th2} + 1.19\text{E-}07\cdot\text{Th17} + 1.25\text{E-}07\cdot\text{Th22} + 1.39\text{E-}07\cdot\text{Treg} + 1.24\text{E-}07\cdot\text{Naive})\cdot\text{slc25a1\_on} + (1 - \text{slc25a1\_on})\cdot\text{temp}$$

$$\frac{d\left([\text{"Malonyl-CoA"}]\cdot V_{\text{Cytosol}}\right)}{d\,t} = +V_{\text{Cytosol}}\cdot\left(\text{kcat}_{(\text{"(FAS) ACACA"})}\cdot[\text{enz\_ACACA}]\cdot\frac{[\text{"Acetyl-CoA\{Cytosol\}"}]}{\text{Km}_{(\text{"(FAS) ACACA"})}+[\text{"Acetyl-CoA\{Cytosol\}"}]}\right)$$
$$-V_{\text{Cytosol}}\cdot\left(\text{kcat}_{(\text{"(FAS) FASN"})}\cdot[\text{enz\_FASN}]\cdot\frac{[\text{"Malonyl-CoA"}]}{\text{Km}_{(\text{"(FAS) FASN"})}+[\text{"Malonyl-CoA"}]}\right)$$

$$-V_{\text{Cytosol}}\cdot\left(k1_{(\text{"(AL) CACT inhibition"})}\cdot[\text{"Malonyl-CoA"}]\cdot[\text{enz\_CACT}]-k2_{(\text{"(AL) CACT inhibition"})}\cdot[\text{"enz\_CACT-M"}]\right)$$

$$[\text{enz\_ACACA}] = 1.39E\text{-}08\cdot \text{Th1} + 1.27E\text{-}08\cdot \text{Th2} + 1.42E\text{-}08\cdot \text{Th17} + 1.42E\text{-}08\cdot \text{Th22} + 2.84E\text{-}08\cdot \text{Treg} + 1.60E\text{-}08\cdot \text{Naive}$$

$$\frac{d\left([\text{"Palmitoyl-CoA"}]\cdot V_{\text{Cytosol}}\right)}{d\,t} = +V_{\text{Cytosol}}\cdot\left(kcat_{(\text{"(FAS) FASN"})}\cdot[\text{enz\_FASN}]\cdot\frac{[\text{"Malonyl-CoA"}]}{Km_{(\text{"(FAS) FASN"})}+[\text{"Malonyl-CoA"}]}\right)$$

$$[\text{enz\_FASN}] = 5.17E\text{-}07\cdot \text{Th1} + 5.53E\text{-}07\cdot \text{Th2} + 3.21E\text{-}07\cdot \text{Th17} + 4.08E\text{-}07\cdot \text{Th22} + 2.05E\text{-}07\cdot \text{Treg} + 2.21E\text{-}07\cdot \text{Naive}$$

$$\frac{d\left([\text{"enz\_PDK-A"}]\cdot V_{\text{Mitochondria}}\right)}{d\,t} = +V_{\text{Mitochondria}}\cdot\left(k1_{(\text{"(AL) PDK activation"})}\cdot[\text{"Acetyl-CoA\{Mitochondria\}"}]\cdot[\text{enz\_PDK}]-k2_{(\text{"(AL) PDK activation"})}\cdot[\text{"enz\_PDK-A"}]\right)$$

$$\frac{d\left([\text{"enz\_PC-A"}]\cdot V_{\text{Mitochondria}}\right)}{d\,t} = +V_{\text{Mitochondria}}\cdot\left(k1_{(\text{"(AL) PC activation"})}\cdot[\text{"Acetyl-CoA\{Mitochondria\}"}]\cdot[\text{enz\_PC}]-k2_{(\text{"(AL) PC activation"})}\cdot[\text{"enz\_PC-A"}]\right)$$

$$[\text{enz\_PDK\_tot}] = \text{"[enz\_PDK-A]"} + [\text{enz\_PDK}] + \text{"[PDK-P]"}$$

$$[\text{produced\_IFNg}] = 1.09E\text{-}09\cdot \text{Naive} + 2.67E\text{-}07\cdot \text{Th1} + 1.55E\text{-}09\cdot \text{Th2} + 1.39E\text{-}09\cdot \text{Th17} + 1.02E\text{-}09\cdot \text{Th22} + 0\cdot \text{Treg}$$

$$[\text{produced\_IL2}] = 1.00E\text{-}11\cdot \text{Naive} + 6.15E\text{-}09\cdot \text{Th1} + 5.17E\text{-}08\cdot \text{Th2} + 4.49E\text{-}09\cdot \text{Th17} + 3.80E\text{-}08\cdot \text{Th22} + 0\cdot \text{Treg}$$

$$[\text{produced\_IL4}] = 1.00E\text{-}11\cdot \text{Naive} + 0\cdot \text{Th1} + 1.40E\text{-}09\cdot \text{Th2} + 0\cdot \text{Th17} + 0\cdot \text{Th22} + 0\cdot \text{Treg}$$

$$[\text{produced\_IL10}] = 1.54E\text{-}10\cdot \text{Naive} + 5.90E\text{-}10\cdot \text{Th1} + 0\cdot \text{Th2} + 0\cdot \text{Th17} + 2.34E\text{-}09\cdot \text{Th22} + 2.11E\text{-}08\cdot \text{Treg}$$

$$[\text{produced\_IL12}] = 3.88E\text{-}09\cdot \text{Naive} + 7.04E\text{-}09\cdot \text{Th1} + 5.00E\text{-}09\cdot \text{Th2} + 7.28E\text{-}09\cdot \text{Th17} + 9.32E\text{-}09\cdot \text{Th22} + 2.09E\text{-}08\cdot \text{Treg}$$

$$[\text{produced\_IL17}] = 1.00E\text{-}11\cdot \text{Naive} + 0\cdot \text{Th1} + 1.83E\text{-}09\cdot \text{Th2} + 2.27E\text{-}08\cdot \text{Th17} + 3.68E\text{-}09\cdot \text{Th22} + 3.64E\text{-}09\cdot \text{Treg}$$

$$[\text{produced\_IL21}] = 1.00E\text{-}11\cdot \text{Naive} + 1.01E\text{-}09\cdot \text{Th1} + 8.86E\text{-}10\cdot \text{Th2} + 4.18E\text{-}09\cdot \text{Th17} + 3.96E\text{-}09\cdot \text{Th22} + 6.94E\text{-}10\cdot \text{Treg}$$

$$[\text{produced\_IL22}] = 1.00E\text{-}11\cdot \text{Naive} + 0\cdot \text{Th1} + 1.63E\text{-}09\cdot \text{Th2} + 4.12E\text{-}09\cdot \text{Th17} + 3.30E\text{-}08\cdot \text{Th22} + 0\cdot \text{Treg}$$

$$[\text{produced\_IL23}] = 3.32E\text{-}07\cdot \text{Naive} + 4.00E\text{-}07\cdot \text{Th1} + 3.91E\text{-}07\cdot \text{Th2} + 4.22E\text{-}07\cdot \text{Th17} + 3.11E\text{-}07\cdot \text{Th22} + 2.11E\text{-}07\cdot \text{Treg}$$

$$[\text{produced\_TGFb}] = 6.34E\text{-}07\cdot \text{Naive} + 1.64E\text{-}06\cdot \text{Th1} + 1.49E\text{-}06\cdot \text{Th2} + 1.65E\text{-}06\cdot \text{Th17} + 1.99E\text{-}06\cdot \text{Th22} + 1.71E\text{-}06\cdot \text{Treg}$$

$$[\text{produced\_TNFa}] = 6.74E\text{-}09\cdot \text{Naive} + 4.11E\text{-}08\cdot \text{Th1} + 4.37E\text{-}08\cdot \text{Th2} + 2.67E\text{-}08\cdot \text{Th17} + 5.54E\text{-}08\cdot \text{Th22} + 3.16E\text{-}08\cdot \text{Treg}$$

$$[\text{enz\_MCT1}] = \text{mct1\_activity}\cdot(\text{Naive}\cdot 3.97E\text{-}08 + \text{Th1}\cdot 4.85E\text{-}08 + \text{Th2}\cdot 6.45E\text{-}08 + \text{Th17}\cdot 7.19E\text{-}08 + \text{Th22}\cdot 7.01E\text{-}08 + \text{Treg})$$

$$\frac{d\left([\text{Lactate\_exported}]\cdot V_{\text{Cytosol}}\right)}{d\,t} = +V_{\text{Cytosol}}\cdot\left(\text{kcat}_{(\text{"(GLY) MCT4"})}\cdot[\text{enz\_MCT4}]\cdot\frac{[\text{Lactate\{Cytosol\}}]}{\text{Km}_{(\text{"(GLY) MCT4"})}+[\text{Lactate\{Cytosol\}}]}\right)$$

$$-V_{\text{Cytosol}}\cdot\left(\text{kcat}_{(\text{"(GLY) MCT1"})}\cdot[\text{enz\_MCT1}]\cdot\frac{[\text{Lactate\{Extracellular\}}]}{\text{Km}_{(\text{"(GLY) MCT1"})}+[\text{Lactate\{Extracellular\}}]}\right)$$

$$\frac{d\left([\text{PEP\{Mitochondria\}}]\cdot V_{\text{Mitochondria}}\right)}{d\,t} = +V_{\text{Mitochondria}}\cdot\left(\text{kcat}_{(\text{"(GLU) PEPCK\_M"})}\cdot[\text{enz\_PCK2}]\cdot\frac{[\text{OAA\{Mitochondria\}}]}{\text{Km}_{(\text{"(GLU) PEPCK\_M"})}+[\text{OAA\{Mitochondria\}}]}\right)$$

$$[\text{enz\_PCK2}] = \text{Naive}\cdot 1.54\text{E-}08 + \text{Th1}\cdot 2.02\text{E-}08 + \text{Th2}\cdot 2.38\text{E-}08 + \text{Th17}\cdot 2.79\text{E-}08 + \text{Th22}\cdot 2.58\text{E-}08 + \text{Treg}\cdot 4.73\text{E-}08$$

$$\text{Th1\_init} = \frac{\text{"[Tbet-P]"}^{\text{pheno\_n}}}{\text{"[Tbet-P]"}^{\text{pheno\_n}}+\text{pheno\_1\_kd}^{\text{pheno\_n}}}$$

$$\text{Th2\_init} = \frac{\text{"[GATA3-P]"}^{\text{pheno\_n}}}{\text{"[GATA3-P]"}^{\text{pheno\_n}}+\text{pheno\_2\_kd}^{\text{pheno\_n}}}$$

$$\text{Th17\_init} = \frac{\text{"[RORgt-ligand]"}^{\text{pheno\_n}}}{\text{"[RORgt-ligand]"}^{\text{pheno\_n}}+\text{pheno\_17\_kd}^{\text{pheno\_n}}}$$

$$\text{Th22\_init} = \frac{\text{"[AhR-P]"}^{\text{pheno\_n}}}{\text{"[AhR-P]"}^{\text{pheno\_n}}+\text{pheno\_22\_kd}^{\text{pheno\_n}}}$$

$$\text{Treg\_init} = \frac{\text{"[acetylated-FOXP3]"}^{\text{pheno\_n}}}{\text{"[acetylated-FOXP3]"}^{\text{pheno\_n}}+\text{pheno\_treg\_kd}^{\text{pheno\_n}}}$$

$$\text{Th1} = \begin{cases} \text{pheno\_sum} <= 1.0, & \text{Th1\_init} \\ \text{else}, & \dfrac{\text{Th1\_init}}{\text{pheno\_sum}} \end{cases}$$

$$\text{Th2} = \begin{cases} \text{pheno\_sum} <= 1.0, & \text{Th2\_init} \\ \text{else}, & \dfrac{\text{Th2\_init}}{\text{pheno\_sum}} \end{cases}$$

$$\text{Th17} = \begin{cases} \text{pheno\_sum} <= 1.0, & \text{Th17\_init} \\ \text{else}, & \dfrac{\text{Th17\_init}}{\text{pheno\_sum}} \end{cases}$$

$$\text{Th22} = \begin{cases} \text{pheno\_sum} <= 1.0, & \text{Th22\_init} \\ \text{else}, & \dfrac{\text{Th22\_init}}{\text{pheno\_sum}} \end{cases}$$

$$\text{Treg} = \begin{cases} \text{pheno\_sum} <= 1.0, & \text{Treg\_init} \\ \text{else}, & \dfrac{\text{Treg\_init}}{\text{pheno\_sum}} \end{cases}$$

$$\text{Naive} = \begin{cases} \text{pheno\_sum} < 1.0, & 1 - \text{pheno\_sum} \\ \text{else}, & 0 \end{cases}$$

$$\text{pheno\_sum} = \text{Th1\_init} + \text{Th2\_init} + \text{Th17\_init} + \text{Th22\_init} + \text{Treg\_init}$$

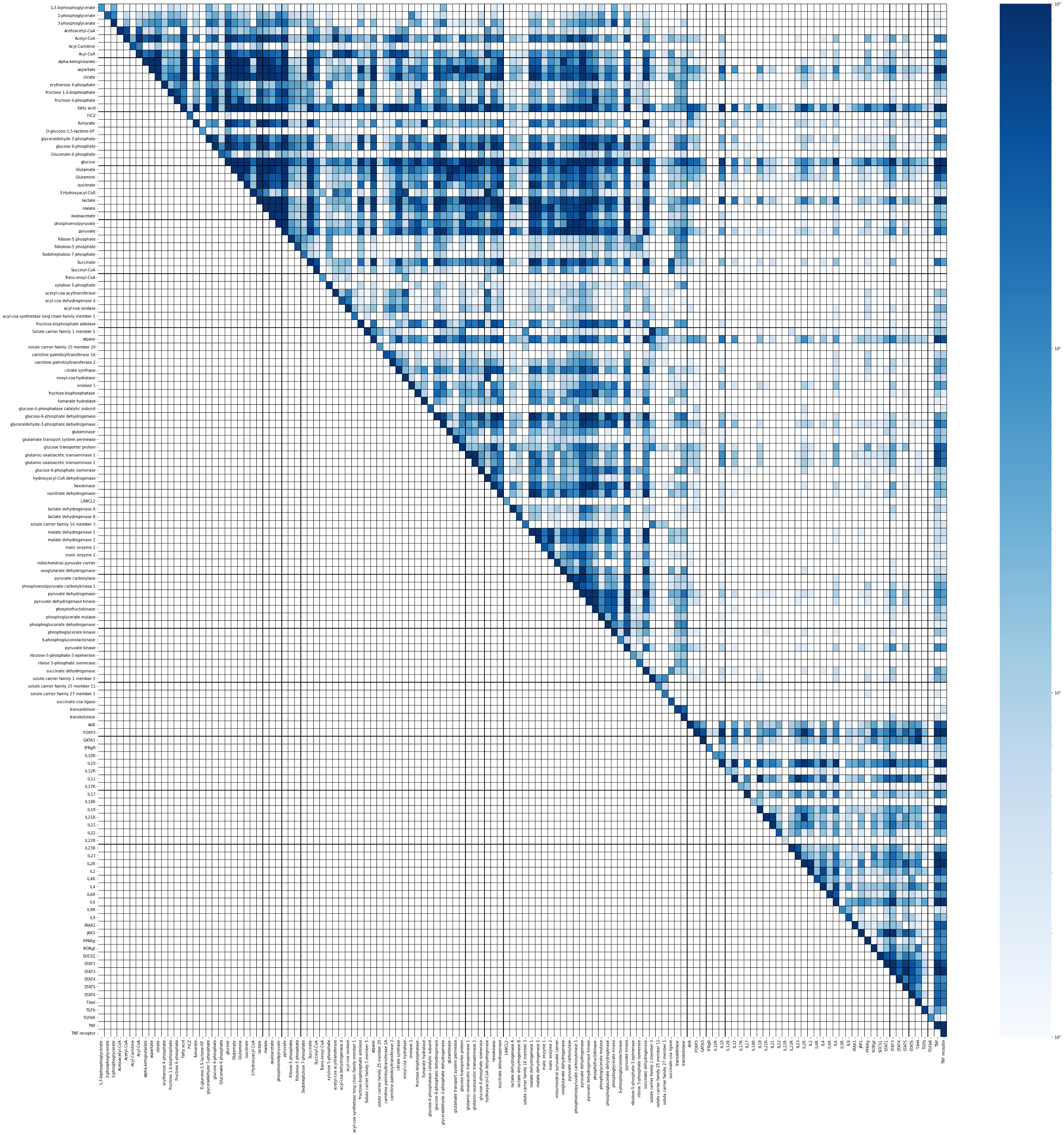

1,3-biphosphoglycerate
2-phosphoglycerate
3-phosphoglycerate
Acetoacetyl-CoA
Acetyl-CoA
Acyl-Carnitine
Acyl-CoA
alpha-ketoglutarate
aspartate
citrate
erytherose 4-phosphate
fructose 1,6-bisphosphate
fructose 6-phosphate
fatty acid
FICZ
fumarate
D-glucono-1,5-lactone-6P
glyceraldehyde 3-phosphate
glucose 6-phosphate
Gluconate-6 phosphate
glucose
Glutamate
Glutamine
isocitrate
3-Hydroxyacyl-CoA
lactate
malate
oxaloacetate
phosphoenolpyruvate
pyruvate
Ribose-5 phosphate
Ribulose-5 phosphate
Sedoheptulose 7-phosphate
Succinate
Succinyl-CoA
Trans-enoyl-CoA
xylulose 5-phosphate
acetyl-coa acyltransferase
acyl-coa dehydrogenase d
acyl-coa oxidase
acyl-coa synthetase long chain family member 1
fructose-bisphosphate aldolase
Solute carrier family 1 member 5
atpase
solute carrier family 25 member 20
carnitine palmitoyltransferase 1A
carnitine palmitoyltransferase 2
citrate synthase
enoyl-coa hydratase
enolase 1
fructose-bisphosphatase
fumarate hydratase
glucose-6-phosphatase catalytic subunit
glucose-6-phosphate dehydrogenase
glyceraldehyde-3-phosphate dehydrogenase
glutaminase
glutamate transport system permease
glucose transporter protein
glutamic-oxaloacetic transaminase 1
glutamic-oxaloacetic transaminase 2
glucose-6-phosphate isomerase
hydroxyacyl-CoA dehydrogenase
hexokinase
isocitrate dehydrogenase
LANCL2
lactate dehydrogenase A
lactate dehydrogenase B
solute carrier family 16 member 3
malate dehydrogenase 1
malate dehydrogenase 2
malic enzyme 1
malic enzyme 2
mitochondrial pyruvate carrier
oxoglutarate dehydrogenase
pyruvate carboxylase
phosphoenolpyruvate carboxykinase 1
pyruvate dehydrogenase
pyruvate dehydrogenase kinase
phosphofructokinase
phosphoglycerate mutase
phosphogluconate dehydrogenase
phosphoglycerate kinase
6-phosphogluconolactonase
pyruvate kinase
ribulose-5-phosphate-3-epimerase
ribose 5-phosphate isomerase
succinate dehydrogenase
solute carrier family 1 member 3
solute carrier family 25 member 11
solute carrier family 27 member 1
succinate-coa ligase
transaldolase
transketolase
AhR
FOXP3
GATA3
IFNgR
IL10R
IL10
IL12R
IL12
IL17R
IL17
IL18R
IL18
IL21R
IL21
IL22
IL22R
IL23R
IL23
IL2R
IL2
IL4R
IL4
IL6R
IL6
IL9R
IL9
IRAK1
JAK1
PPARg
RORgt
SOCS1
STAT1
STAT3
STAT4
STAT5
STAT6
T-bet
TGFb
TGFbR
TNF
TNF receptor
10^3
10^2
10^1
10^0

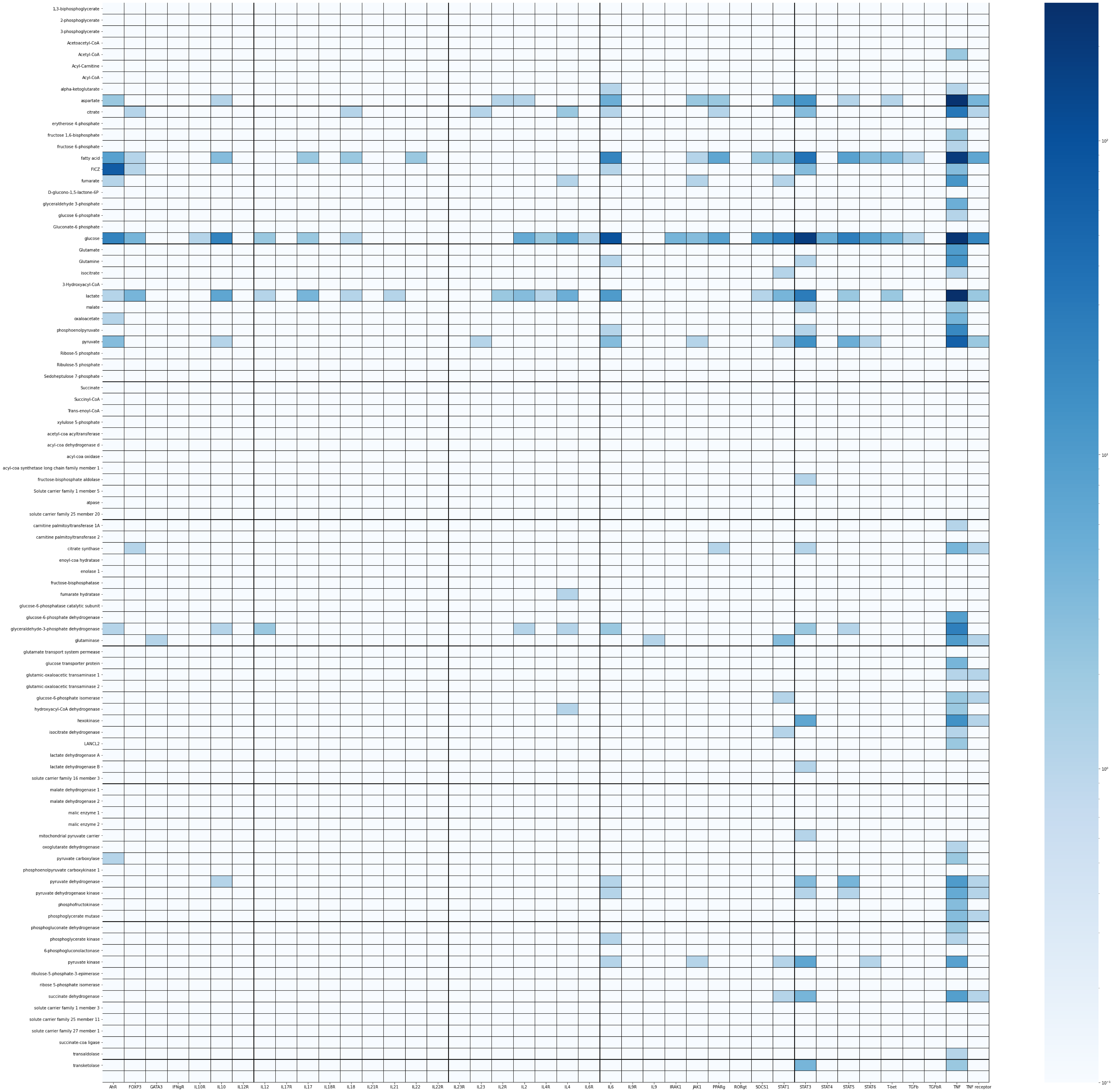
1,3-biphosphoglycerate
2-phosphoglycerate
3-phosphoglycerate
Acetoacetyl-CoA
Acetyl-CoA
Acyl-Carnitine
Acyl-CoA
alpha-ketoglutarate
aspartate
citrate
erytherose 4-phosphate
fructose 1,6-bisphosphate
fructose 6-phosphate
fatty acid
FICZ
fumarate
D-glucono-1,5-lactone-6P
glyceraldehyde 3-phosphate
glucose 6-phosphate
Gluconate-6 phosphate
glucose
Glutamate
Glutamine
isocitrate
3-Hydroxyacyl-CoA
lactate
malate
oxaloacetate
phosphoenolpyruvate
pyruvate
Ribose-5 phosphate
Ribulose-5 phosphate
Sedoheptulose 7-phosphate
Succinate
Succinyl-CoA
Trans-enoyl-CoA
xylulose 5-phosphate
acetyl-coa acyltransferase
acyl-coa dehydrogenase d
acyl-coa oxidase
acyl-coa synthetase long chain family member 1
fructose-bisphosphate aldolase
Solute carrier family 1 member 5
atpase
solute carrier family 25 member 20
carnitine palmitoyltransferase 1A
carnitine palmitoyltransferase 2
citrate synthase
enoyl-coa hydratase
enolase 1
fructose-bisphosphatase
fumarate hydratase
glucose-6-phosphatase catalytic subunit
glucose-6-phosphate dehydrogenase
glyceraldehyde-3-phosphate dehydrogenase
glutaminase
glutamate transport system permease
glucose transporter protein
glutamic-oxaloacetic transaminase 1
glutamic-oxaloacetic transaminase 2
glucose-6-phosphate isomerase
hydroxyacyl-CoA dehydrogenase
hexokinase
isocitrate dehydrogenase
LANCL2
lactate dehydrogenase A
lactate dehydrogenase B
solute carrier family 16 member 3
malate dehydrogenase 1
malate dehydrogenase 2
malic enzyme 1
malic enzyme 2
mitochondrial pyruvate carrier
oxoglutarate dehydrogenase
pyruvate carboxylase
phosphoenolpyruvate carboxykinase 1
pyruvate dehydrogenase
pyruvate dehydrogenase kinase
phosphofructokinase
phosphoglycerate mutase
phosphogluconate dehydrogenase
phosphoglycerate kinase
6-phosphogluconolactonase
pyruvate kinase
ribulose-5-phosphate-3-epimerase
ribose 5-phosphate isomerase
succinate dehydrogenase
solute carrier family 1 member 3
solute carrier family 25 member 11
solute carrier family 27 member 1
succinate-coa ligase
transaldolase
transketolase
AhR
FOXP3
GATA3
IFNgR
IL10R
IL10
IL12R
IL12
IL17R
IL17
IL18R
IL18
IL21R
IL21
IL22
IL22R
IL23R
IL23
IL2R
IL2
IL4R
IL4
IL6R
IL6
IL9R
IL9
IRAK1
JAK1
PPARg
RORgt
SOCS1
STAT1
STAT3
STAT4
STAT5
STAT6
T-bet
TGFb
TGFbR
TNF
TNF receptor
10^2
10^1
10^0
10^{-1}

Cytokine	Naïve	Th1	Th2	Th17	Th22	Treg
IFNg (M)	1.00E-13	5.00E-09	1.00E-13	1.00E-13	1.00E-13	1.00E-13
IL-2 (M)	1.00E-13	1.00E-13	1.00E-13	1.00E-13	1.00E-13	3.23E-08
IL-4 (M)	1.00E-13	1.00E-13	8.33E-10	1.00E-13	1.00E-13	1.00E-13
IL-6 (M)	1.00E-13	1.00E-13	1.00E-13	1.19E-09	1.43E-09	1.00E-13
IL-9 (M)	1.00E-13	1.00E-13	1.00E-13	5.00E-09	1.00E-13	5.00E-09
IL-10 (M)	1.00E-13	1.00E-13	1.00E-13	1.00E-13	1.00E-13	5.00E-09
IL-12 (M)	1.00E-13	3.57E-10	1.00E-13	1.00E-13	1.00E-13	1.00E-13
IL-21 (M)	1.00E-13	1.00E-13	1.00E-13	5.00E-09	1.00E-13	1.00E-13
IL-23 (M)	1.00E-13	1.00E-13	1.00E-13	4.69E-10	1.00E-13	1.00E-13
TGFb (M)	1.00E-13	1.00E-13	1.00E-13	2.08E-10	1.00E-13	2.08E-10
TNFa (M)	1.00E-13	1.00E-13	1.00E-13	1.00E-13	5.78E-10	1.00E-13

Infected D0	Infected D3	Infected D4	Infected D5	Infected D8	Infected D10
1.00E-13	6.39E-09	2.28E-10	3.24E-10	3.66E-10	4.44E-10
2.84E-09	1.62E-09	1.00E-13	1.49E-09	3.60E-09	1.43E-09
1.00E-13	1.00E-13	1.00E-13	1.00E-13	3.22E-10	1.00E-13
1.00E-13	1.73E-07	4.74E-09	3.21E-09	1.38E-10	1.00E-13
1.00E-13	1.00E-13	1.00E-13	1.00E-13	1.00E-13	1.00E-13
2.27E-10	2.70E-09	1.00E-13	1.00E-13	3.83E-10	2.21E-10
1.00E-13	3.75E-09	1.00E-13	1.07E-10	8.98E-10	3.72E-10
4.11E-11	5.44E-10	2.64E-11	9.44E-11	4.08E-11	1.00E-13
1.09E-09	2.93E-09	1.54E-09	6.63E-10	8.51E-10	9.99E-10
3.57E-08	1.02E-07	6.59E-08	4.50E-08	3.67E-08	5.21E-08
1.95E-09	6.43E-08	4.79E-08	2.75E-08	8.04E-09	4.30E-09

Supp Table 2. Parameter values for each reaction. Abbreviations for the pathway associated with each reaction precede the reaction name. They are as follows: allosteric (AL), fatty acid oxidation (FAO), fatty acid synthesis (FAS), gluconeogenesis (GLU), glycolysis (GLY), immunity (IM), malate-aspartate shuttle (MAS), oxidative phosphorylation (OXPHOS), pentose phosphate pathway (PPP), tricarboxylic acid cycle (TCA).

Reaction	Parameter	Value	Unit
((IM) IFNg receptor binding)	k1	3.60E+11	l/(mol*h)
	k2	5.4	1/h
((IM) IL10 receptor binding)	k1	3.60E+11	l/(mol*h)
	k2	1.43942	1/h
((IM) IL12 receptor binding)	k1	3.60E+11	l/(mol*h)
	k2	180	1/h
((IM) IL17 receptor binding)	k1	3.60E+11	l/(mol*h)
	k2	1800	1/h
((IM) IL18 receptor binding)	k1	3.60E+11	l/(mol*h)
	k2	5.26604	1/h
((IM) IL21 receptor binding)	k1	3.60E+11	l/(mol*h)
	k2	25.2	1/h
((IM) IL23 receptor binding)	k1	3.60E+11	l/(mol*h)
	k2	1368	1/h
((IM) IL2 receptor binding)	k1	3.60E+11	l/(mol*h)
	k2	10800	1/h
((IM) IL4 receptor binding)	k1	3.60E+11	l/(mol*h)
	k2	55.8	1/h
((IM) IL6 receptor binding)	k1	3.60E+11	l/(mol*h)
	k2	72	1/h
((IM) TGFb receptor binding)	k1	3.60E+11	l/(mol*h)
	k2	12.6	1/h
((IM) IL9 receptor binding)	k1	3.60E+11	l/(mol*h)
	k2	36	1/h
((IM) IL22 receptor binding)	k1	3.60E+11	l/(mol*h)
	k2	0.36	1/h
((IM) TNFa receptor binding)	k1	3.60E+11	l/(mol*h)
	k2	14.4	1/h
((IM) FOXP3 activation)	Vf	81540.9	1
	ki1	4.94E-07	1
	n1	0.929973	1
	ki2	1.30E-12	1
	n2	0.883737	1
	ki3	1.47E-10	1
	n3	1.53494	1
	ki4	5.40E-08	1
	n4	1.19126	1
	c1	12125.6	1
	Vr	0.1	1/h

	ka1	2.45E-05	1
	c2	19633.7	1
	n5	1.30866	1
	ka2	1.72E-08	1
	c3	0.230021	1
	ka3	1.29E-05	1
	c4	7.20295	1
	n6	1.11051	1
	n7	1.35537	1
	ka4	2.39E-08	1
	c5	731.139	1
	n9	1.64437	1
	n8	1.32092	1
	c7	71562	1
	n11	1.3	1
	ka5	1.84E-05	1
	ka7	1.22E-07	1
	c6	4.69861	1
	n10	1.01881	1
	ka6	2.55E-09	1
((IM) STAT4 activation)	Vf	0.0602925	1
	ki1	0.000126657	1
	n1	1.06245	1
	ki2	2.30E-07	1
	n2	1.37371	1
	c1	259.363	1
	n3	0.928568	1
	ka1	4.99E-08	1
	c2	421.43	1
	n4	1.24541	1
	ka2	3.96E-10	1
	Vr	0.1	1/h
((IM) Tbet activation)	Vf	8.63E-07	1
	ki1	7.21E-07	1
	n1	1.04225	1
	ki2	1.06E-05	1
	n2	1.15008	1
	ki3	4.92E-05	1
	n3	0.989115	1
	ki4	1.17E-08	1
	n4	1.00385	1
	c1	2394.94	1
	n5	1.10812	1
	ka1	2.87E-14	1
	c2	396.411	1
	n6	1.20378	1
	ka2	3.20E-05	1

	c3	54167.8	1
	n7	1.49994	1
	Vr	0.1	1/h
	ka3	5.90E-07	1
	n8	1.52672	1
	c4	9451.08	1
	ka4	1.17E-08	1
((IM) STAT1 activation)	Vf	5.14E+09	1
	ki1	2.24E-06	1
	n1	0.979492	1
	c1	42.7822	1
	n2	1.49896	1
	ka1	3.49E-08	1
	c2	81.8774	1
	ka2	1.73E-06	1
	c3	17.0613	1
	n3	0.813124	1
	Vr	0.1	1/h
	ka3	4.24E-17	1
	n4	1.10605	1
((IM) JAK1 activation)	Vf	7.86E+12	1
	ki1	2.27E-14	1
	n1	1.18056	1
	ki2	3.16E-07	1
	n2	1.08354	1
	c1	0.0190923	1
	n3	1.14687	1
	ka1	3.16E-09	1
	Vr	0.1	1/h
((IM) SOCS1 activation)	Vf	0.0191372	1
	c1	746.941	1
	n1	0.883939	1
	ka1	8.77E-11	1
	c2	5.86786	1
	Vr	0.1	1/h
	ka2	1.37E-06	1
	n2	1.1648	1
((IM) GATA3 activation)	Vf	5.29E+09	1
	ki1	3.41E-12	1
	n1	0.949808	1
	ki2	1.54E-11	1
	n2	1.19523	1
	ki3	1.36E-12	1
	n3	1.05095	1
	c1	695.245	1
	n4	3.20855	1
	ka1	1.29E-06	1

	c2	3.2717	1
	ka2	0.000398629	1
	n5	1.37212	1
	Vr	0.1	1/h
((IM) STAT6 activation)	Vf	0.174954	1
	ki1	6.48E-06	1
	n1	0.81557	1
	c1	135.169	1
	n2	1.75296	1
	ka1	6.74E-08	1
	Vr	0.1	1/h
((IM) STAT5 activation)	Vf	4.52465	1
	c1	3.71748	1
	n1	1.16886	1
	ka1	7.39E-07	1
	c2	2.96752	1
	n2	1.07	1
	Vr	0.1	1/h
	ka	1.41E-07	1
((IM) IRAK1 activation)	Vf	1.77444	1
	c1	1724.36	1
	n1	0.764031	1
	ka1	6.68E-08	1
	Vr	0.1	1/h
((IM) STAT3 activation)	Vf	0.000722176	1
	ki1	3.09E-06	1
	n1	1.17345	1
	c1	60.4147	1
	n2	0.758459	1
	ka1	1.72E-06	1
	c2	141.503	1
	n3	0.764183	1
	ka2	9.40E-12	1
	c3	459.665	1
	n4	4.36602	1
	ka3	8.37E-07	1
	c4	135.787	1
	n5	1.49931	1
	ka4	9.50E-07	
	c5	101.776	1
	n6	1.08064	1
	ka5	9.28E-06	1
	c6	106.695	1
	n7	1.34844	1
	Vr	0.1	1/h
	ka6	7.04E-06	1
((IM) RORgt activation)	Vf	3.30E-07	1

	ki1	1.65E-09	1
	n1	3.1424	1
	n2	1.43967	1
	c1	257.509	1
	n3	0.884071	1
	ka1	9.30E-07	1
	c2	260287	1
	Vr	0.1	1/h
	ka2	2.89E-09	1
((IM) PPARg activation)	Vf	7.61E+06	1
	ki1	4.47E-08	1
	n1	1.24178	1
	Vr	0.1	1/h
((IM) AhR activation)	Vf	1.17163	1
	c1	0.0788843	1
	n1	0.858008	1
	ka1	1.61E-08	1
	c2	0.000562569	1
	ka2	1.08E-05	1
	n2	0.885623	1
	c3	38284.3	1
	Vr	0.1	1/h
	ka3	4.29E-08	1
	n3	1.19294	1
((IM) LANCL2 binding)	k1	1.00E+08	l/(mol*h)
	k2	230.5	1/h
((GLY) ALDO_F[merge])	kcat	19680	1/h
	Km	1.30E-05	mol/l
((GLY) PK[merge])	kcat	1.14E+06	1/h
	Km	7.00E-05	mol/l
	n1	0.872727	1
	ka	8.18E-10	1
	c1	0.25	1
((FAO) ACAA[merge])	kcat	1000	1/h
	Km	8.00E-06	mol/l
((FAO) ACSL[merge])	kcat	8064	1/h
	Km	0.000666	mol/l
((GLY) PFK[merge])	kcat	11220.5	1/h
	Km	0.000438	mol/l
((GLY) ENO_F[merge])	kcat	27360	1/h
	Km	4.30E-05	mol/l
((GLY) GAPDH_F[merge])	kcat	68760	1/h
	Km	0.000495	mol/l
((GLY) PGK_F[merge])	kcat	307800	1/h
	Km	0.0064	mol/l
((GLY) GLUT[merge])	kcat	543600	1/h
	Km	0.002	mol/l

((GLY) PGAM_F[merge])	kcat	2.27E+06	1/h
	Km	0.00038	mol/l
((GLY) GPI_F[merge])	kcat	5.08E+06	1/h
	Km	0.00165	mol/l
((GLY) HK[merge])	kcat	240120	1/h
	Km	0.006	mol/l
((GLY) LDHA[merge])	kcat	1.62E+06	1/h
	Km	3.00E-05	mol/l
((FAO) ECH[merge])	kcat	138000	1/h
	Km	0.0005	mol/l
((FAO) FATP[merge])	kcat	108000	1/h
	Km	9.45E-05	mol/l
((FAO) CPT2[merge])	kcat	1566	1/h
	Km	4.31E-05	mol/l
((FAO) HADH[merge])	kcat	69840	1/h
	Km	1.90E-05	mol/l
((FAO) ACAD[merge])	kcat	7200	1/h
	Km	2.80E-06	mol/l
((TCA) MDH2)	kcat	283738	1/h
	Km	0.00353	mol/l
((FAO) CPT1a[merge])	kcat	1566.1	1/h
	Km	4.31E-05	mol/l
((TCA) IDH)	kcat	105336	1/h
	Km	0.0022	mol/l
((TCA) FH_F)	kcat	1.55E+06	1/h
	Km	0.00038	mol/l
((FAO) CACT[merge])	kcat	10800	1/h
	Km	0.0005	mol/l
((PPP) G6PD[merge])	kcat	727200	1/h
	Km	4.57E-05	mol/l
((TCA) PDH)	kcat	10800	1/h
	Km	3.00E-05	mol/l
	n1	2	1
	ka	1.38E-09	mol/l
	c1	1.40838	1
((TCA) SDH_F)	kcat	2520	1/h
	Km	0.0008	mol/l
((PPP) RPI[merge])	kcat	248400	1/h
	Km	0.00123	mol/l
((TCA) OGDH)	kcat	108000	1/h
	Km	0.00015	mol/l
((TCA) CS)	kcat	64800	1/h
	KmB	2.00E-05	mol/l
	KmA	7.00E-06	mol/l
((TCA) ACO)	kcat	3070.8	1/h
	Km	0.00017	mol/l
((GLU) LDHB[merge])	kcat	779998	1/h

	Km	5.50E-05	mol/l
((GLU) G6PC[merge])	kcat	36000	1/h
	Km	0.001288	mol/l
((Mito Transport) MPC_F[merge])	kcat	10000	1/h
	Km	5.00E-05	mol/l
((TCA) SUCL_F)	kcat	723600	1/h
	Km	2.21E-05	mol/l
((GLU) PEPCK[merge])	kcat	133200	1/h
	Km	1.20E-05	mol/l
((PPP) RPE[merge])	kcat	1.01E+06	1/h
	Km	0.0002	mol/l
((PPP) PGD[merge])	kcat	10416.1	1/h
	Km	2.60E-05	mol/l
((PPP) PGL[merge])	kcat	51480	1/h
	Km	0.00083	mol/l
((PPP) TKT_SHL7P[merge])	kcat	203760	1/h
	KmB	2.60E-05	mol/l
	KmA	2.60E-05	mol/l
((PPP) TKT_F6-P[merge])	kcat	610914	1/h
	KmB	0.15	mol/l
	KmA	0.15	mol/l
((GLY) ALDO_R[merge])	kcat	37020	1/h
	Km	7.50E-05	mol/l
((GLY) GAPDH_R[merge])	kcat	264816	1/h
	Km	3.60E-05	mol/l
((GLU) FBP[merge])	kcat	7488	1/h
	Km	2.10E-06	mol/l
((TCA) ME)	kcat	748440	1/h
	Km	0.00309	mol/l
((PPP) TAL[merge])	kcat	48873.1	1/h
	KmB	0.004	mol/l
	KmA	0.004	mol/l
((GLY) GPI_R[merge])	kcat	1.00E+07	1/h
	Km	0.00262	mol/l
((GLY) PGK_R[merge])	kcat	2.16E+06	1/h
	Km	0.005	mol/l
((Glutaminolysis) ASCT2_F[merge])	kcat	108000	1/h
	Km	0.0004	mol/l
((TCA) SUCL_R)	kcat	255600	1/h
	Km	0.0009	mol/l
((MAS) GOT2[merge])	kcat	14400	1/h
	KmB	0.00116	mol/l
	KmA	7.00E-05	mol/l
((GLY) PGAM_R[merge])	kcat	1.44E+06	1/h
	Km	0.00141	mol/l
((GLY) ENO_R[merge])	kcat	198000	1/h
	Km	0.000102	mol/l

((MAS) GOT1[merge])	kcat	297360	1/h
	KmB	0.0001	mol/l
	KmA	0.0025	mol/l
((TCA) FH_R)	kcat	1.04E+06	1/h
	Km	0.005	mol/l
((TCA) SDH_R)	kcat	2.37E+06	1/h
	Km	4.30E-05	mol/l
((MAS) MDH1[merge])	kcat	446.4	1/h
	Km	0.0044	mol/l
((MAS) M-aKG Transport[merge])	kcat	108000	1/h
	KmB	0.0005	mol/l
	KmA	0.0005	mol/l
((MAS) A-G Transport[merge])	kcat	108000	1/h
	KmB	0.0005	mol/l
	KmA	0.0005	mol/l
((Glutaminolysis) GLS_F[merge])	kcat	72000	1/h
	Km	0.0004	mol/l
((Glutaminolysis) GLS_R[merge])	kcat	72000	1/h
	Km	0.0004	mol/l
((Glutaminolysis) GLUD_R[merge])	kcat	374400	1/h
	Km	0.00674	mol/l
((Glutaminolysis) GLUD_F[merge])	kcat	234000	1/h
	Km	0.003	mol/l
((OXPHOS) II-IV[merge])	kcat	700	1/h
	Km	4.30E-05	mol/l
	Km2	1.00E-08	mol/l
((OXPHOS) H pool[merge])	Vf	1	mol/(h*l)
	Vr	1000	1/h
((Glutaminolysis) ME[merge])	kcat	748440	1/h
	Km	0.00309	mol/l
((TCA) PC)	kcat	214200	1/h
	Km	0.0005	mol/l
((GLY) GLUT_R[merge])	kcat	543600	1/h
	Km	0.002	mol/l
((Glutaminolysis) ASCT2_R[merge])	kcat	108000	1/h
	Km	0.0004	mol/l
((FAO) FATP_R[merge])	kcat	108000	1/h
	Km	9.45E-05	mol/l
((OXPHOS) I-IV[merge])	kcat	700	1/h
	Km	9.00E-06	mol/l
	Km2	1.00E-08	mol/l
((OXPHOS) V[merge])	kcat	5000	1
	Vf	10	1
	Km1	1.00E-07	mol/l
	Vr	1	1
	Km2	1.00E-07	mol/l
((GLY) MCT[merge])	kcat	8820	1/h

	Km	0.0007	mol/l
((IM) NADPH_G6PD_deg)	k1	0.1	1/h
((IM) NADPH_P6D_deg)	k1	0.1	1/h
((FAS) SLC25A1_F)	kcat	4897.25	1/h
	Km	3.66E-06	mol/l
((FAS) ACLY_F)	kcat	1771.2	1/h
	Km	1.10E-05	mol/l
((FAS) ACACA)	kcat	6459.29	1/h
	Km	0.000173551	mol/l
((FAS) FASN)	kcat	36.4	1/h
	Km	2.60E-05	mol/l
((FAS) ACLY_R)	kcat	108	1/h
	kia	0.14	mol/l
	KmB	1.30E-06	mol/l
	KmA	0.0255	mol/l
((FAS) SLC25A1_R)	kcat	1931.76	1/h
	Km	7.50E-06	mol/l
((AL) PDK activation)	k1	3.60E+11	l/(mol*h)
	k2	7.00E+06	1/h
((AL) PC activation)	k1	3.60E+11	l/(mol*h)
	k2	6.00E+08	1/h
((TCA) PC-A)	kcat	2.11E+06	1/h
	Km	0.0005	mol/l
((AL) CACT inhibition)	k1	3.60E+11	l/(mol*h)
	k2	10	1/h
(PDH phosphorylation-A)	Vf	986.279	1
	n1	1.53858	1
	ka1	5.07E-07	1
	Vr	0.986279	1/h
